# Predictors of Child-to-Parent Violence in Adolescence: A Systematic Review

**DOI:** 10.3390/children13060807

**Published:** 2026-06-11

**Authors:** Lara Mendes, Rita dos Santos, Cátia Martins, Cláudia Carmo, Marta Brás, Cristina Nunes

**Affiliations:** 1 Campus de Gambelas, Universidade do Algarve, 8005-139 Faro, Portugal; 2 Campus de Gambelas, University Research Center in Psychology, Universidade do Algarve, 8005-139 Faro, Portugal; rasantos@ualg.pt (R.d.S.); csmartins@ualg.pt (C.M.); cgcarmo@ualg.pt (C.C.); mbras@ualg.pt (M.B.)

**Keywords:** adolescents, child-to-parent violence, personality, psychopathology, systematic literature review, victimization

## Abstract

**Highlights:**

**What are the main findings?**

- Child-to-parent violence (CPV) is strongly related to early and ongoing trajectories of family victimization, including direct or vicarious violence perpetrated by parents, which encourages the learning of aggressive behaviors, the legitimization of violence, and the development of feelings of anger and resentment towards the parent.
- Exposure to victimization in childhood compromises emotional, socio-cognitive and personality development, with CPV being associated with high levels of psychopathology, namely psychopathic traits, antisocial personality disorder, substance use and multiple clinical comorbidities.

**What are the implications of the main findings?**

- CPV is multifactorial and occurs in contexts of early family victimization, requiring early and multidisciplinary interventions in the context of trauma.
- Prioritize longitudinal and qualitative studies to understand trajectories and experiences of young people and evaluate the effectiveness of interventions, especially in Portugal.

**Abstract:**

Background/Objectives: Child-to-parent violence (CPV) refers to persistent physical, psychological, or financial violence perpetrated by children or adolescents against their parents. Although CPV has attracted increasing academic and professional attention in recent years, evidence regarding its predictors remains fragmented. This systematic literature review aimed to synthesize empirical evidence on the predictors of adolescent CPV, with a particular focus on developmental victimization, personality traits, and psychopathology. Violence refers to the intentional use of physical, psychological, or symbolic force to cause harm, control, or suffering, while aggression corresponds to intentional behavior aimed at harming another individual, which may or may not involve physical violence and is often broader and more situational. Methods: A systematic literature review was conducted in accordance with PRISMA guidelines and prospectively registered in PROSPERO (CRD42024596076). Searches were carried out in January 2025 across six electronic databases (PsycINFO, Web of Science, Scopus, PubMed, MEDLINE, and CINAHL). Empirical studies published between 2000 and 2025 examining predictors of CPV in adolescence, namely developmental victimization, personality traits, and psychopathology, were included. Methodological quality was assessed using the Mixed Methods Appraisal Tool (MMAT). Results: The search identified 862 records, of which 46 studies met the inclusion criteria and were retained for full-text analysis. Most studies were quantitative in design and published within the last 15 years, with Spain accounting for most of the empirical evidence. The findings consistently demonstrated associations between CPV and exposure to direct or vicarious family victimization, maladaptive personality traits—particularly psychopathic features—and a range of psychopathological symptoms, including substance use, mood and anxiety disorders, and neurodevelopmental conditions. Conclusions: The results support a multifactorial and developmental understanding of CPV, highlighting early victimization as a central risk context interacting with personality and mental health vulnerabilities. Limitations of the existing literature are discussed, and directions for future research are proposed, emphasizing the need for longitudinal and qualitative studies to inform prevention and intervention strategies.

## 1. Introduction

Child-to-parent Violence (CPV) is a form of domestic violence that undermines the quality of the parent-child relationship and entails significant physical and emotional consequences [1]. Despite the growing practical and scientific relevance of this issue, research on CPV remains limited in terms of conceptual integration and systematic synthesis at the international level. This gap suggests an insufficient recognition of this construct and a lack of intervention resources specifically tailored to this target population. The literature on CPV in adolescence presents significant conceptual and empirical inconsistencies. A lack of standardization in the definition of the construct stands out, with variations in the inclusion of different forms of violence (physical, psychological, and emotional), which hinders comparisons between studies. The conceptualization of CPV adopted in this review has been refined based on highly influential contributions in the field, including pioneering work [2], alongside other key systematic reviews and meta-analyses that have helped clarify the definition of the construct. Furthermore, discrepancies are observed between results obtained in clinical and community samples, as well as inconsistencies in the role of parenting styles. There is also ongoing debate regarding the specificity of CPV as a phenomenon distinct from other forms of domestic violence and externalizing behaviors, reflecting unresolved theoretical inconsistencies. Previous reviews tend not to distinguish adolescence as a specific developmental phase, nor to systematically differentiate between typologies of CPV. Additionally, there is a lack of organization of predictors according to ecological levels and less attention paid to protective factors. Moreover, previous reviews have not consistently synthesized these inconsistencies nor clearly identified which aspects remain underexplored. Many reviews still do not include the most recent evidence in a rapidly expanding field, and some do not sufficiently incorporate relevant systematic reviews and meta-analyses, which weakens the overall positioning of the literature.

According to APAV (Portuguese Association for Victim Support), domestic violence is understood as any act or omission that occurs in the context of an intimidating, family, or cohabiting relationship that inflicts physical, psychological, sexual, or economic suffering on another person, including behaviors such as mistreatment, threats, coercion, humiliation, or deprivation of liberty, regardless of whether or not there is cohabitation.

The literature distinguishes three fundamental types of CPV: physical CPV, involving bodily harm using force or objects; psychological CPV, characterized by strategies of intimidation, humiliation, and affective manipulation; and economic CPV, which comprises the destruction of property or the abuse of parental financial resources [3,4]. Additionally, CPV can be classified according to its functionality: reactive, appearing as an impulsive response to a perceived threat; or proactive, when it takes on an instrumental and deliberate character [5]. This distinction is fundamental to the theoretical debate regarding the nature of the violence: whether it should be conceptualized as a specific typology of violence or as a symptom of relational dysfunctions within the family dynamics.

Statistics on the prevalence and incidence of CPV tend to underestimate its true magnitude. In Portugal, according to the Portuguese Association for Victim Support [6], a total of 4092 cases of parents assaulted by their children were recorded between 2013 and 2018, with most of these episodes (55.2%) occurring in the shared residence. Regarding the age of the perpetrators, an incidence of 176 minors (under 18) and 585 young adults (aged 18 to 25) was reported. In terms of gender, the perpetrator profile is predominantly male (68.8%), while victims are mostly female (81.3%). Furthermore, there is evidence of heightened vulnerability among the elderly, with approximately 47% of victims being over 65 years of age [7].

Victimization has been consistently identified as a key variable in CPV research, as early exposure to violence is associated with an increased likelihood of later aggressive behaviours within the family context. Rather than representing an isolated risk factor, victimization is embedded within developmental pathways that may normalize the use of violence and impair emotion regulation and interpersonal functioning. In line with this, research has documented a victim–perpetrator overlap, whereby adolescents who engage in CPV frequently report prior experiences of victimization across their developmental trajectory [8].

Victimization within the family context can manifest in a direct form (i.e., direct aggression by parents against their children) or a vicarious form (i.e., witnessing interparental violence), which promotes the learning and internalization of violent and maladaptive relational models [9]. Early exposure to these contexts represents a risk factor that contributes to the emergence of violent personality traits, such as the justification of violence, hostility, anger, and impulsivity [9,10].

According to the literature, victims are mainly mothers, who experience higher levels of psychological violence, whereas fathers experience higher levels of physical violence. The fact that mothers are the most common victims may be explained by their role as the primary caregiver, as well as by the fact that single-parent families are predominantly headed by mothers and their child(ren) [3].

Regarding perpetrators, research indicates that they are predominantly male and more frequently engage in physical violence, whereas female perpetrators tend to engage more in psychological violence [11].

Harbin and Madden [12] argue that aggression against parents occurs mainly in situations of disagreement, such as when parents impose measures that the child finds objectionable (e.g., setting limits). In these situations, parents perceive that their usual resources (e.g., threats and punishment) are ineffective in dealing with the situation and the adolescent’s behaviour, leading them to adopt a passive attitude of reconciliation and acceptance in order to reduce family stress. However, the child interprets this conciliatory attitude as a personal victory over the parents, perceiving them as submissive and weak, and thus engages in more aggressive behaviours and makes further demands. In turn, parents reach a high level of frustration, which is reflected in harsh and hostile behaviour toward the child, again increasing family stress and the adolescent’s anger. In response to their perceived loss of control, the child adopts retaliatory behaviours through aggression, prompting parents to adopt one of two responses: either accepting a temporary loss of parental authority and returning to a submissive stance to reduce family stress; or responding with equivalent aggression toward the child. Furthermore, the Nested Ecological Model may effectively illustrate the multiple levels of perpetuation of CPV.

The study of CPV requires assessing adolescents’ personality and their functioning across different contexts (for example, school and social settings). Although there is no consensus regarding specific psychiatric diagnoses, the literature highlights a consistent pattern of emotional and behavioral dysfunction. This pattern mainly includes symptoms of anxiety and depression, as well as disruptive behaviors and difficulties in impulse control, with an impact on the overall adjustment of these youths [13,14].

Conducting a systematic review on the predictors of CPV is essential to consolidate the currently fragmented evidence and to clarify key risk factors. Despite growing scientific interest in CPV, there remains a notable scarcity of systematic reviews focusing specifically on this phenomenon in Portugal. Addressing this gap is crucial for informing prevention strategies, guiding clinical and educational interventions, and supporting the development of evidence-based policies tailored to the sociocultural context. The present research question focuses on predictors of CPV perpetrated during adolescence. However, national data referenced earlier suggest that young adults may represent a substantial proportion of perpetrators. In this context, the exclusion of the 18–25 age group requires further justification, particularly given that recent literature has increasingly extended the scope of CPV research to include emerging adulthood.

Over the last few decades, scientific literature has extensively documented the consequences of CPV on victims and the mediating role of parenting practices. However, there remains a scarcity of research focused on the perpetrator’s profile, specifically regarding the analysis of predictor variables and the mechanisms that maintain violent behavior toward parental figures. Therefore, the general objective of this study is to provide a comprehensive overview of empirical knowledge concerning the predictors of CPV perpetrated by adolescents. The following specific objectives were defined: (1) to examine the relationship between CPV and adolescents’ exposure to various types of victimization throughout their development; (2) to assess the relationship between adolescent personality traits and CPV; and (3) to investigate the relationship between adolescent psychopathology and CPV, identifying the most common disorders.

## 2. Methods

### 2.1. Type of Study

A Systematic Review aims to synthesize the current state of the art, offering a critical integration of existing knowledge while identifying research gaps and priorities for future research. This approach is particularly effective for evaluating the underlying processes and origins of behavioral phenomena through a rigorous and transparent framework [15].

The methodology and stages of an systematic review.include: (1) formulating the research question; (2) developing and registering a research protocol; (3) defining inclusion and exclusion criteria; (4) developing a search strategy and identifying information sources; (5) study selection; (6) performing a qualitative assessment of the included studies; (7) data extraction; (8) literature synthesis and interpretation of results; and (9) publication of findings [15,16].

### 2.2. Procedures

This review was conducted in accordance with the Preferred Reporting Items for Systematic Reviews and Meta-Analyses (PRISMA) guidelines [15].

#### 2.2.1. Research Question

The present study aims to address the following research question: What evidence is available in the literature regarding the predictors of child-to-parent violence perpetrated by adolescents?

The research question was structured using the SPIDER framework, as it better aligns with the exploratory and non-interventional nature of this review. The Sample (S) comprises adolescents who engage in child-to-parent violence (CPV). The Phenomenon of Interest (PI) focuses on CPV and its association with developmental victimization, individual traits, and psychopathology. The Design (D) includes empirical studies. The Evaluation (E) refers to the measurement and analysis of variables associated with CPV, namely victimization experiences, individual characteristics, and psychopathological symptoms. Finally, the Research type (R) includes quantitative, qualitative and mixed-methods studies

#### 2.2.2. Research Protocol and Registration

The research protocol was developed and evaluated a priori to minimize potential biases and ensure the study’s quality. It was registered on the PROSPERO platform, an international database for systematic reviews, on 11 October 2024 (ID: CRD42024596076). Registration on this platform ensures the transparency of the review, enhances its visibility, and prevents the duplication of research efforts [16].

#### 2.2.3. Eligibility Criteria

The following inclusion criteria regarding study publication were defined: (1) studies published in Portuguese, Spanish, or English; (2) empirical studies; (3) studies conducted in clinical, community, or judicial settings; and (4) a publication date between 2000 and 2025. Regarding the individual characteristics of the studies, the following criteria were established: (1) samples comprising adolescents of both sexes (male and female) who perpetrate violent behavior against their parents; (2) inclusion of variables related to victimization, individual traits, and psychopathology; (3) any type of study design; and (4) quantitative and mixed-methods studies.

Regarding the exclusion criteria, the following were applied: (1) case studies; (2) opinion articles; and (3) studies not addressing the variables defined in the inclusion criteria. No studies were excluded based solely on methodological quality. Instead, study quality was assessed systematically and considered during data synthesis and interpretation, in line with recommendations for reviews in heterogeneous fields.

#### 2.2.4. Information Sources and Search Strategy

The search was conducted in January 2025 across the following international bibliographic databases: PsycINFO, Web of Science, Scopus, PubMed, and EBSCO (MEDLINE and CINAHL).

The following search strategy was utilized: SU(‘filio-parental violence’ OR ‘child-to-parent abuse’ OR ‘child-to-parent violence’ OR ‘violence against parents’) AND SU(child* OR adolescen* personality) AND SU(child* OR adolescen* psychopathology).

#### 2.2.5. Study Selection

The study selection process began with the identification of records across electronic databases, followed by a manual search using the snowballing method. The first stage of screening was performed by reviewing titles and abstracts against the predefined eligibility criteria. Subsequently, potentially relevant studies were assessed via full-text review to confirm their eligibility. Studies that did not meet the inclusion criteria were excluded at this stage.

To remove duplicate records and organize the selected studies, Rayyan bibliographic management software [17] was utilized. For the full-text screening phase, Mendeley Desktop (version 1.19.8) was used. The selection process was systematically documented using the PRISMA flow diagram.

The study selection process was conducted in accordance with established systematic review procedures to ensure rigor and minimize bias. Two reviewers (L.M, C.N) initially screened all records retrieved from the databases based on titles and abstracts, applying the predefined inclusion and exclusion criteria. Subsequently, the same reviewers independently assessed the full texts of potentially eligible studies to determine final inclusion. Any discrepancies between the two reviewers were discussed and resolved through consensus; when agreement could not be reached, a third reviewer (R.S) was consulted to make the final decision. In cases requiring further clarification, a fourth (C.C.) reviewer was available to provide additional arbitration.

#### 2.2.6. Quality Assessment

The methodological quality of the included studies was assessed and documented using the Mixed Methods Appraisal Tool (MMAT; [18]). This tool evaluates the risk of bias across various study designs by distinguishing criteria for qualitative, randomized controlled, non-randomized, quantitative descriptive, and mixed-methods studies [18]. The appraisal begins with two screening questions: ‘Are there clear research questions?’ and ‘Do the collected data allow for the research question to be addressed?’. A response of ‘No’ or ‘Can’t tell’ may indicate that the study is not empirical and, therefore, cannot be appraised. For each study type, five criteria are evaluated and scored as ‘Yes’ (1 point) or ‘No’/’Can’t tell’ (0 points). The final scores range from 0 to 5, which can be expressed as percentages: 0 = 0%, 1 = 20%, 2 = 40%, 3 = 60%, 4 = 80%, and 5 = 100%. However, for mixed-methods studies, fifteen criteria are assessed—five each for the quantitative, qualitative, and mixed-methods components—with the overall quality being limited by the score of its weakest component [18].

As the instrument does not define standardized cut-off values, the categories for study characterization are established by the authors. Accordingly, the studies were classified into three levels: low quality (score below 50%), moderate quality (score between 50% and 80%), and high quality (score above 80%).

#### 2.2.7. Data Extraction

The information extracted from each study included: author(s); year of publication; the country where the research was conducted; objectives; study design; sample characteristics; data collection method(s); type of data analysis; main findings; primary limitations; and methodological quality assessment. Data extraction was conducted using a standardized form developed a priori to ensure consistency and reduce bias. The selected variables were defined to capture both methodological features and key outcomes relevant to the review objectives. The extracted data were systematically organized into structured tables to enable comparison across studies and to support a transparent and rigorous narrative synthesis.

#### 2.2.8. Synthesis of Literature and Interpretation of Results

The narrative synthesis aims to systematize the extracted evidence, enabling a coherent integration of results that address the objectives outlined in this systematic review. This synthesis was conducted following the EEECA model [19]: (a) Examine—analyzing the theme from diverse perspectives; (b) Evaluate—performing a critical analysis; (c) Establish relationships—identifying how the results correlate; (d) Compare—contrasting the findings; and (e) Argue—debating the presented perspectives.

## 3. Results

A total of 862 records were identified through the initial database search. After removing 445 duplicates, 417 records proceeded to the title screening stage. Following this analysis, 133 articles were selected for abstract screening. At this stage, 54 articles were identified for full-text assessment. However, 4 were excluded as the full text was unavailable. Consequently, 50 articles proceeded to a comprehensive full-text review, with 5 being excluded for the following reasons: (a) literature review (*n* = 1); (b) sample composed of parent victims of CPV (*n* = 1); (c) failure to meet the predefined inclusion criteria *(n* = 2); (d) absence of the variables under study (*n* = 1); and (e) poor methodological quality (*n* = 1). This process resulted in an initial selection of 44 articles.

**Figure 1:**
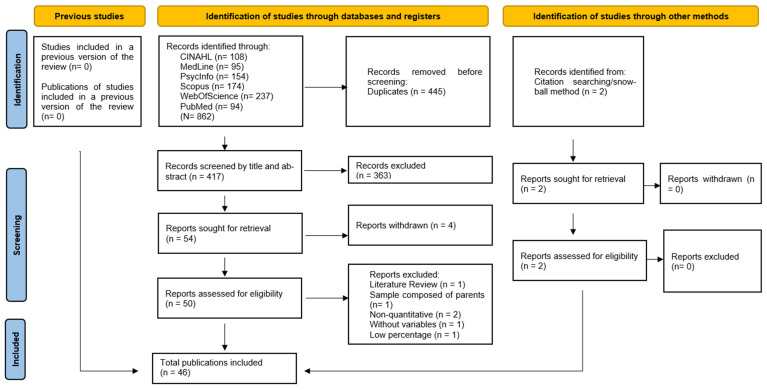
Flow diagram PRISMA.

Subsequently, the reference lists of these studies were screened using the snowballing method to identify additional relevant literature, leading to the inclusion of 2 studies. Finally, the total corpus for this systematic review comprised 46 articles. The search and selection process is illustrated in the PRISMA flow diagram (Figure 1).

**Figure 2:**
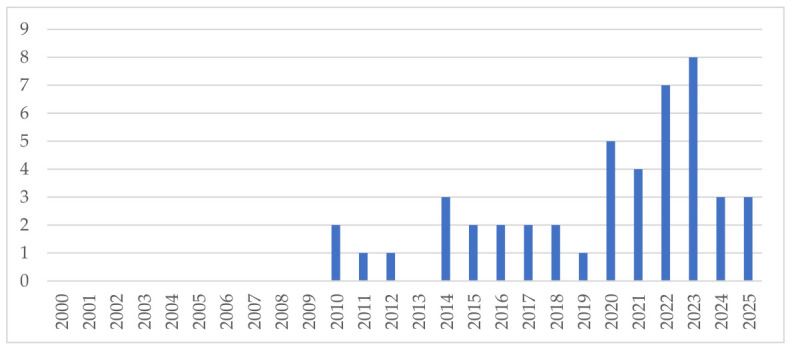
Included studies by year of publication.

The 46 included articles were published between 2000 and 2025. Specifically, no studies were identified from 2000 to 2009; however, 7 articles were published between 2010 and 2014, 9 between 2015 and 2019, and a significant majority of 30 articles between 2020 and 2025. Notably, 97.8% of the corpus was published within the last 15 years (2010–2025) (Figure 2).

A detailed analysis of outcomes is presented in Table 1 and in Supplementary Materials. Regarding the geographic distribution of the research, the studies were carried out in the following countries: Spain (*n* = 37), United States (*n* = 2), Australia (*n* = 2), Mexico (*n* = 2), France (*n* = 1), South Korea (*n* = 1), and Japan (*n* = 1).

The study samples were further analyzed based on their sample size. A large sample was defined as one exceeding 500 participants, a medium sample as one between 100 and 500 participants, and a small sample as one with fewer than 100 participants, according to the classification criteria established by the authors for the purpose of this review. According to this categorization, 22 studies included a large sample, 17 a medium sample, and 7 a small sample.

The results were synthesized into three topics aligning with the systematic reviews core variables. These were further organized into sub-topics to systematically present the main findings from the reviewed studies.

### 3.1. Victimization Throughout Adolescent Development (N = 24)

#### 3.1.1. Family Victimization

Adolescents who perpetrated CPV experienced, during childhood, both direct family violence and vicarious family violence [21,22,23,27,28,36,42,44,52]. Evidence suggests that exposure to adverse life experiences within abusive contexts is more prevalent among adolescents who committed CPV than among those involved in other types of offenses [51,58], contributing significantly to the increase in violence against parents [48,54].

Adolescents who engaged in CPV reported family functioning characterized by high levels of criticism, low family cohesion and adaptability, and limited knowledge of family history [43], reflecting a significant fragility in emotional support. Direct victimization refers to experiences in which an individual is personally and intentionally exposed to harmful acts, such as physical, psychological, or sexual violence directed at them by another person. Vicarious victimization refers to exposure to violence experienced indirectly, where an individual is affected by witnessing or being aware of violence inflicted on others (e.g., observing violence between parents or other family members), rather than being the direct target of aggression.

Regarding the most frequent types of parental violence, psychological abuse was the most prevalent, followed by verbal and physical violence [23]. Beyond direct psychological victimization [36,43], the literature identifies other influential variables in the perpetration of CPV, such as ignoring misbehavior [43]; a poor family environment, family circumstances, antisocial relationships, and inadequate supervision [62], as well as family functioning perceived as lacking cohesion and adaptability [37]. It is important to highlight that exposure to high levels of corporal punishment during childhood represents a significant predictor of CPV, with a notable tendency for youth to reproduce similar levels of violence against their parents [46].

Adolescents involved in CPV frequently present a history of parental abuse, particularly by the paternal figure [23,58]. Specifically, CPV directed toward the father is more closely associated with victimization perpetrated by him [22] and with ineffective disciplinary practices [21]. Furthermore, violence against the father has been linked to family detachment, preoccupation, and prior exposure to violence [52].

Evidence points toward gender specificity in the transmission of violence. Specifically, among boys, CPV correlates with vicarious exposure to violence from the father toward the mother, whereas in girls, these behavioral patterns are associated with witnessing violence from the mother toward the father [22]. Regarding direct victimization, girls (16%) demonstrate greater vulnerability to maternal psychological violence [23] and poly-victimization [30,32]. These findings suggest that violence directed at a specific parent is frequently associated with prior victimization perpetrated by that same parent [44], indicating the modelling and reproduction of aggressive patterns within the family environment.

Regarding the direction of CPV, direct and vicarious psychological aggression are associated with psychological CPV, while direct and vicarious physical aggression are linked to physical CPV [36]. The results highlight a predominance of reactive rather than instrumental responses [22]. The instrumental use of CPV directed at both parents is associated with the anticipation of positive consequences and the legitimization of violence [10].

Results about predictors of maternal-directed CPV identified older age, female gender, verbal-emotional violence, psychopathy, and antisocial behaviors [29], alongside family victimization [52]. Conversely, the predictors for paternal-directed CPV were younger age and psychopathy [29].

In terms of reoffending, evidence indicates a positive correlation between high recidivism rates and the perpetration of CPV [47,62].

#### 3.1.2. Interpersonal Victimization

At the interpersonal level, peer victimization, particularly in the form of bullying, has been frequently reported among adolescents exhibiting child-to-parent violence (CPV), with some evidence suggesting a higher risk of exposure among girls [32,33,58]. This factor is presented insofar as it relates to broader patterns of victimization that may be associated with CPV-related developmental pathways. Peer relationships appear to influence the practice of CPV through modelled violent behaviors [21]. However, they also represent a protective factor, buffering the impact of child abuse on the perpetration of violence against parents [54].

### 3.2. Adolescent Personality Traits (N = 32)

Exposure to family and interpersonal victimization during childhood is associated with cognitive, behavioral, and emotional difficulties in adolescence [10,28,42,58]. Dysfunctional family relationships and circumstances, characterized by low cohesion, domestic violence, maladaptive parenting styles, and inverted hierarchies [33], contribute to the development of antisocial traits, low frustration tolerance, and aggressiveness, negatively impacting adolescent self-esteem [25]. Additionally, this adverse family environment is associated with high criminogenic risk indicators and violent conduct directed toward authority figures [42].

#### 3.2.1. Emotional Intelligence

Significant challenges in emotional intelligence skills were observed, specifically regarding the difficulty in identifying, expressing, and regulating affective states, which compromises the selection of socially appropriate behaviors and reduces resilience [43,55,58].

Emotional regulation difficulties in perpetrators with cumulative adverse experiences is linked to the development of mental representations of the self and parental figures based on an absence of affection [58].

Adolescents who have committed CPV exhibit significant emotional imbalance [42], family dissatisfaction, greater psychological impairment, and an exacerbation of clinical symptoms [20].

#### 3.2.2. Psychopathic Traits

In the domain of personality, results highlight a preponderance of psychopathic traits [25]. At the interpersonal and affective levels, several traits are observed: superficiality [25]; dangerousness [25]; grandiosity [21,25,27]; manipulation [25,55]; insensitivity [25]; lack of remorse [25]; indifference [27,49]; cruelty [27]; narcissism and egocentrism [27,49], and Machiavellianism [27].

At the behavioral and social levels, young perpetrators exhibit self-harming behavior [49]; threatening attitudes [55]; intimidation [55]; low prosocial behavior [27,55]; hedonism [27,55]; controlling or dominant behaviors [23,55]; and a lack of empathy [33]. However, it is noteworthy that some studies found no direct relationship between specific empathy variables and CPV [40]. Furthermore, this maladjustment profile also manifests as social adaptation difficulties, school dropout, and workplace maladjustment [40,59].

Gender differences reveal a greater prevalence of accountability among females, contrasting with elevated levels of egocentrism, authoritarianism, and empathy challenges in males [31,35]. Specific psychological variables predict distinct behaviors: narcissism is associated with running away from home, cruelty with obscene gestures, and Machiavellianism with incurring debt [27]. Hostile sexism was identified as a double risk factor for spitting and parental theft, while benevolent sexism acted as a protective factor for the former. Finally, indifference increased the likelihood of spitting, and anger toward the mother correlated positively with physical aggression.

#### 3.2.3. Aggressiveness and Impulsivity

An aggressive personality is identified in the literature as a predictor of CPV [62], particularly regarding anger and the accessibility of aggressive responses (e.g., [10,21,55]). This challenge in anger management is transversal to various offender profiles, manifesting in both the cognitive dimension, through the justification of violence and aggressive responses within the family context [21,33], and the behavioral dimension, evidenced by school indiscipline, non-conformist attitudes, and other social conduct disturbances [24,40,60].

As a result of experienced victimization, females present more emotional problems and aggressive behaviors as a form of self-defense [30], along with a higher propensity to experience anger [30,32]. Furthermore, this group is more closely associated with the perpetration of controlling and dominant behaviors directed at the maternal figure [23].

Several authors have emphasized the role of impulsivity among adolescents who engage in CPV, particularly regarding social conflict resolution [21,26,33,56]. However, findings by Loinaz et al. [46] contradict this evidence, highlighting that no significant differences in impulsivity were observed in these youths.

#### 3.2.4. Socio-Cognitive Development and Self-Esteem

Exposure to family violence appears to foster maladaptive socio-cognitive processing, which is linked to the practice of various types of CPV, hostile attributions, emotional regulation challenges, the justification of violence, and emotional and behavioral difficulties [10,28,42].

CPV is positively associated with low self-esteem [21,28,47], serving as a predictor for a higher frequency of CPV-related violence and prior individual or family psychological interventions [41,54]. Regarding gender differences in this context, boys tend to report higher levels of self-esteem compared to girls [26,32].

#### 3.2.5. Interpersonal Skills

Challenges in social skills have been positively associated with CPV [47], reflected in lower compliance with social norms [50], school indiscipline, aversion to teachers, and social misalignment [40]. Adolescents who engage in CPV exhibit lower levels of social sensitivity, helpfulness, and collaboration, with a higher prevalence of social introversion traits [34]. Additionally, the literature suggests a negative correlation between CPV and both family and social self-concept. In the case of girls, a fragile family self-concept emerges as a significant predictor of a higher propensity for this type of violence [50].

### 3.3. Adolescent Psychopathology (N = 16)

Adolescents who engage in CPV appear to present a more severe clinical profile compared to offenders in general, with a higher prevalence of mental health conditions [34,59], emotional and behavioral challenges [40], clinical diagnoses [26], and a history of psychological and/or psychiatric interventions [26,35]. Studies suggest that CPV may function as an externalization of psychological distress, showing a positive association between distress levels and the severity of violence [50]. Psychopathological symptomatology has proven to be a highly significant predictor of CPV among youth aged 16 to 18 [57].

Regarding gender differences, girls exhibit higher levels of mental health impairment [30] along with elevated levels of psychological distress and stress [50,60]. Conversely, boys report higher scores in non-conformist attitudes [60].

#### 3.3.1. Substance Use Disorder

Substance use is a robust predictor in CPV cases [21,24,26,27,30,40,41,56,62]. It is estimated that 46.7% of youth assaulted their parents while under the influence of drugs or alcohol [26], a behavior that is particularly prevalent in late adolescence and young adulthood [59]. Boys demonstrate a higher likelihood of substance misuse [32,33,49,57].

Several studies suggest that the probability of substance misuse increases when there are prior experiences of victimization [30]. Consumption patterns may be regular or sporadic [49]. However, the study by Loinaz et al. [46] presents a diverging perspective, as it did not identify substance use as a predominant factor within this population.

#### 3.3.2. Mood Disorders

CPV perpetrators report a significant prevalence of hypomanic traits (17%) [34]. Specifically, girls present more borderline personality features [49].

Depressive symptomatology, notably high levels of personal misalignment and emotional imbalance, emerges as a central predictor of violence against parental figures [34,35,40]. Regarding gender, the literature underscores its predictive role in both boys [28] and girls [30].

It is important to note that the relationship between CPV and depressive symptomatology is not consensual. Some studies point to the absence of significant differences in depression levels within this population (e.g., [37,51]), while others evidence a negative correlation between depression and CPV perpetration [57].

#### 3.3.3. Anxiety Symptomatology

Anxiety symptomatology has been consistently associated with CPV [41], specifically generalized anxiety disorder [25], hypochondriasis [25,34], somatic symptom disorder [25], and psychasthenia [34]. From a clinical perspective, these youth exhibit personality traits characterized by ingenuity, egocentrism, exhibitionism, extraversion, and superficiality [34]. Furthermore, anxiety disorders in children who demonstrate severely overbearing behavior are also linked to social phobia, agoraphobia, and sleep disorders [35]. However, some studies have not identified a significant relationship between anxiety and CPV [37,51,57].

Regarding gender, girls present a greater vulnerability to anxiety symptomatology [32], namely, high levels of worry, sensitivity, apprehension, and tension [49].

#### 3.3.4. Attention-Deficit/Hyperactivity Disorder

Neurodevelopmental disorders are prevalent diagnoses among youth who engage in CPV [51], specifically Attention-Deficit/Hyperactivity Disorder (ADHD) [26,35,40,41,51]. The high incidence of ADHD suggests that traits characteristic of this condition, such as impulsivity, function as facilitators of aggressive conduct toward parents [51].

#### 3.3.5. Trauma and Stressor-Related Disorders

The expression of stress symptoms during adolescence may increase the likelihood of violence toward parents or other family authority figures [60]. Girls exhibit a greater vulnerability to developing post-traumatic stress symptoms [49].

#### 3.3.6. Obsessive-Compulsive Disorder

Fandiño et al. [33] and Fongaro et al. [35] highlighted the prevalence of obsessive-compulsive symptoms among some youth who engage in CPV.

#### 3.3.7. Psychotic Symptoms

Regarding psychotic symptomatology, a high prevalence of paranoid ideation is observed [25,57], expressed through sensitivity to criticism and the self-referential interpretation of others’ actions [34].

Boys show a higher propensity for hallucinations [30], whereas girls exhibit higher levels of paranoia [49].

In terms of formal clinical diagnoses, results indicate schizophrenia as a relevant pathology [25,34]. Specifically, CPV perpetrators present an incidence of symptoms 24% higher than the general offender population [34], particularly among those aged 14 to 15 [49].

#### 3.3.8. Disruptive, Impulsive-Control, and Conduct Disorders

The profile of adolescents who engage in CPV is frequently associated with disruptive behavior and impulse-control disorders [26,35]. Kennedy et al. [45] suggest that conduct disorders may be at the core of aggressive behaviors toward parents, expressed through verbal violence [21] and disruptive conduct [41]. Regarding gender differences, boys tend to exhibit a higher prevalence of behavioral issues outside the family context [40], showing a greater propensity for physical aggression and diagnoses of conduct disorder [49].

#### 3.3.9. Personality Disorders

Regarding personality disorders, the literature primarily highlights schizoid, borderline, and antisocial personality disorders [28,41,62].

Antisocial personality disorder and law-violating behaviors emerge as robust predictors of CPV [28,29,33,42,62], corroborated by evidence of high psychopathic deviation (68%) within this population [34]. Clinically, these youth exhibit marked impairment in psychopathic deviation, characterized by a lack of trust, egocentrism, an inability to learn from experience, conflicts with family or authority figures, and anger within relationships or under stressful situations [34]. However, research by Del Hoyo-Bilbao et al. [31] points to a weak positive association between psychopathic traits and psychological CPV.

Psychopathic traits show a strong correlation with CPV directed at both parents, potentially indicating emotional regulation challenges, reduced empathy, and interpersonal-affective psychopathic features, which increase the likelihood of engaging in CPV [25,29]. Boys report more antisocial characteristics and behaviors [32,49].

#### 3.3.10. Suicidal Ideation

Greater involvement in CPV appears to contribute to high levels of suicidal ideation, emotional loneliness, and alexithymia [50,61]. Regarding suicidal ideation, the literature highlights a greater vulnerability among females [30,50].

## 4. Discussion

This systematic review.aimed to synthesize the available and updated empirical evidence regarding the predictors of CPV perpetrated by adolescents. Specifically, it analyses the relationship between CPV and variables such as developmental victimization, personality profiles, and psychopathology. The relevance of this topic is grounded in the need to synthesize and clarify the international evidence on child-to-parent violence (CPV) in adolescence, given the heterogeneity of findings, definitions, and methodological approaches across studies. Importantly, this heterogeneity extends to differences in the operationalization and measurement of CPV across studies, which may partly account for inconsistencies in reported findings. While the scarcity of research conducted within the Portuguese context is noted as an additional contextual indicator, it is not intended as the primary justification for the review, which is based instead on the broader need for an integrated and updated international synthesis of CPV predictors.

Of the 46 included articles, a large part were published within the last 16 years, suggesting a recent increase in scientific interest regarding this subject. Regarding sample sizes, while they were predominantly large, they often lacked representativeness due to the specificity of the populations and contexts analyzed. This limits the generalizability of findings and may contribute to variability across studies. The reviewed articles were conducted across various countries. However, Spain accounted for most of the included studies, and this geographic concentration may have contributed to a context-specific conceptualization of CPV, potentially constraining cross-cultural comparisons and the broader applicability of existing explanatory models.

Concerning the analyzed variables, personality emerged as the most investigated construct, followed by victimization and psychopathology. However, the relative emphasis on these domains varies considerably across studies, with some prioritizing individual traits and others focusing on contextual or relational factors. Regarding study design, there is a high preponderance of quantitative methods, with comparatively limited use of qualitative approaches that could provide deeper insight into underlying mechanisms. According to the MMAT (2018) [18] qualitative assessment, the included articles demonstrated average quality and high quality.

Relatively to the topic of family victimization, developmental victimization has been identified as fundamental to understanding the practice of CPV in adolescence, as early experiences of direct and vicarious family violence were predominant factors in the emergence of CPV [21,22,23]. Nevertheless, studies differ in how developmental victimization is defined and measured (e.g., single vs. cumulative exposure), which complicates direct comparison of results. Continuous exposure to an abusive and violent family environment appears to promote the internalization of aggressive models as conflict resolution strategies [44,58], validating the mechanisms of social learning and the intergenerational transmission of violence. Therefore, developmental victimization emerges as a key contextual factor rather than a deterministic pathway. Family victimization was associated with preoccupied, avoidant, and traumatized attachment styles [58], indicating that the quality of family affective relationships mediates the expression of violent behaviors toward others. Regarding the target of CPV, the pattern of victimization is not consistent across studies, although several findings suggest that mothers are more frequently identified as targets of CPV, whereas aggression toward fathers appears in some samples but without sufficient consistency to establish a general prevalence pattern, and is significantly associated with a history of prior paternal victimization, although this pattern is not consistently observed across all samples, suggesting possible moderating effects of gender and family dynamics [22].

Bullying victimization is also positively associated with oppositional and aggressive conduct [32,58]. However, peer relationships can act as either a protective or a risk factor, depending on their quality and the presence of prosocial versus antisocial influences, reflecting the significance and complexity of personal interactions during adolescence.

Regarding personality traits, although this systematic review does not define a consistent profile, several common traits and characteristics were identified among adolescents who engaged in CPV. Psychopathic traits were the most consistently identified in the literature [21,25,27,33,55]. However, there is variability in how these traits are operationalized, ranging from brief self-report scales to more comprehensive multidimensional assessments. This is also related to a marked absence of empathy among these youth [40], reflecting challenges in emotional regulation and adaptive affective processing. Consequently, lower levels of emotional intelligence also constitute a relevant predictor of CPV [43,55], which hinders the adoption of socially appropriate behaviors and fosters the emergence of violent conduct. The frequent reference to psychopathic traits should be interpreted cautiously, as most studies rely on self-report measures rather than structured clinical assessment, which may inflate associations due to shared method variance.

Aggression and impulsivity are widely recognized as predictors of CPV [62]. Young offenders exhibit difficulties in the emotional regulation of anger, which is manifested through a high justification of violence and the adoption of aggressive behaviors during conflicts [21,33]. Importantly, some studies distinguish between reactive and proactive/instrumental aggression, suggesting potentially different underlying mechanisms, although this distinction is not consistently applied across literature. These traits may be influenced by the aggressive environment to which adolescents were exposed throughout their development. The externalization of anger is expressed through school failure [24], conflict with the law [20], and non-conformist attitudes [60], reflecting a behavioral misalignment that extends beyond the family unit.

Regarding dysfunctional socio-cognitive processing [10], there are evident difficulties in anticipating the consequences of one’s own behaviors and in selecting effective strategies for problem-solving [26]. However, the extent to which these deficits are assessed varies across studies, limiting the comparability of findings. This reinforces the idea that CPV is associated with cognitive and emotional challenges that compromise social adaptation and self-regulation.

The profile of these adolescents is characterized by a multiplicity of symptoms, ranging from anxiety and mood conditions to neurodevelopmental and behavioral disorders, often accompanied by low self-esteem [21]. Specifically in girls, a higher prevalence of mental health challenges is observed [20], although gender differences are not systematically examined across studies, making it difficult to draw firm conclusions. This indicates that the cumulative effect of poly-victimization and psychological distress exacerbates the psychopathological risk within this group.

Substance use was identified as both a risk and a predictive factor for CPV [40], exacerbating impulsivity and aggression (e.g., [20,21,26]). The fact that assaults often occur while under the influence reinforces the disinhibiting effect of these substances and their influence on impulse control and behavioral regulation [26]. Nonetheless, the directionality of this relationship remains unclear, as some studies do not distinguish between antecedents and consequences of substance use.

Regarding psychopathology, antisocial personality disorder emerged as the most predictive of CPV (e.g., [42,62]), corroborating the prevalence of psychopathic traits reported in the literature. From a developmental perspective, early victimization may compromise interpersonal trust, inducing a hostile and suspicious worldview that fosters the emergence of conflict.

The prominence of depressive and anxious symptomatology [34,35,40] suggests a state of hypervigilance and high stress levels among these adolescents, although their role as risk versus consequence of CPV remains insufficiently clarified in the literature.

Finally, while conduct disorders were identified as being at the core of CPV [45], other clinically relevant conditions were observed with a less pronounced but significant incidence. These include psychotic symptoms [25,57], post-traumatic stress disorder [49] obsessive-compulsive features [25,35], and suicidal ideation [50], highlighting the clinical heterogeneity of this population and the need for more integrative models of explanation.

One important consideration in the interpretation of the present findings is the heterogeneity of the included samples, which comprised judicial, clinical, and community populations. Although this broader inclusion strategy may enhance the external validity and exploratory scope of the review, differences in sample origin may have influenced the observed results. In particular, clinical samples are more likely to present higher levels of psychopathological traits and symptom severity when compared with community-based populations, while judicial samples may reflect more severe behavioral manifestations and contextual risk factors. Consequently, the variability across sample types may limit the direct comparability of findings between studies and should be taken into account when interpreting overall patterns. Therefore, the results should be interpreted with caution, and their generalizability to specific populations may be limited. Future research would benefit from more homogeneous sampling strategies or subgroup analyses to better clarify the influence of sample origin on the observed outcomes.

## 5. Limitations

The most frequently reported limitation in the reviewed literature relates to the small sample sizes and limited representativeness, often consisting of Spanish adolescents, which restricts the cross-cultural generalizability of the findings. Another limitation concerns the exclusive use of self-report measures, which are susceptible to social desirability bias. Furthermore, data collection based solely on parental reports or the analysis of public service records is identified as a methodological constraint. Regarding the assessment of the adolescents’ personality and psychopathology, there is a notable lack of formal clinical diagnoses, lending a speculative nature to some results. Finally, the predominance of cross-sectional designs prevents the establishment of causal inferences between the analyzed variables.

The present systematic review.has limitations that must be considered. Language restrictions and the exclusion of grey literature may have limited access to pertinent information for the study. Despite the emerging interest in this topic, CPV remains an under-researched construct internationally. This scarcity of scientific studies results in difficulties in consolidating evidence regarding the predictors of aggressive behavior toward parents. The sample shows a strong predominance of studies from Spain, with very limited representation from other geographical contexts (only 2 from the USA, 2 from Australia, 2 from Mexico and 1 each from France, South Korea and Japan), which restricts the generalizability of the results and may over-represent specific cultural and contextual factors of this country, compromising its applicability in different sociocultural contexts. Despite claiming to synthesize empirical evidence and describing associations as strong or consistent, this review is exclusively narrative. The absence of any quantitative component (such as meta-analysis or systematization of effect sizes) limits the robustness of these conclusions, preventing the assessment of consistency and the quantification of the magnitude of the reported associations.

## 6. Conclusions

This review focuses specifically on adolescent perpetration of CPV, allowing for a more homogeneous synthesis of predictors. It organizes the evidence from an ecological perspective, clarifying inconsistent results between studies and identifying more robust predictors. Additionally, it highlights relevant gaps in the literature, including the need for longitudinal studies, greater conceptual standardization, and differentiated analysis by type of violence, literature for the development of future research, and more targeted interventions.

The included studies revealed a clear association between CPV and developmental victimization. Victimization and dysfunctional family dynamics during childhood promote the adoption of aggressive responses and the justification of violence. Additionally, the results emphasize that direct or vicarious violence from a parent toward their children is associated with future CPV directed at that same parent. This suggests the social learning of aggressive behaviors and the presence of feelings of resentment, anger, and revenge. The impact of exposure to childhood victimization is manifested in socio-cognitive and emotional developmental challenges, which are associated with a high prevalence of psychopathic traits characterized by poor social interaction and emotional imbalance.

Studies confirm the association between CPV and compromised mental health, with substance use being the risk behavior with the highest incidence in this population. The most prevalent clinical diagnosis associated with CPV was antisocial personality disorder, which validates the high prevalence of psychopathic traits. A range of comorbidities was also identified, encompassing anxious and depressive symptomatology, stress, and ADHD features, which heightens the likelihood of substance use, suicidal ideation, alexithymia, paranoid ideation, and impulsivity.

In summary, the results indicate that CPV is a multifactorial construct, primarily predicted by offenders’ early and continuous exposure to family victimization. This victimization trajectory appears to compromise personality structuring, influencing the development of dysfunctional traits and, consequently, complex psychopathological clinical profiles.

## 7. Implications for Practice and Future Research

In conclusion, integrating these findings into explanatory models is essential for designing preventive and therapeutic strategies tailored to the specific characteristics of these youth. It is crucial to foster both theoretical and applied research to structure multidisciplinary interventions that provide a comprehensive response to the identified needs. Future research should prioritize the development of longitudinal studies to better understand the developmental trajectories of CPV, as well as incorporate qualitative methods to gain a deeper understanding of the subjective experiences of the youth involved and to develop, test, and evaluate the efficacy of interventions.

Against this backdrop, by synthesizing evidence on developmental victimization, personality traits, and psychopathological factors, this systematic review advances a more integrative understanding of child-to-parent violence, offering a theoretically grounded framework to guide future research and the development of targeted preventive and therapeutic strategies.

## Figures and Tables

**Table 1:** Characterization of included studies (*N* = 46).

Author(s) (Year)	Country	Type of Study			Sample Size			Data Analysis		Variables			MMAT Results
		Qua	Quan	Mix	Sm	Med	Large	Descriptive analysis	Inferential Analysis	Victimization	Personality	Psychopathology	
**Armstrong et al. (2018)** [20]	USA		✓				✓	✓	✓	✓	✓		100%
**Bautista-Aranda et al. (2023)** [9]	Spain		✓				✓	✓	✓	✓			80%
**Calvete et al. (2011)** [21]	Spain		✓				✓	✓	✓	✓	✓	✓	80%
**Cano-Lozano et al. (2024)** [22]	Spain		✓				✓		✓	✓			60%
**Cano-Lozano et al. (2021)** [23]	Spain		✓				✓	✓	✓	✓			80%
**Carrasco et al. (2018)** [24]	Spain		✓		✓			✓	✓	✓			60%
**Contreras & Cano (2016)** [25]	Spain		✓		✓				✓		✓		60%
**Contreras & Cano (2015)** [26]	Spain		✓		✓			✓	✓		✓	✓	60%
**Contreras et al. (2020)** [10]	Spain		✓				✓		✓	✓			100%
**Cortina & Martín (2020)** [27]	Spain		✓			✓		✓	✓	✓		✓	80%
**Cuervo (2023)** [28]	Spain		✓			✓		✓	✓		✓		60%
**Cuervo (2025)** [29]	Spain		✓			✓			✓		✓	✓	60%
**Cuervo & Palanques (2022)** [30]	Spain		✓			✓		✓	✓	✓	✓	✓	60%
**Hoyo-Bilbao et al. (2021)** [31]	Spain		✓			✓		✓	✓		✓	✓	80%
**Espuig et al. (2025)** [32]	Spain		✓			✓		✓	✓	✓	✓	✓	80%
**Fandiño et al. (2024)** [33]	Spain		✓			✓			✓		✓	✓	80%
**Fandiño et al. (2021)** [34]	Spain		✓		✓			✓			✓	✓	80%
**Fongaro et al. (2023)** [35]	Fr		✓		✓			✓			✓	✓	80%
**Gámez-Guadix & Calvete (2012)** [36]	Spain		✓				✓	✓	✓	✓			100%
**Harries et al. (2023)** [37]	Aust		✓				✓	✓	✓	✓			100%
**Ibabe (2014)** [38]	Spain		✓			✓		✓	✓	✓	✓		80%
**Ibabe et al. (2013)** [39]	Spain		✓				✓	✓	✓	✓			80%
**Ibabe et al. (2014)** [40]	Spain		✓			✓			✓		✓	✓	100%
**Ibabe & Jaureguizar (2010)** [41]	Spain		✓			✓		✓	✓		✓		100%
**Izaguirre & Calvete (2017)** [42]	Spain		✓				✓	✓	✓	✓			80%
**Jiménez-Granado et al. (2023)** [43]	Spain		✓				✓	✓	✓	✓	✓	✓	80%
**Junco-Guerrero et al. (2022)** [44]	Spain		✓				✓	✓	✓	✓			100%
**Kennedy et al. (2010)** [45]	USA		✓			✓		✓	✓	✓	✓		60%
**Loinaz et al. (2020)** [46]	Spain		✓		✓			✓		✓	✓		60%
**Loinaz et al. (2023)** [47]	Spain		✓			✓		✓			✓		80%
**Maranon & Ibabe (2024)** [48]	Spain		✓			✓		✓	✓	✓	✓		100%
**Martín et al. (2022)** [49]	Spain		✓			✓		✓	✓		✓		80%
**Martínez-Ferrer et al. (2020)** [50]	Mex		✓				✓	✓	✓		✓	✓	80%
**Nam et al. (2022)** [51]	Kor		✓				✓	✓	✓	✓	✓		80%
**Navas-Martínez & Cano-Lozano (2022)** [52]	Spain		✓				✓	✓	✓		✓		60%
**Navas-Martínez & Cano-Lozano (2023)** [53]	Spain		✓				✓	✓	✓	✓	✓		80%
**Navas-Martínez et al. (2023)** [54]	Spain		✓				✓	✓	✓	✓	✓		80%
**Padilla-Falcón & Moreno-Manso (2019)** [55]	Spain		✓			✓		✓			✓	✓	100%
**Palanques et al. (2022)** [56]	Spain		✓			✓		✓	✓	✓	✓		80%
**Rosado et al. (2017)** [57]	Spain		✓				✓	✓	✓			✓	100%
**Sasaki et al. (2021)** [58]	Jap		✓				✓	✓	✓			✓	60%
**Sheed et al. (2024)** [59]	Aust		✓				✓	✓	✓		✓		80%
**Suárez-Relinque et al. (2020)** [60]	Mex			✓			✓	✓	✓		✓		60%
**Suárez-Relinque et al. (2023)** [61]	Esp		✓				✓	✓	✓		✓		60%
**Zuñeda et al. (2016)** [62]	Spain		✓		✓			✓	✓		✓		100%

Note. ✓ = Corresponds to the category shown in the column. Qua = Qualitative (*n* = 0); Quan = Quantitative (*n* = 45); Mix = Mixed methods (*n* = 1); Sm = Small (*n* = 7); Med = Medium (*n* = 17); Large (*n* = 22); USA = United States of America; Sp = Spain; Fr = France; Aust = Australia; Mex = Mexico; Kor = South Korea; Jap = Japan.

## Data Availability

The data presented in this study are available on request from the corresponding author; the data are not publicly available due to privacy.

## Supplementary Materials

The following supporting information can be downloaded at: https://www.mdpi.com/article/10.3390/children13060807/s1, Table S1: This is a table caption.

## Abbreviations

The following abbreviations are used in this manuscript:

CPV	Child-to-Parent Violence
PRISMA	Preferred Reporting Items for Systematic Reviews and Meta-Analyses
MMAT	Mixed Method Appraisal Tool
Qua	Qualitative
Quan	Quantitative
Mix	Mixed methods
Sm	Small
Med	Medium
USA	United States of America
Sp	Spain
Fr	France
Aust	Australia
Mex	Mexico
Kor	South Koreaa
Jap	Japan
ADHD	Attention-Deficit/Hyperactivity Disorder
