# Supplementary material for: Predictors of Child-to-Parent Violence in Adolescence: A Systematic Review
Source: Children (Basel). 2026 Jun 11;13(6):807. doi: 10.3390/children13060807 (PMC13297394; doi:10.3390/children13060807)
Supplement: Supplementary file 1 [file children-13-00807-s001.zip › children-4315138-supplementary.pdf]

Table S1. This is a table caption.

Authors/ publication year	Objetives	Data collection method	Analysis plan	Principal results	Main conclusions and limitations
**Armstrong et al. (2018) [20]**	- To understand the risk profile of youth incarcerated for family and partner violence (CPV);<br>- To analyze sex and ethnicity, and whether they have experienced victimization or intergenerational transmission of violence;<br>- To examine whether CPV indicators vary according to sex.	Data from the 2003 Survey of Youth in Residential Placement (SYRP) [57], made available by the Inter-University Consortium for Political and Social Research (ICPSR).<br>**Sociodemographic characteristics**: questionnaire constructed with sex (male or female); age; ethnicity (non-Hispanic White, non-Hispanic Black, Hispanic, Native American/Asian/Hawaiian, or other/multiple ethnicities); offense severity (murder, weapon offense, or non-weapon offense); and relationship to the victim (biological father, stepfather, adoptive father, or grandfather).<br>**Substance use:** questionnaire constructed with frequency (every day/several times a week, once a week, once a month, or never); simultaneous use of alcohol and drugs; whether drug	**Descriptive analysis:**<br>- Univariate statistics to describe the risk profiles of youth incarcerated for family and partner violence (CPV).<br>**Inferential analysis:**<br>- T-tests to determine mean differences between sexes in the risk profiles of youth incarcerated for CPV.	**Sociodemographic characteristics:** The majority of youth incarcerated for family and partner violence (CPV), of both sexes, were Caucasian (60.1%). Significant sex differences were observed for age (i.e., boys were on average one year older than girls: 16.4 vs. 15.2 years) and ethnicity (i.e., a higher proportion of boys were Caucasian or Black, whereas 33.9% of girls were Hispanic, Native American/Asian/Hawaiian, or from other groups).<br>**Offense characteristics:** Most youth of both sexes were incarcerated for non-weapon assaults and victimized a parent. Girls committed significantly more non-weapon assaults than boys (71.4% vs. 64.7%). Significantly more boys than girls were incarcerated for homicide (9.1% vs. 3.1%), and boys were more likely to victimize a	Incarceration for family and partner violence (CPV) is more prevalent among Caucasian boys and is often associated with weapon use during the offense.<br>Girls report higher levels of direct and vicarious victimization, poly-victimization, emotional problems, and aggressive behaviors as a form of self-defense.<br>Single-parent family contexts and substance use are common factors among these youth. Girls incarcerated for CPV typically begin life in a two-parent family structure but experience higher rates of victimization and sexual abuse, leading to an increased likelihood of substance use and mental health problems.<br>All these factors contribute to problematic behaviors at home and at school.<br>Limitations**:**<br>- It is noted that there are few available studies on CPV, particularly regarding sex differences, highlighting the need for further research to enable the

	use interfered with fulfilling responsibilities; problems experienced while drinking or using drugs; substance use during the offense; and substance use prior to judicial custody.<br>**Mental health:** items from the Massachusetts Youth Screening Inventory (MAYSI) [58]; presence or absence, at the beginning of detention, of depression, anxiety, anger, hallucinations, or suicidal ideation.<br>**Living situation:** assessment of living situation before detention (living with both parents, with one parent, in foster care, with another relative, with friends, homeless/alone/other); and living situation during upbringing (living with both parents, with one parent, without parents, in foster care).<br>**Victimization:** poly-victimization scale, including direct and secondary victimization (physical abuse, sexual abuse, forced sexual relations, emotional abuse, or	biological parent or stepparent than girls (77% vs. 73.4%; 26.9% vs. 23.3%).<br>**Substance use:** Nearly 80% of the sample reported marijuana use, 76% alcohol use, and 76.6% other drug use. Girls were significantly more likely to consume alcohol (82.2% vs. 72.4%) and other illicit drugs (80.7% vs. 74.2%), report problems while using substances (50.3% vs. 36.2%), and had a significantly higher likelihood of being under the influence of drugs and alcohol at the time of the offense (32.7% vs. 24.1%). Boys had a significantly higher likelihood of being under the influence of alcohol alone (7.9% vs. 1.6%).<br>**Mental health:** Girls were significantly more likely to present depression (67.7% vs. 50.6%), anxiety (29.9% vs. 11.0%), anger (67.8% vs. 55.5%), and suicidal ideation (56.4% vs. 37.8%), whereas boys exhibited significantly higher rates of hallucinations (25.9% vs. 22.9%).<br>**Victimization:** Girls reported higher direct victimization (i.e.,	development of effective interventions.

witnessing severe violence).

School problems: low academic performance; diagnosis of learning disorders; and behavioral problems, including school suspension.

Juvenile justice involvement: number of times in judicial custody; and offense history (no history, history without convictions, or with convictions).

experiencing more physical, sexual, or emotional abuse, or forced sexual relations during childhood) and poly-victimization than boys.

Living situation: At the time of the offense, girls were significantly more likely to live in single-parent families (51.2% vs. 44.6%), while boys were more likely to live in two-parent families (38.3% vs. 32.1%). However, during upbringing, the pattern was reversed (i.e., girls were more likely to have lived with both parents, and boys were more likely to have lived in single-parent families). Over the course of development, more girls reported living without parents (13.4% vs. 6.0%) or in foster/group homes (24.2% vs. 18.2%).

Other behavioral problems: The majority of youth incarcerated for CPV, of both sexes, reported school problems, especially previous suspensions (60.5% and 60.3%), as well as prior involvement with the juvenile justice system, including incarceration (90.7% and 80.7%).

**Bautista-Aranda et al. (2025) [9]**	- To analyze the relationship between exposure to family violence before the age of 10 and family and partner violence (CPV);<br>- To examine the relationship between moral disengagement and CPV;<br>- To analyze the mediating role of moral disengagement in the relationship between childhood exposure to family violence and CPV.	**CPV:** Child-to-Parent Violence Questionnaire, adolescent version (CPV-Q-A). **Early exposure to family violence:** Exposure to Violence Scale (VES) [59], Exposure to Violence at Home subscale. **Moral disengagement:** Mechanisms of Moral Disengagement Scale, Spanish version (MMDS-S) [60].	**Descriptive analysis:**<br>- Frequency analyses, including means, standard deviations, skewness, and kurtosis.<br>**Inferential analysis:**<br>- Structural equation modeling (SEM) to examine whether exposure to family violence increases CPV through moral disengagement.	Both father-directed CPV and mother-directed CPV showed positive and significant associations with vicarious and direct exposure to family violence, as well as with moral disengagement. Significant positive direct effects were found between vicarious exposure to family violence and CPV (father-directed CPV, $\beta = 0.248$, SE = 0.064, $p < 0.001$; mother-directed CPV, $\beta = 0.245$, SE = 0.060, $p < 0.001$).<br>Significant positive direct effects were also observed between direct exposure to family violence and CPV (father-directed CPV, $\beta = 0.301$, SE = 0.045, $p < 0.001$; mother-directed CPV, $\beta = 0.315$, SE = 0.042, $p < 0.001$). The indirect effects of both vicarious and direct exposure to family violence on CPV through moral disengagement were positive and significant (vicarious exposure: father-directed CPV, $\beta = 0.037$, SE = 0.014, $p < 0.001$; mother-directed CPV, $\beta = 0.039$, SE = 0.015, $p < 0.05$; direct exposure: father-directed CPV, $\beta = 0.042$, SE = 0.009, $p < 0.001$; mother-directed CPV, $\beta =$	Vicarious and direct exposure to violence in the home, as well as moral disengagement, contribute to explaining family and partner violence (CPV).<br>Family victimization is significantly associated with and predicts CPV.<br>Moral disengagement was found to mediate the relationship between direct and vicarious exposure to family violence and CPV.<br>**Limitations:**<br>- This is a cross-sectional and retrospective study, which does not allow the assessment of long-term effects;<br>- The sample consists of adolescents from four regions of Spain, which limits the generalizability of the findings, and the results are based on self-reports.

				0.045, SE = 0.021, $p < 0.001$). The models were controlled for sex. Results indicated that males scored higher on moral disengagement, whereas females scored higher on both vicarious and direct exposure to violence.	
**Calvete et al. (2011) [21]**	- To assess the prevalence of physical and verbal family and partner violence (CPV) perpetrated by adolescents;<br>- To identify their profiles according to family, educational, community, and personal characteristics.	**CPV:** Conflict Tactics Scales – Child-Parent (CTS-CP) [61].<br>**Beliefs associated with the use of violence:** Justification of Violence subscale of the Irrational Beliefs Scale for Adolescents [61].<br>**Exposure to violence:** Family Violence Exposure subscale of the Exposure to Violence Scale [63].<br>**Social support:** Friends' Support subscale of the Multidimensional Scale of Perceived Social Support [64].<br>**Parental discipline behaviors:** Punitive Discipline, Monitoring, and Inductive Discipline scales from the Dimensions of Discipline Inventory – Form *C* [65].<br>**Aggressive and delinquent behaviors in the peer group:** Questionnaire by Barnow, Lucht & Freyberger (2005).<br>**Depressive symptoms and delinquent behavior:** Affective Problems and Delinquent Behavior and Rule-Breaking subscales of the	**Descriptive analysis:**<br>- Frequency analysis of violent behaviors toward parents.<br>**Inferential analysis:**<br>- Repeated-measures ANOVA to assess whether there were differences in CPV according to the sex of the parent and the adolescent;<br>- Correlation analyses between CPV and environmental and individual variables;<br>- Hierarchical	A high correlation was observed between the Maternal Aggression and Father-Directed Aggression scales (coefficients ranging from 0.60 to 0.77).<br>A four-profile solution was retained, with mean posterior probabilities of Profile 1 = 0.95, Profile 2 = 0.99, Profile 3 = 0.95, and Profile 4 = 1.00. Effect sizes for profile differences were large for all variables ($\eta p^2 > 0.14$), except for parental warmth and lack of limits.<br>Profile 1 (n = 1,354; 82.2%) comprised youth with very low scores on CPV and exposure to family violence. Profile 2 (n = 102; 6.2%) showed moderate levels of psychological CPV and very high levels of both forms of exposure to family violence. Profile 3 (n = 160; 9.7%) was characterized by high levels of psychological CPV and	Verbal CPV was more frequent than physical CPV, with boys tending to perpetrate the latter more often. Mothers experienced more verbal CPV than fathers, with no differences observed in physical aggression toward mothers and fathers. Father-directed CPV was associated with lower scores across several forms of parental discipline, suggesting that these parents apply less punitive discipline. A negative relationship was found between CPV and parental monitoring, indicating a general profile of low parental discipline.<br>CPV was present across all socioeconomic strata. These adolescents showed greater exposure to family violence and stronger associations with peers

	Youth Self-Report (YSR) [68].<br>**Substance use:** Adolescent Drug Use Inventory [66].<br>**Self-esteem:** Rosenberg Self-Esteem Scale (1965).<br>**Impulsivity:** Social Problem-Solving Inventory – Revised, Short Form [67].	multiple linear regression to examine the extent to which each variable contributed to explaining variance in CPV.	very low exposure to family violence. Profile 4 (n = 31; 1.9%) included high levels of CPV, including physical aggression, and high exposure to family violence.<br>Regarding sex, profile distributions were similar, except for Profile 3, in which girls were overrepresented (12.4% girls; 6.9% boys).<br>No association was found between profile membership and socioeconomic status ($\chi^2(12) = 8.44$, $p = 0.750$).<br>Large effect sizes (> 0.14) were observed for aggressive response selection, and moderate effect sizes were found for hostile attribution, anticipation of positive consequences of aggression, and externalizing problems.<br>Profiles 1, 2, and 4 were compared with Profile 3. Profile 3 was associated with high levels of justification of violence and aggressive behavior selection in conflicts with parents. Compared with Profile 4, Profile 3 showed a lower tendency toward aggressive	exhibiting behavioral problems. CPV was positively correlated with substance use, an impulsive style of social conflict resolution, depressive symptoms, and low self-esteem. CPV showed a weak association with the justification of violence, whereas grandiosity was associated with all types of CPV.<br>**Limitations:**<br>- The study relied on adolescentes self-reports;<br>- The scales used were the most commonly applied at the time of the study and did not introduce new assessment methods for this phenomenon;<br>- The study should have assessed the dimension of family affection to better understand children's psychological adjustment.

				responses and positive expectations, but higher levels of anger and depressive symptoms. Compared with Profile 2, Profile 3 was associated with older age, a higher likelihood of including girls, and lower levels of hostility, knowledge structures, anger, and parent–child conflict. Relative to Profile 1, Profile 3 was associated with higher scores in hostility, aggressive behavior selection, depression, and externalizing problems.	
**Cano-Lozano et al. (2024) [22]**	- To clarify the relationship between direct and vicarious exposure to family violence before age 10 and in the past year, and family and partner violence (CPV);<br>- To identify types of family victimization that predict CPV;<br>- To analyze which types of motives for CPV (i.e., reactive, instrumental, or both) mediate the	**CPV:** Child-to-Parent Violence Questionnaire, adolescent version (CPV-Q) [68].<br>**Exposure to family violence at home:** Violence Exposure Scale, adapted version (VES) [69], Exposure to Violence at Home subscale with adaptation to include two different time points (i.e., during childhood – before age 10, and during the past year).	**Inferential analysis:**<br>- Correlation analyses to examine relationships between the study variables;<br>- Stepwise multiple linear regression, conducted separately for boys and girls, to evaluate the extent to which each variable	CPV was positively related to both direct and vicarious exposure to family violence at home during the past year and childhood, for both boys and girls. Correlation coefficients were higher for direct victimization (r = .27 to r = .40) than for vicarious victimization (r = .20 to r = .30), as well as for exposure during the past year compared to childhood. In boys, CPV toward parents correlated more strongly with victimization perpetrated by the father (r = .40) than by the mother (r = .32). In girls, CPV toward mothers correlated more strongly	A positive relationship was found between CPV and both direct and vicarious exposure to family violence during the past year and childhood, in both sexes.<br>Stronger correlations were observed for direct victimization occurring in the past year, suggesting that immediate effects are more intense. CPV correlated strongly with father-perpetrated victimization in boys, while violence toward mothers correlated more with mother-perpetrated victimization in girls.

	relationship between different types of family victimization and whether these effects differ by sex.			contributed to explaining the variance in CPV toward the mother and toward the father;<br>- Mediation analyses, conducted separately for boys and girls, to assess whether motives for CPV influence the relationship between exposure to family violence at home and CPV.	with victimization perpetrated by the mother (r = .39) than by the father (r = .33).<br>Both types of victimization showed higher correlations with reactive motives (r = .24 to r = .55) than with instrumental motives (r = .24 to r = .29). The only non-significant correlation between motives for CPV and exposure to family violence was found in girls: instrumental motives for CPV did not significantly correlate with vicarious victimization (father to mother) during childhood.<br>Both types of exposure to family violence showed higher correlation coefficients with reactive motives (r = 0.24 to r = 0.55) than with instrumental motives (r = 0.08 to r = 0.29). Significant positive correlations were also found between reactive and instrumental motives and CPV toward both father and mother in boys and girls.<br>Both direct and vicarious victimization significantly predicted CPV in both sexes.<br>**CPV toward father:**	Vicarious victimization predicted CPV when occurring during childhood, indicating more compensatory effects associated with this type of victimization.<br>In boys, CPV was motivated by both reactive and instrumental reasons across all types of victimization, except for vicarious victimization of father-to-mother violence during the past year, which was related to reactive reasons.<br>**Limitations:**<br>- Retrospective longitudinal design;<br>- Although large, the sample was not fully representative as it included only Spanish youth aged 13 to 17 years;<br>- The measures used were self-reports.

The proportion of variance explained was slightly higher in boys ($R^2 = .201$ vs. $R^2 = .148$). Direct victimization by the father was the strongest predictor in both boys and girls, with sex differences observed in vicarious victimization. In boys, CPV toward the father was explained by vicarious exposure to father-to-mother violence during the past year. In girls, CPV toward the father was explained by vicarious exposure to mother-to-father violence during the past year.

CPV toward mother: The proportion of variance explained was also slightly higher in boys ($R^2 = .239$ vs. $R^2 = .194$). Direct victimization by the mother during the past year and by the father during childhood were common predictors in both boys and girls, with sex differences found in vicarious victimization. In boys, CPV toward the mother was explained by vicarious exposure to father-to-mother violence during childhood. In girls, CPV toward the mother was explained by vicarious exposure

				to mother-to-father violence during childhood. **Influence of motivations for CPV:** In boys, the relationship between violence toward the father and direct victimization by him (during the past year and childhood) was influenced by both instrumental and reactive motives. However, the relationship between violence toward the father and vicarious victimization of father-to-mother violence (during the past year) was more influenced by reactive motives. In girls, the relationships between CPV and all victimization experiences were more influenced by reactive motives than instrumental motives, with one exception: the relationship between violence toward the mother and direct victimization by the father during childhood was equally influenced by reactive and instrumental motives.	
**Cano-Lozano et al. (2021) [23]**	- To examine the frequency of family	Measures were administered exclusively during the COVID-19	**Descriptive analysis:**	**Type of CPV:** Overall, 65.2% of adolescents reported having	More than half of the adolescents engaged in at least one

	and partner violence (CPV) in the Spanish youth population toward both parents, as well as other forms of family violence, and their relationship with different psychosocial stressors;<br>- To understand sex differences;<br>- To determine the individual and additive contributions of different types of family violence and different types of psychosocial stressors in predicting CPV.	lockdown period, from March 14 to May 10, 2020.<br>**CPV:** Child-to-Parent Violence Questionnaire, youth version (CPV-Q-J) [19].<br>**Sociodemographic data:** participant characteristics (sex, age, nationality, and educational level) and family structure (parents' marital status and educational level, and number of siblings).<br>**Family violence:** Violence Exposure Scale (VES), Domestic Violence subscale [63].<br>**Psychosocial stressors:** a questionnaire was developed to assess psychosocial stressors related to lockdown (academic/work-related stressors, family coexistence, financial stressors, COVID-19-related stressors, physical health, and mental health).	**-** Frequency analyses of CPV, other types of family violence, and psychosocial stressors related to lockdown;<br>- Frequency of accumulated stressors.<br>**Inferential analysis:**<br>- Chi-square analyses to examine sex differences in both types of violence and psychosocial stressors;<br>- Spearman's bivariate correlations to analyze relationships between CPV toward mothers and fathers and other types of family violence and psychosocial stressors;	engaged in some form of violent behavior against their mother, and 59.4% against their father. A significantly higher proportion of girls, compared to boys, reported perpetrating CPV against mothers [$\chi^2(1, 2221) = 22.5$, $p < .001$] and against fathers [$\chi^2(1, 2181) = 10.4$, $p = .001$].<br>The most frequent type of CPV was psychological violence (40.1% to 61.3%), followed by control/domination behaviors (36.5% to 43%), economic violence (12% to 16.6%), and physical violence (1.7% to 3.3%).<br>Compared to boys, girls perpetrated significantly more psychological CPV against both mothers and fathers [$\chi^2(1, 2221) = 33.1$, $p < .001$; $\chi^2(1, 2181) = 28.9$, $p < .001$, respectively], and reported more control/domination behaviors against mothers [$\chi^2(1, 2221) = 4.0$, $p = .040$].<br>In contrast, boys perpetrated significantly more physical and economic violence against fathers compared to girls [$\chi^2(1, 2181) = 6.0$, $p = .014$; $\chi^2(1, 2181) = 8.2$, $p =$	aggressive behavior toward their parents within a one-month period, with mothers being the most affected. Girls perpetrated more psychological CPV and control/domination behaviors against mothers, whereas boys engaged more frequently in physical and economic violence.<br>Sixteen percent of adolescents reported experiencing some form of violence perpetrated by their mother, and 14.9% by their father. Girls reported higher levels of psychological violence perpetrated by mothers.<br>The majority of adolescents reported high concern regarding school/work, the virus, and mental health, and more than 90% experienced an accumulation of psychosocial stressors.<br>Positive associations were found between CPV and parent–child violence, as well as between CPV and exposure to interparental violence. The strongest correlations were observed between CPV against mothers and mother-to-child violence,

	- Hierarchical multiple linear regressions to determine the individual and additive effects of types of family violence and stressors	.004, respectively].<br>**Exposure to Parental Violence:** Sixteen percent of adolescents reported experiencing at least one violent behavior perpetrated by the mother, and 14.9% by the father. The most frequent type of parental violence was psychological violence (12.4%–17.1%), followed by verbal violence (4.6%–5.7%) and physical violence (2.8%–3.4%).<br>Compared to boys, significantly more girls were victims of psychological violence perpetrated by their mothers [$\chi^2(1, 2221) = 9.7$, $p = .002$], and witnessed more psychological violence from father to mother [$\chi^2(1, 2159) = 6.5$, $p = .010$].<br>Regarding vicarious exposure, 11.6% of adolescents witnessed violence from the mother against the father, and 11.9% witnessed violence from the father against the mother. Exposure to psychological violence between parents was the most frequent (9.9%–13.4%), followed by verbal violence (0.4%–1.2%) and physical violence (0.9%–1%).	and between CPV against fathers and father-to-child violence. CPV against both parents was significantly associated with psychosocial stressors and their accumulation. Academic/work-related stressors, family cohabitation stressors, and mental health stressors predicted CPV against both parents. Family cohabitation stressors were the strongest predictors of CPV against mothers, whereas mental health stressors were the strongest predictors of CPV against fathers.<br>Overall, family violence and psychosocial stressors acted as significant predictors and, jointly, explained a greater proportion of variance in CPV.<br>**Limitations**<br>- The study employed a cross-sectional design, which does not allow for the identification of causal relationships;<br>- The sample showed an overrepresentation of university students and an underrepresentation of adolescents attending primary

Frequency of Psychosocial Stressors Related to Lockdown: Regarding psychosocial stressors during confinement, 44.6% of adolescents experienced an academic/work-related stressor, which was significantly more frequent among girls [$\chi^2(1, 2245) = 19.7$, $p < .001$]. The most frequently reported stressors were fear of academic outcomes (95.9%) and fear regarding job continuity (88.9%).

Additionally, 29.3% experienced a family cohabitation-related stressor (21.8% reported lack of personal space and 12.6% difficulties spending time with family), which was also more frequent among girls [$\chi^2(1, 2245) = 10.5$, $p = .001$].

Both sexes experienced economic and physical health stressors at similar rates. COVID-19–related stressors were reported by 45.1% of adolescents, with infections being the most frequent (43.4%), and a significantly higher proportion of girls reporting this stressor [$\chi^2(1, 2245) = 32.5$, $p < .001$].

and secondary education;

- Few items were used to assess each psychosocial stressor, limiting the depth of their measurement.

A very high proportion (96.6%) reported mental health stressors, again more frequent among girls [χ^2(1, 2245) = 31.8, $p < .001$]. Specifically, 88.4% reported psychological impact on a family member, 87.1% psychological impact on themselves, and 77% sleep disturbances.

Accumulation of Psychosocial Stressors: An accumulation of psychosocial stressors was observed in 94.7% of adolescents, with girls experiencing a significantly higher proportion of cumulative stressors compared to boys [χ^2(1, 2245) = 18.6, $p < .001$].

Relationship Between CPV, Family Violence, and Psychosocial Stressors: CPV against both mothers and fathers showed positive and significant correlations with all types of family violence included in the study. The highest correlation coefficients were found between CPV against mothers and mother–child violence [r(2159) = .37, $p < .001$], and between CPV against fathers and

father–child violence [r(2159) = .37, $p < .001$].

CPV was also positively and significantly correlated with both types of psychosocial stressors and their accumulation (CPV against mother: r(2245) = .16, $p < .001$; CPV against father: r(2245) = .15, $p < .001$).

Multiple Linear Regression Analyses Predicting CPV: Regarding CPV against mothers, 15.8% of the variance was explained by mother–child violence (β = .392, $p < .001$, $R^2 = .158$). The explained variance increased with exposure to mother-to-father violence (β = .120, $p < .001$, $R^2 = .168$), as well as academic/work stressores (β = .065, $p = .001$), family cohabitation stressores (β = .093, $p < .001$), and mental health stressores (β = .057, $p = .005$). The final model [$F(7, 2158) = 70.91$, $p < .001$, $R^2 = .187$] indicated that the additive effect of different types of family violence and specific psychosocial stressors explained 18.7% of CPV against mothers.

For CPV against fathers, 17.5% of

				the variance was explained by father–child violence (β = .383, $p < .001$, $R^2 = .175$), increasing with exposure to mother-to-father violence β = .139, $p < .001$, $R^2 = .188$), and academic/work, family cohabitation, and mental health stressors (β = .053, $p = .007$; β = .077, $p < .001$; β = .086, $p = .025$, respectively). The final model [$F(7, 2158) = 77.44$, $p < .001$, $R^2 = .201$] showed that the additive effect of family violence and psychosocial stressors explained 20.1% of CPV against fathers. **Effect of Cumulative Psychosocial Stressors:** When including the accumulation of psychosocial stressors, cumulative stress had a significant effect on the prediction of CPV against both mothers (β = .107, $p < .001$) and fathers (β = .095, $p < .001$), compared to the effect of each stressor considered separately.	
**Carrasco et al. (2018) [24]**	- To examine differences in risk	All cases recorded by the Municipal Social Emergency	**Descriptive analysis:**	54.2% (n = 39) of the youths were classified in Group 1, while 45.8%	Statistically significant differences were found in family separation

	factors among adolescents and young people who have perpetrated CPV, according to the type of family structure to which they belong;<br>- To identify the primary victim of violent behavior as a function of family composition.	Service (Servicio Municipal de Urgencias Sociales, SMUS) of Bilbao (Basque Autonomous Community, Spain) classified as domestic violence or domestic violence perpetrated by minors/young people (up to 21 years of age) were analyzed, covering the period from January 2008 to June 2013.	- Frequency analysis of variables in the two groups (Group 1: apparently normalized families; Group 2: families at social risk).<br>**Inferential analysis:**<br>- Chi-square test for independent samples to estimate differences in frequencies between groups across risk factors for CPV.	(n = 33) were in Group 2. Risk factors were highly prevalent in the total sample, with school failure (90.7%), substance use (87.8%), and psychopathology (86.9%) being the most predominant.<br>Significant differences were found in family separation and exposure to violence, with Group 1 showing a higher frequency in both risk factors compared to Group 2, with moderate to large effect sizes (respectively, r = .57 and r = .54). The sample included 21 nuclear families (29%), 21 reconstituted families (29%), and 30 single-parent families (42%). In 81% of the cases (n = 51), there was correspondence between family type and the primary victim of CPV. The mother was the most frequent sole victim of CPV in single-parent families (70.7%) and in reconstituted or nuclear families (29.3%).<br>In nuclear and reconstituted families, where both parents are present (n = 42; 48.3%), CPV was perpetrated against both parents in 66.7% of these cases (n = 28).	and exposure to violence between both groups.<br>The most predominant risk factors were school failure, substance use, and psychopathology.<br>Group 1 showed a higher frequency of family separation (n = 37; 94.87%) and a higher frequency of exposure to violence (n = 23; 100%).<br>The mother was the primary victim of CPV, which may be due to her being the sole focus in single-parent families. In nuclear and reconstituted families, the mother was also the most frequent victim, but both parents were assaulted.<br>**Limitations**:<br>- Information was obtained through parental reports and a public service, excluding data from private support services and self-reports from youths;<br>- Limited information was gathered regarding the risk factor of psychopathology;<br>- The sample included only heterosexual couples.

**Contreras & Cano (2016) [25]**	-	To analyze emotional intelligence, social attitudes, and personal values of adolescents who have committed a crime of CPV, examining whether there are differences compared to non-offending adolescents, through direct and individual assessments of the participants.	**Emotional Intelligence:** Trait Meta-Mood Scale (TMMS-24), Spanish version [70].<br>**Social Attitudes:** Attitudes and Social Cognitive Strategies Questionnaire (Cuestionario de Actitudes y Estrategias Cognitivas Sociales, AECS) [71].<br>**Personal Values:** Schwartz's Values Questionnaire, Spanish version.	**Inferential Analysis:**<br>- MANOVA with group as the independent variable to assess group differences in social attitudes;<br>- MANOVA with group as the dependent variable to assess group differences in personal values.	Significant main effects of group were found for emotional intelligence, $\lambda = .57$, $F(3, 54) = 13.30$, $p < .001$, $\eta^2 = .42$, and for attention to feelings, $F(1, 56) = 21.78$, $p < .001$, $\eta^2 = .28$, clarity of feelings, $F(1, 56) = 9.31$, $p < .01$, $\eta^2 = .14$, and mood repair, $F(1, 56) = 22.90$, $p < .001$, $\eta^2 = .29$.<br>Significant overall main effects of group were found for social attitudes, $\lambda = 0.59$, $F(9, 48) = 3.64$, $p < 0.01$, $\eta^2 = 0.41$. For prosocial attitudes, significant group effects emerged for social sensitivity, $F(1, 56) = 11.07$, $p < 0.001$, $\eta^2 = 0.16$, helping and cooperation with others, $F(1, 56) = 13.14$, $p < 0.001$, $\eta^2 = 0.19$, and conformity with norms, $F(1, 56) = 17.94$, $p < 0.001$, $\eta^2 = 0.24$.<br>Regarding antisocial and asocial attitudes, significant group effects were found for dominance, $F(1, 56) = 3.89$, $p < 0.05$, $\eta^2 = 0.06$, and aggressiveness/stubbornness, $F(1, 56) = 5.40$, $p < 0.05$, $\eta^2 = 0.08$. Asocial attitudes indicated significant group effects for apathy-reluctance, $F(1, 56) = 6.74$, $p < 0.05$, $\eta^2 = 0.11$.	The results indicated that parental aggressors, compared to non-aggressive adolescents, exhibited lower levels of emotional intelligence, less prosocial attitudes, and more antisocial attitudes, as well as higher hedonism and power values.<br>Regarding emotional intelligence, aggressors showed less attention to feelings, clarity of feelings, and mood repair, reflecting a reduced ability to identify, express, and regulate emotions and feelings. This leads to poorer emotional regulation and socially appropriate behaviors, particularly within the family environment.<br>Concerning social relationships, parental aggressors demonstrated lower social sensitivity, a reduced tendency to help and cooperate with others, and less conformity to social norms. In terms of antisocial attitudes, aggressors showed a greater tendency towards aggressiveness and dominance, indicating a higher

				Significant main effects of group were also observed for personal values, $\lambda = 0.44$, $F(10, 47) = 5.88$, $p < 0.001$, $\eta^2 = 0.56$. Significant group effects were found for hedonism, $F(1, 56) = 4.89$, $p < 0.05$, $\eta^2 = 0.08$, power, $F(1, 56) = 4.85$, $p < 0.05$, $\eta^2 = 0.07$, universalism, $F(1, 56) = 15.63$, $p < 0.001$, $\eta^2 = 0.22$, benevolence, $F(1, 56) = 4.64$, $p < 0.05$, $\eta^2 = 0.07$, security, $F(1, 56) = 4.42$, $p < 0.05$, $\eta^2 = 0.07$, conformity, $F(1, 56) = 22.41$, $p < 0.001$, $\eta^2 = 0.28$, and tradition, $F(1, 56) = 28.33$, $p < 0.001$, $\eta^2 = 0.33$.	likelihood of disrespecting authority figures, engaging in aggressive behaviors, and a propensity to intimidate, threaten, and manipulate.<br>Regarding personal values, aggressors tended to place more importance on hedonism, power, and control. They assigned little importance to conformity and tradition values, such as respect for parents and social norms, whereas non-aggressors scored higher in self-transcendence and conservation values.<br>**Limitations**:<br>- The study was based on cross-sectional data;<br>- Small and non-representative sample.
**Contreras & Cano (2015) [26]**	- To explore the psychological traits of adolescents reported for violence against parents, analyzing how they differ from other delinquent and non-delinquent adolescents in terms of clinical diagnoses and	**Clinical diagnosis and psychological/psychiatric treatment:** Information extracted from offenders' case files, including reports from the judicial psychosocial team and social services.<br>**Substance use:** Assessed through a structured ad hoc interview.<br>**Social-cognitive style:** Measured by the *Questionnaire of Attitudes and Social Cognitive Strategies*	**Descriptive analysis:**<br>- Frequency analyses of variables related to clinical diagnosis, psychological/psychiatric treatment, and substance use.	Regarding clinical diagnosis and psychological/psychiatric treatment, 30% of youths in the CPV group (n = 9) presented some diagnosis, the most common being Attention Deficit Hyperactivity Disorder (ADHD) (55.6%; n = 5), and Adjustment Disorder with Conduct Disorder (n = 2). No clinical diagnosis was found in the non- CPV group.	A higher prevalence of clinical diagnoses was found among youths who committed CPV (30%) compared to offenders in general. The most frequent diagnoses were ADHD and Conduct Disorder.<br>A greater percentage of CPV offenders received psychological (60%) or psychiatric treatment (20%) during the legal process

	psychological/psychiatric treatment;<br>- To examine substance use, social-cognitive style, and self-esteem.	(Cuestionario de Actitudes y Estrategias Cognitivas Sociales - AECS) [71].<br>**Self-esteem:** Measured by the *Rosenberg Self-Esteem Scale* (RSES) [72].	**Inferential analysis:**<br>- Chi-square tests to explore differences between groups with and without CPV (filial parent violence) for the clinical, treatment, and substance use variables;<br>- MANOVA with group as the independent factor to examine group differences in social-cognitive style;<br>- ANOVA with group as the independent factor to explore differences in self-esteem between groups;<br>- Logistic regression to predict group membership (presence vs.	Significant differences between groups were found in psychological treatment [$\chi^2(1, N = 60) = 10.00$, $p < .001$, $\varphi = 0.38$] and psychiatric treatment [$\chi^2(1, N = 60) = 4.04$, $p < .05$, $\varphi = 0.25$], with a higher percentage of youths in the CPV group receiving psychological (60%, n = 18) or psychiatric treatment (20%, n = 6) compared to the non- CPV group (20%, n = 6 and 0%, respectively). Regarding substance use, significant group differences were found for legal substances [$\chi^2(2, N = 90) = 15.41$, $p < .001$, $\varphi = 0.41$], with a significantly higher percentage of youths in both the CPV group (93.3%, n = 28) and the non- CPV group (90%, n = 27) using these substances. For illicit drugs, both offender groups were significantly more likely to use hashish [$\chi^2(2, N = 90) = 42.02$, $p < .001$, $\varphi = 0.68$], as well as cocaine [$\chi^2(2, N = 90) = 10.96$, $p < .05$, $\varphi = 0.35$]. Regarding socio-cognitive style, significant main effects of group were found [$\lambda = .58$, $F(20, 150) = 2.28$, $p < .001$, $\eta^2 = .23$], but no sex effects [$\lambda = .93$, $F(10, 75) =$	compared to offenders in general (20% and 0%, respectively).<br>Both offender groups reported substance use. Regarding illicit drugs, both offender groups were more likely to consume them, suggesting that these youths have a higher propensity for substance use than the general population. Additionally, 46.7% of youths who committed CPV were violent towards their parents under the influence of drugs or alcohol.<br>Within the CPV group, youths showed lower ability to anticipate the consequences of their behaviors and to select behaviors appropriate to social goals. Both offender groups demonstrated tendencies toward impulsivity and independence, exhibiting difficulties in socio-cognitive strategies and a more negative perception of parental authority as well as parental acceptance and affection. They also showed lower cognitive strategies for resolving interpersonal problems,

	absence of CPV) using all variables as predictors.	.48, $p > .05$] nor Group × Sex interaction [$\lambda = .81$, $F(20, 152) = .79$, $p > .05$]. Specifically, significant group effects were observed for impulsivity/reflexivity [$F(2, 84) = 5.79$, $p < .05$, $\eta^2 = 0.12$], dependence/independence [$F(2, 84) = 5.92$, $p < .05$, $\eta^2 = 0.12$], perception of parental authority [$F(2, 84) = 7.06$, $p < .05$, $\eta^2 = .14$], perception of parental acceptance and affection [$F(2, 84) = 14.53$, $p < .001$, $\eta^2 = 0.26$], ability to perceive and retain relevant information about social situations [$F(2, 84) = 3.82$, $p < .05$, $\eta^2 = 0.08$], ability to anticipate and understand the consequences of social behaviors [$F(2, 84) = 7.25$, $p < .05$, $\eta^2 = 0.15$], and ability to select appropriate means to achieve social behavior goals [$F(2, 84) = 7.86$, $p < .05$, $\eta^2 = 0.16$]. Bonferroni post hoc comparisons indicated that youths in both the CPV and non-CPV groups scored significantly higher on independence and impulsivity and significantly lower on the ability to perceive and retain relevant social information. The CPV group also	anticipating and understanding the consequences of social behaviors, and selecting appropriate means to achieve social objectives.<br>Perception and expectations of social relationships, as well as the capacity to seek solutions for social behaviors, did not differ between offenders and non-offenders.<br>All three groups showed similarities in self-esteem levels; however, males demonstrated higher self-esteem compared to females.<br>**Limitations:**<br>- Study based on cross-sectional data;<br>- Small and non-representative overall sample;<br>- Privacy concerns limited access to many other cases.

showed a significantly more negative perception of both parental authority and parental acceptance and affection, as well as a significantly lower ability to anticipate and understand the consequences of social behaviors and to select appropriate means to achieve goals, compared to other groups.

ANOVA showed no significant group effects for self-esteem [$F(2, 84) = 1.90$, $p > .05$], but significant sex effects [$F(2, 84) = 6.46$, $p < .05$, $\eta^2 = 0.07$], with males scoring significantly higher than females ($M = 31.2$; $SD = 0.76$ vs. $M = 25.13$, $SD = 2.26$). No significant Group × Sex interaction effects were found for self-esteem [$F(2, 84) = 1.87$, $p > .05$]. Regarding predictors of CPV, the full logistic regression model including all predictors was statistically significant [$\chi^2(13, N = 90) = 71.26$, $p < .001$, Nagelkerke $R^2 = 0.76$, with 90% correct classification]. Significant contributors to the model were impulsivity/reflexivity, perception of parental authority, and perception of parental

					acceptance and affection.	
**Contreras et al. (2020) [10]**	-	To investigate the relationship between exposure to domestic violence, socio-cognitive variables, and whether reactive or instrumental use of aggression may help clarify the nature of the type of family violence.	Exposure to domestic violence was assessed using the Exposure to Violence Scale [63], specifically the Exposure to Violence at Home subscale. Child-to-parent violence (CPV) was measured with the Child-to-Parent Violence Questionnaire (CPV-Q) [68]. Social information processing was evaluated through the Social Information Processing (SIP) in Child-to-Parent Conflicts Questionnaire [73]. Justification of violence was assessed using the Justification of Violence Subscale from the Irrational Beliefs Scale for Adolescents [62].	Analyses were conducted using the Lavaan package in R, with graphical modeling of the Structural Equation Models (SEM) performed using Ωnyx software.<br>Confirmatory Factor Analysis (CFA) was employed to assess the fit of each of the subscales used.<br>**Inferential analysis:**<br>- Structural Equation Modeling (SEM).	**Confirmatory Factor Analysis (CFA) of the scales:** All subscales used showed good to excellent reliability indices, and the CFA demonstrated acceptable to excellent model fit indices:<br>**Exposure to Violence**: $\chi^2(5) = 43.6$, $p < 0.001$, CFI = 0.988, RMSEA = 0.069; $\alpha = 0.832$; $\omega = 0.834$<br>**CPV-Q Mother** (CPV–Q M): $\chi^2(68) = 483$, $p < 0.001$, CFI = 0.928, RMSEA = 0.061; $\alpha = 0.792$; $\omega = 0.827$<br>**CPV -Q Father** (CPV–Q F): $\chi^2(67) = 590$, $p < 0.001$, CFI = 0.907, RMSEA = 0.069; $\alpha = 0.788$; $\omega = 0.823$<br>**Reasons Subscale**: $\chi^2(16) = 122$, $p < 0.001$, CFI = 0.958, RMSEA = 0.063; $\alpha = 0.705$; $\omega = 0.717$<br>**Social Information Processing in CPV:** $\chi^2(169) = 1811$, $p < 0.001$, CFI = 0.866, RMSEA = 0.077; $\alpha = 0.785$; $\omega = 0.812$<br>**Justification of Violence**: $\chi^2(22) = 111$, $p < 0.001$, CFI = 0.971, RMSEA = 0.049; $\alpha = 0.761$; $\omega = 0.778$.<br>**Structural Equation Modeling (SEM):** The factor "exposure to violence" was related to hostile	It was understood that exposure to violence promotes the development of maladaptive social-cognitive processing. Maladaptive elements of social-cognitive processing were related to different types of CPV behaviors.<br>Exposure to violence at home was positively associated with hostile attributions, anger (reflecting emotional dysregulation), aggressive responses, anticipation of positive consequences of aggression, and justification of violence.<br>Anger and access to aggressive responses were positively related to CPV against both parents.<br>A strong relationship was found between aggressive responses and CPV motivated by reactive reasons.<br>Anticipation of positive consequences of aggression and justification of violence were positively related to the instrumental use of CPV against

					attribution, anger, aggressive responses, positive consequences, empathy, and justification of violence. Furthermore, while hostile attribution, anger, and aggressive response were related to reactive reasons, positive consequences, empathy, and justification of violence were related to instrumental reasons. The model showed similar fit for both mothers and fathers. All proposed relationships were significant except for the relationship between exposure to violence and hostile attribution, and any relationships involving empathy.<br>Standardized structural regression coefficients between latent variables ranged from 0.842 (exposure to violence – hostile attribution) to -0.013 (exposure to violence – empathy) for mothers, and from 0.840 (exposure to violence – hostile attribution) to -0.012 (exposure to violence – empathy) for fathers.	both parents.<br>**Limitations:**<br>- Study based on cross-sectional data;<br>- Absence of parent reports;<br>- Despite the large sample size, the sample was not representative of other countries, including only youth from two regions in Spain.
**Cortina & Martín (2020) [27]**	-	To analyze different forms of CPV	**CPV**: Question "During your time living with your parents or guardians, how often do you	**Descriptive analysis:**<br>- Frequency	All participants reported having used violence against their parents, ranging from the mildest	Most adolescents committed at least one act, primarily insulting; however, the more severe

	and their relationship with two groups of variables: one comprising sociodemographic variables commonly studied in CPV research, and the other including personality traits and exposure to violence variables.	perform or have you performed any of the following behaviors?"<br>**Exposure to violence**: Observed Violence Scale [63].<br>**Self-concept:** Self-Concept Form-5 Scale (AF5) [74].<br>**Parental affection**: Inventory of Parent and Peer Attachment (IPPA) [75], subscales for mother and father.<br>**Psychopathy**: Inventory of Callous-Unemotional Traits [76].<br>**Narcissism**: Narcissism Scale [77].<br>**Sexism**: Ambivalent Sexism Inventory (ASI) [81].<br>**Social desirability**: Social Desirability Scale (SDS) [82].	analysis of the variables under study.<br>**Inferential analyses:**<br>- Student's t-test (for quantitative variables) and Chi-square test (for categorical variables) to compare youth who engaged in each behavior with those who did not;<br>- Binary logistic regression analysis using stepwise forward method to assess the predictive capacity of each variable among participants who engaged in CPV behaviors.	behavior of insulting (57%) to the most severe behavior of hitting (3.6%).<br>Significantly elevated rates were found for the following acts: Insulting (.573), Z(N = 225) = 35.99, p = .000, δ = 1.83, PSES = .803; Running away (.182), Z(N = 225) = 9.08, p = .000, δ = .74, PSES = .397; Obscene gestures (.142), Z(N = 225) = 6.33, p = .000, δ = .57, PSES = .311; Stealing (.133), Z(N = 225) = 5.71, p = .000, δ = .53, PSES = .289; Destroying property (.093), Z(N = 225) = 2.95, p = .003, δ = 0.32, PSES = .182; and Intimidating (.084), Z(N = 225) = 2.34, p = .019, δ = 0.27, PSES = .151. Insulting occurred in isolation in 83.8% of cases, whereas spitting, intimidating, and hitting never occurred alone but always alongside other CPV acts. A significant association was found between substance use and running away from home [χ2(1) = 6.22, p = .013, OR = 1.57; 24.6% of participants who used substances reported running away] and hitting [χ2(1) = 4.50; p = .034, OR = 1.46; 6.1% of participants who	behaviors never occurred in isolation. Spitting, intimidating, and hitting always co-occurred with other CPV acts.<br>No significant differences were found regarding sex, age, or family structure, but differences emerged in drug use, academic performance, exposure to violence, self-concepts, attachment and communication with the mother, hostile and benevolent sexism, and psychopathic traits (cruelty and indifference).<br>Regarding personality traits, cruelty was associated with obscene gestures, indifference with spitting, and Machiavellianism with incurring debts.<br>**Limitations**:<br>- Unequal group composition;<br>- Low frequency of some behaviors;<br>- Identified social desirability bias.

used substances reported hitting their parents]. The frequency of substance use was also related to running away [$t(42,234) = 2.28$, $p = .028$, $\delta = .52$], with minors who ran away reporting higher consumption frequency ($M = 4.24$, $SD = 2.51$) than those who did not ($M = 3.05$, $SD = 2.19$). A significant relation was also found between running away and the presence of psychopathology [$\chi 2(1) = 4.36$, $p = .038$, $OR = 1.46$; 44.4% of participants diagnosed with mental illness reported having run away]. Additionally, a statistically significant relationship was observed between academic performance and incurring debts [$t(223) = 2.49$, $p = .013$, $\delta = .626$], where participants who incurred debts to their parents had poorer academic performance ($M = 5.47$, $SD = 1.70$) than those who did not ($M = 6.5$, $SD = 1.69$). A significant relationship was also found between education level and intimidating behavior [$\chi 2(3) = 9.83$; $p = .020$, $OR = 1.76$].

The highest mean scores were for

family self-concept (7.81), followed by social self-concept, communication with the mother, trust with the father, and academic self-concept, ranging between 6 and 7.

Predictive variables for the CPV group were: indifference, cruelty, narcissism, Machiavellianism, hostile sexism, benevolent sexism, communication with the mother, anger toward the mother, family self-concept, physical self-concept, witnessing violence at home, and experiencing violence on the street. Experiencing violence on the street increased the odds of running away by 1.26 and of making obscene gestures by 1.34.

Narcissism increased the odds of running away by 1.24, and cruelty (insensitivity) increased the odds of making obscene gestures by 1.45.

Hostile sexism doubled (2.28) the odds of spitting and increased by 1.26 the odds of stealing from parents.

Indifference also doubled (2.78) the odds of spitting.

				Benevolent sexism reduced the odds of spitting by almost half (1.96), and communication with the mother reduced the odds of stealing by 1.25. Physical self-concept reduced the odds of insulting by 1.16, while a high family self-concept increased the odds of incurring debts by 1.31.<br>Finally, Machiavellianism increased the odds of incurring debts by 1.29; witnessing violence at home increased the odds of destroying property by 1.35; and anger toward the mother increased the odds of hitting by 1.48.	
**Cuervo (2023) [28]**	- To analyze the differences between the profiles of youths who committed CPV and those who committed other crimes;<br>- To examine the specific influence of three groups of variables: psychological characteristics of the	Analysis of juvenile records from 2010 to 2017.<br>Assessment of juveniles: Youth Level of Service/Case Management Inventory (YLS/CMI) [78].	**Descriptive analysis:**<br>- Frequency analyses of the variables.<br>**Inferential analyses:**<br>- Chi-square test;<br>- ANOVA to examine significant differences in continuous variables;	Family circumstances showed a strong positive correlation with CPV ($r = .55$, $p < .001$), followed by antisocial personality traits ($r = .43$, $p < .001$), depressive symptoms ($r = .36$, $p < .001$), and exposure to violence ($r = .24$, $p < .001$). Low self-esteem ($r = .24$, $p < .001$), callousness ($r = .15$, $p < .005$), and adoption status ($r = .13$, $p < .005$) were also significantly associated with CPV. Male sex was negatively	The CPV group exhibited higher levels of emotional deficits, callousness, elevated antisocial personality traits, and lower self-esteem. Within the family context, these youths had higher percentages of adoption, non-traditional family structures, and higher scores on exposure to violence and adverse family circumstances.<br>Depressive state was considered present when youths showed

	youth, family context, and parenting.		- Logistic regression with Nagelkerke's R^2 to analyze the predictive value of each group of variables for the type of crime;<br>- Wald test for the significance of model parameters.	correlated with CPV ($r = -.18$, $p < .001$).<br>Among personal variables, significant predictors of CPV were depression and antisocial personality traits (Nagelkerke $R^2 = .368$). For family variables, exposure to violence and adoption were significant predictors (Nagelkerke $R^2 = .115$). When family circumstances were included in the model, they showed a stronger predictive value than other family variables (Nagelkerke $R^2 = .412$).	signs of emotional instability or depressive symptoms. Exposure to family violence was significant in fostering a hostile social perception and acted as a predictor of CPV. Youth offenders who abused their parents demonstrated a higher-risk psychosocial profile compared to the general offender profile.<br>Depressive symptoms and antisocial personality traits emerged as significant predictors of CPV.<br>**Limitations**:<br>- Moderate sample size but poorly representative of the general population;<br>- Social reluctance to report abuse leading to a reduced sample size;<br>- Assessment conducted without formal clinical diagnosis.
**Cuervo (2025) [29]**	- To identify the predictor variables with the highest explanatory power for CPV;<br>- To develop a general predictive model of	Analysis of juvenile records from the Juvenile Court of a Spanish province, from 2011 to 2017. Recidivism risk was assessed using the Youth Level of Service/Case Management Inventory (YLS/CMI) [78].	**Inferential analyses**:<br>- Chi-square test;<br>- Student's t-test for mean comparisons;<br>- Logistic	Girls were more likely to engage in CPV. Variables such as recidivism, non-traditional family structure, single-parent family, marital conflict, abusive father, depression, denial, poor social skills, and low self-esteem showed statistically	The variables with the greatest predictive power were depressive symptoms, criminal recidivism, and the total YLS/CMI score. It was found that youths classified within the moderate risk group, exhibiting symptoms related to depressive states, were more likely to engage

	CPV and replicate it, examining differences based on participants' sex.			regression;<br>- Conditional inference tree analysis.<br>**Non-parametric analysis:**<br>- Kruskal-Wallis test.	significant associations with CPV ($p < 0.05$).<br>The CPV group presented higher scores on the YLS/CMI and lower scores on protective factors. Within the CPV group, risk scores were 15.98 for girls and 16.75 for boys.<br>Regarding the prediction of FPP the studied variables, the model showed a good fit, with a Nagelkerke R^2 = 0.43 and a non-significant Hosmer-Lemeshow test (p = 0.808). Depression, recidivism, and the total YLS score significantly predicted CPV behavior.<br>Concerning sex, the model also demonstrated good fit, with a Nagelkerke R^2 = 0.37 and a non-significant Hosmer-Lemeshow test (p = 0.163). Depression and recidivism predicted the occurrence of CPV.<br>Regarding the type of crime, the model showed good fit as well, with a Nagelkerke R^2 = 0.72 and a non-significant Hosmer-Lemeshow test (p = 0.979). Parental conflict, total YLS/CMI score, and involvement in denial predicted the occurrence of CPV.	in CPV. For boys, the strongest predictors were depressive state, criminal recidivism, and moderate risk of recidivism. For girls, the main predictors were marital conflict and moderate risk of recidivism.<br>The presence of CPV was generally associated with the total YLS/CMI score and moderate risk of recidivism. Youths with a YLS/CMI score above 8.5 (moderate risk) who presented depressive symptoms had approximately an 80% higher likelihood of engaging in CPV.<br>Exposure to parental conflicts significantly influenced the perpetration of CPV, representing a variable with strong predictive power. A high level of recidivism was associated with CPV, whereas low risk was linked to the commission of other crimes.<br>**Limitations:**<br>- Unknown number of unreported cases, reflecting reluctance to report CPV;<br>- Exclusion of minors outside the juvenile justice system (i.e., under 14 or over 18 years), limiting sample representativeness;<br>- High non-response rate among youths engaging in CPV.

**Cuervo & Palanques (2022) [30]**	- To examine whether there are differences in sociodemographic and criminogenic factors between a group of youths who committed CPV and a group who committed other types of crimes;<br>- To analyze whether the risk factors assessed by the YLS/CMI inventory are the best predictors of CPV.	Analysis of juvenile court records from a Spanish province between 2011 and 2017.<br>Risk factors assessed using the Youth Level of Service/Case Management Inventory (YLS/CMI) [78].<br>Interviews conducted with adolescents and their families.	**Descriptive analysis:**<br>- Frequency analyses of the variables.<br>**Inferential analyses:**<br>- Chi-square tests (for dichotomous variables) and ANOVA (for continuous variables) to examine statistically significant differences;<br>- Binary logistic regression to identify which variables predict CPV.	ex was predominantly male in both groups. Significant differences were found in sex distribution, with males being more prevalent in the comparison group (80.3%) than in the CPV group (63.4%) [$X^2(1) = 12.1$, $p < .001$].<br>A significant difference in recidivism was observed between the two groups, as most youths in the CPV group were recidivists (60.1% vs. 30.3% in the comparison group). Furthermore, the CPV group showed an average of 2.67 cases of any type of crime, while the comparison group had a mean of 1.64. Similarly, the maximum number of accumulated cases in the CPV group was 15, compared to 8 in the comparison group.<br>The greatest differences among risk factors were observed in the subscales Family/Parenting Circumstances, Personality/Behavior, and Substance Abuse. The CPV group exhibited a higher risk of recidivism than the	Youth in the CPV group exhibited higher rates of recidivism (60.1%), a greater number of crimes committed, and higher total scores on the YLS/CMI. The most significant differences among YLS/CMI subscales were found in Family Circumstances, Antisocial Personality, Substance Abuse, and Education. The majority of youths in the CPV group were classified at moderate to high risk levels and showed significantly lower protective factors.<br>The risk subscales predictive of CPV were Family Circumstances, Antisocial Peer Relations, Substance Abuse, and Antisocial Personality. The strongest predictor was Family Circumstances.<br>A deficient family environment, combined with poor family relationships, inadequate supervision, aggressive personality traits, and substance abuse predicted the occurrence of CPV. Criminal history,

				comparison group [F(56) = 2.24, p < .001], with 68.2% of the CPV group classified at moderate recidivism risk, whereas 55% of youths in the comparison group were classified at low recidivism risk. A high recidivism risk was identified in 20.5% of the CPV group, while only 6% of the comparison group reached this level.<br>The comparison group presented significantly higher rates of protective factors than the CPV group in the subscales Peer Relations, Substance Abuse, Personality/Behavior, and Attitudes/Orientation. When analyzing the total sum of protective factors, the CPV group averaged 0.11 protective factors per youth, compared to 0.55 in the comparison group [F(1, 272) = 13.76, p < .001].<br>The subscales Family/Parenting Circumstances, Substance Abuse, and Personality/Behavior predicted the occurrence of CPV. The Antisocial Peers subscale showed a negative association with CPV, indicating that higher	education, and attitudes did not predict CPV.<br>**Limitations:**<br>- Low sample representativeness;<br>- YLS/CMI scores were not reported.

				scores on this subscale reduce the likelihood of belonging to the CPV group. Thus, this subscale predicts general delinquent behavior (comparison group).	
**Del Hoyo-Bilbao et al. (2021) [31]**	- To analyze the longitudinal relationships between dimensions of psychopathy traits and the perpetration of CPV;<br>- To examine whether the predictive model is similar across sexes.	**CPV**: Child-to-Parent Aggression Questionnaire (CPAQ; [4]).<br>**Dimensions of psychopathic traits**: Youth Psychopathic Inventory-Short Form (YPI-S; [83]).	**Analytical Approach:**<br>- Descriptive analyses included frequency distributions and Pearson correlation coefficients for all variables;<br>- Inferential analyses were conducted using robust maximum likelihood estimation (ML) with the Satorra-Bentler scaled chi-square test, implemented in LISREL 8.8.	Psychopathic traits were positively intercorrelated, showing moderate correlations overall, except for a small correlation between irresponsibility and lack of empathy.<br>Females scored higher on irresponsibility ($d = 0.24$) and psychological CPV at both Time 1 (T1) and Time 2 (T2) ($d = 0.61$; $d = 0.54$).<br>Males scored higher on lack of empathy ($d = 0.38$) and egocentrism ($d = 0.23$). Most sex differences had small effect sizes, except for psychological CPV at T1 and T2, which showed moderate to large effects ($d > 0.50$). The model demonstrated good overall fit, particularly with RMSEA < 0.08 and CFI/NNFI > 0.90, and explained psychological CPV well and physical CPV moderately.	Psychopathic traits showed a weak positive association with psychological CPV in cross-sectional analyses. In the longitudinal follow-up, only irresponsibility predicted psychological CPV.<br>The interaction among the three traits predicted physical CPV. Irresponsibility had a greater impact on physical CPV when combined with high lack of empathy, low manipulation, and egocentrism.<br>Psychological CPV predicted physical CPV, indicating a pattern of escalation in violence.<br>Males scored higher on lack of empathy and egocentrism, whereas females scored higher on irresponsibility. Overall, the patterns of association between psychopathic traits and CPV were similar across sexes.<br>Although psychopathic traits individually have a limited effect, their combination can significantly increase physical violence toward parents.<br>**Limitations**:

				Stability of CPV over time was observed, with psychological CPV at T1 predicting physical CPV at T2. The interaction of psychopathic dimensions at T1 predicted CPV at T2. Irresponsibility was related to physical CPV only when lack of empathy was high ($\beta = 0.017$, $t = 2.95$, $p = 0.003$) and when the slope was low ($\beta = 0.027$, $t = 2.82$, $p = 0.005$).<br>The predictive model was invariant across sexes, indicating a similar pattern for males and females ($\Delta\chi^2(12, N = 765) = 11.145$, $p = 0.516$).	- Data were obtained from self-reports by youths, without parental input;<br>- Community sample with low levels of physical violence, highlighting the need for studies with clinical samples and contextual variables
**Espuig et al. (2025) [32]**	- Analyze CPV against mothers and fathers separately, examining associations with sex, age, experiences of physical and verbal-emotional dating violence victimization, psychopathy, and antisocial and law-violating behaviors.	**CPV:** Conflict Tactics Scales (CTS2) – child-to-parent version [84].<br>**Sociodemographic variables:** Personality scale.<br>**Psychopathy:** Psychopathy Content Scale (P-16) [85].<br>**Antisocial and law-violating behavior:** Antisocial and Criminal Behavior Scale in Adolescents (ECADA) [86].<br>**Dating violence victimization:** Conflict in Adolescent Dating Relationships Inventory	**Descriptive Analysis:**<br>- Frequency analyses and Pearson correlations to examine the relationships among dating violence victimization, psychopathy, criminal behavior, age, and CPV.	Low levels of physical violence ($M = 0.29$, $SD = 1$) and verbal-emotional violence ($M = 3.85$, $SD = 5.51$), as well as low-to-moderate levels of psychopathy ($M = 4.02$, $SD = 2.55$), antisocial and law-violating behaviors ($M = 6.54$, $SD = 3.70$), and CPV against the father ($M = 3.06$, $SD = 3.39$) and mother ($M = 3.69$, $SD = 3.39$) were observed.<br>Age was positively associated with greater verbal-emotional victimization ($r = 0.21$, $p \leq 0.05$),	Girls who experienced higher levels of verbal and emotional violence engaged in fewer antisocial behaviors compared to boys and exhibited more CPV against the mother. Psychopathy showed a strong correlation with CPV against both parents, suggesting that youths with elevated psychopathy scores may have difficulties in emotional regulation and reduced empathy, thereby increasing the

	(CADRI) [87].	**Inferential Analyses:**<br>- Hierarchical linear regressions to examine CPV separately against mothers and fathers, controlling for age, sex, physical victimization, verbal-emotional victimization, criminal behavior, and psychopathy.<br>- Qualitative Comparative Analysis using fuzzy sets (fsQCA software) to identify interaction effects and configurations.	higher levels of psychopathy ($r = 0.21$, $p \leq 0.05$), and increased antisocial behaviors ($r = 0.25$, $p \leq 0.01$).<br>CPV against the mother was significantly correlated with verbal-emotional victimization ($r = 0.41$, $p \leq 0.01$), higher psychopathy levels ($r = 0.57$, $p \leq 0.01$), antisocial behaviors ($r = 0.38$, $p \leq 0.01$), and increased CPV against the father ($r = 0.66$, $p \leq 0.01$). In contrast, CPV against the father was associated with higher psychopathy levels ($r = 0.47$, $p \leq 0.01$) and antisocial behaviors ($r = 0.19$, $p \leq 0.05$). The explanatory variables significantly increased the variance explained in CPV against the mother ($\Delta R^2 = 0.18$, $p \leq 0.001$), but not for CPV against the father ($\Delta R^2 = 0.05$, $p > 0.05$). Age ($\beta = -0.21$, $t = 3.2$, $p \leq 0.01$) and verbal-emotional victimization ($\beta = 0.22$, $t = 2.92$, $p \leq 0.01$) were significantly associated with CPV against the mother. When psychopathy and antisocial behaviors were included, the variance explained increased significantly for CPV against the mother ($\Delta R^2 = 0.28$, $p \leq$	likelihood of perpetrating CPV. Antisocial and law-violating behaviors were significantly and positively correlated with CPV, specifically against mothers. Significant correlations were found between verbal-emotional dating violence and CPV. A negative relationship was observed between CPV and age. Predictors of CPV against the mother included older age, female sex, verbal-emotional violence, psychopathy, and antisocial behaviors. Conversely, predictors of CPV against the father were younger age and psychopathy.<br>**Limitations**:<br>- Cross-sectional study design;<br>- Small, convenience sample limiting generalizability;<br>- Data based on self-report without external informants

0.001) and the father ($\Delta R^2 = 0.23$, $p \leq 0.001$). Psychopathy was significantly associated with CPV against both parents (mother: $\beta = 0.48$, $t = 6.33$, $p \leq 0.001$; father: $\beta = 0.52$, $t = 5.93$, $p \leq 0.001$). The final model explained 44% of the variance in CPV against the mother (adjusted $R^2 = 0.44$, $p \leq 0.001$) and 25% against the father (adjusted $R^2 = 0.25$, $p \leq 0.001$). For CPV against the mother, three pathways were identified explaining 63% of the variance. Path 1 (46%) combining female sex, verbal-emotional victimization, and antisocial behaviors. Path 2 (41%) combining female sex, verbal-emotional victimization, and psychopathy. Path 3 (41%) combining female sex, high levels of psychopathy and antisocial behaviors, and low levels of physical and emotional violence.

For CPV against the father, three pathways explained 72% of the variance. Path 1 (55%) combining female sex, antisocial behaviors, and psychopathy. Path 2 (55%) combining female sex,

				psychopathy, and physical and emotional victimization. Path 3 (40%) combining female sex and verbal-emotional victimization.	
**Fandiño et al. (2024) [33]**	- Estimate the prevalence of key recidivism-related variables;<br>- Compare youth who perpetrate CPV with youth who do not engage in CPV offenses.	Self-report questionnaires administered during detention, interviews with family members, and review of judicial and institutional records. Dangerousness assessed using the Masculinity-Femininity and Social Introversion scales of the MMPI-A [88]. Psychopathy traits measured by the Hare Psychopathy Checklist: Youth Version (PCL:YV) [89]. Criminogenic risk factors evaluated with the Youth Level of Service/Case Management Inventory (YLS/CM) [78].	**Validity Analysis of Protocols:**<br>- Inter-rater reliability analysis.<br>**Inferential Analyses:**<br>- Multivariate Analysis of Variance (MANOVA) to compare mean scores of dangerousness, psychopathic traits, and criminogenic risk factos;<br>- Partial eta squared (η^2) to estimate effect sizes;<br>- Probability of Inferiority Score (PIS) index.	Significant differences were found in mental health problems, $F(8, 125) = 1.76$, $p = 0.091$, with a moderate effect size ($0.059 < \eta^2 < 0.137$), partial $\eta^2 = 0.101$, and observed power of 73.7% ($1 - \beta = 0.737$). The prevalence of clinical cases among offenders was significant compared to clinical thresholds for hypochondriasis (illness anxiety) (f = 22; OR = 7.72; Z = 9.56, $p < 0.001$), depression (f = 23; OR = 8.26; Z = 10.12, $p < 0.001$), hysteria (somatic symptom disorder) (f = 22; OR = 7.72; Z = 9.56, $p < 0.001$), psychopathic deviation (f = 44; OR = 26.13; Z = 21.16, $p < 0.001$), paranoia (f = 24; OR = 8.78; Z = 10.64, $p < 0.001$), psychasthenia (generalized anxiety disorder and obsessive-compulsive disorder) (f = 18; OR = 5.90; Z = 7.48, $p < 0.001$), and schizophrenia (social alienation) (f = 20; OR = 6.78; Z = 7.94, $p < 0.001$). A significant multivariate effect was found for psychopathy traits,	Dangerousness is not a distinguishing characteristic of CPV offenders compared to other offenders. However, the prevalence of dangerousness is notably high among CPV offenders, as is the prevalence of psychopathic traits.<br>Superficial, grandiose, and manipulative personality traits, along with callousness and lack of remorse, characterize CPV offenders in comparison to non-offenders.<br>CPV offenders show significantly higher scores in family circumstances (e.g., inadequate supervision, difficulty controlling behavior, poor discipline, inconsistency, weak parental relationships), which contribute to lower self-esteem, physical aggression, tantrums, inattention, low frustration tolerance, feelings of guilt, verbal aggression, and criminogenic risk factors.

				$F(4, 131) = 3.39$, $p = 0.011$. Offenders exhibited higher interpersonal and affective psychopathy traits, with a moderate effect size, showing increases of 28.7% and 23.8% respectively compared to non-offenders. The diagnosis of psychopathy among offenders (25%, n = 19) was significant, $Z = 21.03$, $p < 0.001$, with a large effect size, $h = 1.19$, 95% CI [1.09, 1.29].<br>A significant multivariate effect was also observed in risk factors, $F(8, 127) = 3.34$, $p = 0.002$, with a power of 96.9% ($1 - \beta = 0.969$). Offenders scored higher on family circumstances ($0.20 < d < 0.50$) and personality and behavior risk factors, with moderate effect sizes.	**Limitations:**<br>- Possible absence of responses or inconsistencies in participants with personality disorders regarding mental health assessments;<br>- Reported inconsistencies between the significance and effect size of dangerousness findings;<br>- Limited generalizability due to the specificity of the sample.
**Fandiño et al. (2021) [34]**	**-** To examine whether juvenile CPV offenders exhibit deficits in executive functions and psychological maladjustment;<br>- To quantify the potential impairments and describe their epidemiology.	**Psychological maladjustment**: Minnesota Multiphasic Personality Inventory-Adolescent version (MMPI-A) [90].<br>**Executive functions**: Stroop Color-Word Test [91].	**Descriptive analyses:**<br>- Frequency analyses and one-sample t-tests for psychological dimensions and Stroop Test performance;<br>- Assessment of normality through	Regarding psychological adjustment, significantly higher scores were observed across all scales compared to the normative population. Youth offenders of CPV exhibited 23% more hysterical symptoms, 37% more depressive symptoms, 44% more hypochondria, 68% more psychopathic deviation, 26% more psychasthenia, 24% more schizophrenic symptoms, 17% more hypomanic symptoms, and 13%	Clinically, these youth exhibited more symptoms in the core clinical dimensions than the normative population, showing clinical deterioration only in psychopathic deviation (which includes traits such as unreliability, egocentrism, inability to learn from experience, problems with family/authorities, anger in

	skewness and kurtosis;<br>- Calculation of Cohen’s d for effect size.<br>**Inferential probabilistic analysis:**<br>- Binomial effect size measured using r;<br>- Analysis of Z and T scores.	more social introversion. The effect size (indicating impairment in mental health markers) was greater than 26.6% for hypochondria, 43.2% for depression, 51.6% for hysteria, 80.2% for psychopathic deviation, 53.4% for paranoia, 30.4% for psychasthenia, 27.4% for schizophrenia, 19.8% for hypomania, and 14.2% for social introversion. Epidemiologically, the percentage of clinical deterioration (T ≥ 66.45, i.e., 95th percentile) was higher than expected (5% in the normative sample). Effect sizes indicated rates above expected normative values (5%) of 82.1%, 82.9%, 82.9%, 91.7%, 83.7%, 77.9%, and 80.2% for hypochondria, depression, hysteria, psychopathy, paranoia, psychasthenia, and schizophrenia, respectively.<br>Regarding executive functions, youth who committed CPV showed greater impairment in word reading, color naming, color-word naming, and interference on the Stroop Test. The magnitude of impairment was 62% for reading, 47.9% for naming, 45.8% for color-word naming, and 11.9% for interference. Case studies revealed a significantly higher prevalence of impairment compared	relationships and under stress), and moderate deterioration in hysteria (naïve, egocentric, exhibitionist, extroverted, and superficial) and paranoia (sensitivity to criticism and personalizing others’ actions).<br>Youth offenders of CPV show greater deterioration compared to other juvenile justice samples.<br>Epidemiologically, the prevalence of clinical and moderate deterioration cases was very high across all clinical dimensions, excluding hypomania and introversion.<br>The Stroop test results indicate impairments in the ability to inhibit responses to stimuli requiring suppression of automatic responses, reduced planning and execution capabilities, low cognitive flexibility, and dysfunction in cognitive inhibition.<br>**Limitations**:<br>- Low sample representativeness;<br>- Sensitivity of the case study design was α/β 6 = 1;<br>- Presence of defensiveness in

					to the general population, except for interference.	responses, potentially introducing bias;<br>- Stroop tasks are relatively simple in experimental settings.
**Fongaro et al. (2023) [35]**	-	To examine the clinical characteristic s of children and adolescents who have committed physical or verbal CPV, as well as the sociodemogr aphic and general characteristic s of their families.	Semi-structured diagnostic interview K-SADS [92] was used to assess current and past episodes of psychopathology in children and adolescents.	tatistical analyses were conducted using SAS (Enterprise Guide 8.2).<br>**Descriptive analyses:**<br>- Frequency analyses of sociodemographic and clinical characteristics of parents and children.	The mean age of the children was 11.71 years, ranging from 7 to 17 years, with 57.32% being male.<br>Fifty-five point fifty-six percent (55.56%) of the children lived with both parents, and 8.22% lived in single-parent families.<br>Regarding social status, the majority of parents were professionals and executives with higher intellectual levels.<br>Eighty-four point seventy-two percent (84.72%) of the children had previously received psychiatric or psychological care.<br>There was a high prevalence of ADHD (69.86%) and current anxiety-related disorders (49.32%).<br>Specifically, 38.36% met criteria for agoraphobia; 26.03% for generalized anxiety disorder; 19.18% for social anxiety disorder; 16.44% for depressive disorder; 41.10% for sleep disorder; 56.16% for disruptive mood dysregulation disorder; and 10.96% for separation anxiety disorder.	A minority of children met criteria for Conduct Disorder, confirming the presence of violence and a restricted tyrannical behavioral dynamic within the family environment.<br>The most significant difference observed was the higher proportion of mothers with elevated social status compared to the general French population.<br>Boys appeared to be at greater risk for severely tyrannical behavior.<br>Children exhibiting severely tyrannical behavior showed high levels of comorbid psychiatric conditions, with elevated scores in ADHD and aggressive behavior.<br>Anxiety problems in children with severely tyrannical behavior were also manifested as social phobia and agoraphobia, in addition to sleep disorders. Some children presented with Obsessive-Compulsive Disorder and Depressive Disorders.<br>**Limitations**:<br>- This was a purely analytical study, thus correlations between variables could not be determined.

**Gámez-Guadix & Calvete (2012) [36]**	- To analyze the relationship between exposure to different types of family violence and the perpetration of physical and psychological CPV.<br>- To examine whether the relationships between various types of domestic violence differ between boys and girls.	**CPV:** Child-to-Parent Violence Scale developed based on the Conflict Tactics Scales (CTS2 and CTSPC).<br>**Parental violence:** Revised Conflict Tactics Scale (CTS2).<br>**Physical aggression from parents to children:** Modified Physical Abuse Scale from the Parent-Child Conflict Tactics Scales (CTS-PC) [93].<br>**Psychological aggression:** Dimensions of Discipline Inventory, Form A (DDI) [65], Psychological Aggression subscale.	**Statistical analyses** were conducted using EQS 6.1 software (Bentler, 2005).<br>**Descriptive analyses:**<br>- Frequency analyses and Pearson correlations for the study variables<br>**Inferential analyses:**<br>- Robust maximum likelihood estimation method with Satorra-Bentler scaled chi-square (χ^2) for variable normality assessment;<br>- Standardized root mean square residual (SRMR) and robust versions of the Non-Normed Fit	All correlations between variables were significant ($p < 0.001$). The highest correlation was 0.47 between intraparental physical aggression and parent-to-child physical aggression.<br>No significant differences were found between boys and girls in mean scores for exposure to psychological intraparental violence [t(1670) = -0.87, ns] or physical intraparental violence [t(1670) = 1.58, ns], nor in psychological parental aggression [t(1670) = -0.83, ns] or physical parental aggression [t(1670) = 1.12, ns].<br>Psychological aggression toward children was higher in girls (M = 2.45, SD = 1.59) than boys (M = 2.08, SD = 1.46) [t(1670) = 4.19, $p < 0.001$]. Conversely, physical aggression was higher in boys (M = 0.12, SD = 0.36) than girls (M = 0.08, SD = 0.28) [t(1670) = 2.38, $p < 0.05$].<br>**The final estimated model demonstrated good fit**: Satorra-Bentler $\chi^2(65, N = 1681) = 137.12$; RMSEA = 0.026 (90% CI = 0.020 – 0.032); SRMR = 0.035; CFI = 0.95;	Both exposure to interparental violence and parent-to-child aggression (physical and psychological) were associated with the future occurrence of CPV.<br>Higher frequency of parent-to-child aggression was related to an increased likelihood of CPV. Consistent relationships were found across all types of violence, such that intraparental psychological aggression and parent-to-child psychological aggression were associated with psychological CPV, while intraparental physical aggression and parent-to-child physical aggression were associated with physical CPV. A significant association was observed between intraparental aggression and parent-to-child aggression (both physical and psychological).<br>The relationship between exposure to domestic violence and CPV was similar across both sexes.<br>**Limitations**:<br>- Results were based on

			Index (NNFI), Comparative Fit Index (CFI), and Root Mean Square Error of Approximation (RMSEA);<br>- Multi-group analysis to examine invariance across groups.	NNFI = 0.91. The model explained 21% of the total variance in psychological CPV and 27% in physical CPV.<br>Various types of violence between parents and from parents to children were related, with correlation magnitudes ranging from moderate ($r = 0.34$, $p < 0.001$; psychological aggression between parents – physical aggression from parents to children) to high ($r = 0.54$, $p < 0.001$; physical aggression between parents – physical aggression from parents to children).<br>**Model fit was adequate for both sexes**:<br>**Girls**: S-B $\chi^2(65, N = 1293) = 141.70$; RMSEA = 0.030 (90% CI = 0.023 – 0.037); SRMR = 0.043; CFI = 0.93; NNFI = 0.90<br>**Boys**: S-B $\chi^2(63, N = 379) = 66.14$; RMSEA = 0.007 (90% CI = 0.000 – 0.032); SRMR = 0.039; CFI = 0.99; NNFI = 0.98	retrospective self-reports;<br>- Limited representativeness of the sample.
**Harries et al. (2022) [37]**	- To compare different categories of parental physical force in relation to CPV	**CPV:** Conflict Tactics Scale (CTS) [94].<br>**Corporal punishment:** Brief Physical Punishment Scale (BPPS) [95].	**Descriptive analysis:**<br>- Frequency analyses of the study groups.	Corporal punishment and physical abuse showed a strong and positive correlation ($r = 0.80$). CPV against both parents was also highly positively correlated (r =	The frequency of CPV increased as the level of corporal punishment experienced increased.<br>Individuals who experienced

	outcomes against both parents.	**Physical abuse:** Childhood Trauma Questionnaire – Short Form, Physical Abuse subscale (CTQ-SF) [96].	**Inferential analyses:**<br>- One-way ANOVA to assess mean scores of CPV against mothers and CPV against fathers;<br>- Kolmogorov–Smirnov test and Shapiro–Wilk test to assess normality.	0.48).<br>For mothers, the results revealed a significant overall effect, $F(2, 297.24) = 6.27$, $p = 0.002$. Similarly, for fathers, a significant overall effect was observed, $F(2, 522.44) = 5.91$, $p = 0.003$.	high levels of corporal punishment in childhood were likely to use similar levels of violence against their parents as those who experienced high levels of both physical abuse and corporal punishment, suggesting that physical abuse does not contribute to an additional increase in CPV among youths who were also exposed to corporal punishment. Corporal punishment and physical abuse were likely to co-occur.<br>**Limitations:**<br>- Cross-sectional study design;<br>- Use of self-report measures based on retrospective recall, which may introduce recall bias;<br>- Limited details regarding the nature of CPV associated with parental use of physical force;<br>- Australian sample, where corporal punishment is not considered a criminal offense and is therefore socially minimized.
**Ibabe (2014) [38]**	- To examine the effect of family violence on adolescents'	**Maladjustment:** Multifactor Self-Assessment Test of Child Adjustment [97].	**Descriptive analyses:**<br>- Frequency	Twenty-one percent of adolescents reported having engaged in physical CPV, 33% in psychological CPV, and	Both **p**arent-to-child violence and intimate partner violence contributed to explaining the

	maladjustment and CPV;<br>- To explore the potential mediating effect of adolescents' behavioral and/or emotional problems on the relationship between family violence and CPV.	**Behavior and personality:** Behavior Assessment System for Children and Adolescents (BASC; [92]).<br>**Substance use:** Millon Clinical Inventory for Adolescents [103].<br>**Family violence:** Scale of Intra-family Violence [51].	analyses of the types of CPV and types of violence.<br>**Inferential analyses:**<br>- One-way ANOVA;<br>- Tukey post hoc test;<br>-Maximum likelihood analysis.<br>**Model fit assessment:**<br>- EQS 6.1 (Structural Equation Modeling software).	19% in financial CPV. Physical CPV was significantly correlated with intimate partner violence ($r = 0.42$) and moderately correlated with parent-to-child violence (father: $r = 0.41$; mother: $r = 0.44$). Financial CPV showed moderate correlations with intimate partner violence ($r = 0.51$) and with parent-to-child violence (father: $r = 0.40$; mother: $r = 0.45$). Financial violence was also correlated with personal maladjustment ($r = 0.24$), school maladjustment ($r = 0.24$), and social maladjustment ($r = 0.22$). In Model 2, goodness-of-fit indices indicated an adequate model fit:<br>ML $\chi^2(83, N = 485) = 231.79$, CFI = 0.95, NNFI = 0.93, IFI = 0.95, RMSEA = 0.061;<br>Yuan–Bentler $\chi^2(83, N = 485) = 165.50$, CFI = 0.96, NNFI = 0.95, IFI = 0.96, RMSEA = 0.045. In the structural model, overall fit was acceptable: ML $\chi^2(95, N = 485) = 277.0$, $p < 0.001$, CFI = 0.94, NNFI = 0.92, IFI = 0.94, RMSEA = 0.063. The model explained 75% of the variance in parent–child violence. All factor loadings were statistically significant ($p < 0.001$), except for lack of discipline ($p < 0.01$). Intra-family violence had direct effects on emotional problems ($\beta = 0.46$, $p <$	occurrence of parent–child violence observed in the sample. Family violence predicted different forms of adolescent maladjustment. Negative family relationships (i.e., lack of cohesion and high levels of conflict) showed direct effects on the development of antisocial behavior in youth, and antisocial behavior, in turn, predicted violent behavior toward authority figures. Exposure to family violence had direct effects on emotional problems (somatization, depression, and low self-esteem) and behavioral problems (substance use and aggressive behaviors). Both emotional and behavioral problems predicted CPV, with behavioral factors emerging as the strongest predictors. It was also observed that, as age increased, behavioral problems increased, whereas parent–child violence decreased.<br>**Limitations:**<br>- Cross-sectional study design;<br>- Possible framing effects.

				0.001), behavioral problems (β = 0.52, p < 0.001), and parent–child violence (β = 0.45, p < 0.001). The latent variable behavioral problems was a significant predictor of CPV (β = 0.53, p < 0.001). Age emerged as a significant predictor of CPV (β = −0.25, p < 0.001) and behavioral problems (β = 0.46, p < 0.001). Adolescents aged 12–14 years exhibited significantly more violent behavior toward parents compared to those aged 17–18 years. Age was also significantly associated with substance abuse, F(2, 463) = 15.40, p < 0.001; alcohol consumption, F(2, 461) = 32.90, p < 0.001; illegal substance use, F(2, 441) = 5.62, p = 0.004; and social aggression, F(2, 477) = 5.64, p = 0.004. Emotional problems were more frequent among adolescents who witnessed and/or experienced family violence, including somatization, t(321) = −3.62, p < 0.001, d = 0.17, 95% CI [−1.40, −0.41]; depression, t(321) = −3.03, p = 0.003, d = 0.14, 95% CI [−0.57, −0.12]; and low self-esteem, t(184.05) = −4.54, p < 0.001, d = 0.21, 95% CI [−1.41, −0.57].	
**Ibabe et al. (2013) [39]**	- To determine the role of family relationship quality and family discipline strategies,	**CPV:** Conflict Tactics Scale Child-to-Parent (CTS1) ([104]. **Intimate Partner Violence:** Revised Conflict Tactics Scale (CTS2) [105].	**Descriptive analyses:** - Frequency analysis, chi-	Five percent of adolescents reported having perpetrated severe physical violence against one of their parents, 11% reported mild physical	Marital violence is a risk factor for CPV. Parental coercive discipline strategies were moderately associated with

	moderated by the level of coercion, in the development of violent behaviors and prosocial behaviors of adolescents toward their parents.<br>- To examine differences between various family types in relation to CPV.	**Family Discipline:** Discipline Dimensions Inventory (DDI-C) [65].<br>**Family Climate:** Family Environment Scale (FES) [106].<br>**Prosocial Behaviors in the Family:** Scale of Behaviors toward Authority Figures [107].	square tests, and Pearson correlations to determine the prevalence of CPV and marital violence.<br>**Inferential analyses:**<br>- Maximum likelihood estimation;<br>- Confirmatory factor analysis.<br>**Model fit assessment:**<br>- EQS 6.1 (Structural Equation Modeling software).	violence, and 13% reported both types. Physical abuse ranged from 1% hitting to 9% pushing. No differences were found in the perpetration of physical aggression based on the sex of either the parents or the adolescents. Higher rates of CPV were found in single-mother households (21%) $\chi^2(1, N = 481) = 4.94$, $p = 0.026$, $r = 0.10$, in reconstituted families (22%) $\chi^2(1, N = 473) = 5.60$, $p = 0.018$, $r = 0.11$, and in other family types $\chi^2(1, N = 450) = 10.46$, $p < 0.001$, $r = 0.15$. Eighty-eight percent reported psychological CPV, varying from 16% threatening and hitting to 84% yelling. Psychological CPV was more common towards mothers (84%) than fathers (81%), $\chi^2(1, N = 545) = 223.91$, $p < 0.01$, $r = 0.64$. Girls were more involved in this type of CPV against mothers (88%) compared to boys (82%). Regarding marital violence, 7% reported unilateral violence and 4% reciprocal violence. Maternal violence against the father (5%) was higher than paternal violence against the mother (2%), $\chi^2(1, N = 43) = 6.72$, $p < 0.01$. Physical and psychological CPV were significantly associated with higher levels of marital violence ($r = 0.45$ and $r = 0.24$, respectively), family	parent-child violence. Marital violence significantly predicted physical CPV, while a positive family climate was associated with lower levels of violent behavior by children toward their parents.<br>Coercive strategies were positively linked to physical violence between parents and children. The use of coercive parental discipline strategies may contribute to violent behavior in children. These coercive discipline strategies were more frequently employed in immigrant families.<br>Family cohesion, low conflict levels, and organization increased the likelihood of adolescents exhibiting prosocial behaviors toward their parents.<br>Coercive family discipline strategies are associated with higher levels of parent-child violence, whereas a positive family climate predicts lower levels of such violence and more prosocial behaviors.<br>**Limitations:**<br>- Cross-sectional design;

	conflict (r = 0.20 and r = 0.48), more frequent coercive strategies (r = 0.27 and r = 0.58), response cost (r = 0.10 and r = 0.27), and supervision (r = 0.38 and r = 0.42), as well as lower cohesion (r = –0.21 and r = –0.42) and family organization (r = –0.14 and r = –0.27). Adolescents' prosocial behaviors toward their parents were related to lower levels of marital violence (r = –0.12), family conflict (r = –0.41), and parental coercive strategies (punishment r = –0.36; supervision r = –0.21), as well as a more positive family climate (cohesion r = 0.47; organization r = 0.34). Positive discipline was not related to either CPV or prosocial behaviors.<br>The model demonstrated reasonable fit: ML χ^2(82, N = 585) = 243.11; NNFI = 0.92, CFI = 0.94, IFI = 0.94, RMSEA = 0.058. The model explained 45% of the variance in CPV. A positive family climate predicted lower CPV (β = –0.67, p < .001) and coercive discipline (β = –0.58, p < .001), and higher prosocial behavior (β = 0.72, p < .001). Marital violence predicted greater parent-child physical violence (β = 0.42, p < .001), poorer family climate (β = –0.31, p < .001), and lower use of	- Possible framing effects and social desirability bias.

				positive discipline ($\beta = -0.18$, $p < .001$).	
**Ibabe et al. (2014) [40]**	- To examine externalizing and internalizing problems in children and adolescents accused of CPV, comparing them with those who committed other offenses and with non-offending youth;<br>- To explore sex differences in the manifestation of psychological problems among youth, as well as different types of violence between parents and children.	**Family violence**: Intra-family Violence Scale Adjustment: Multifactor Self-Assessment Child Adjustment Test (TAMAI) [97].<br>**Behavior**: Behavior Assessment System for Children (BASC) [98].<br>**Personality traits**: Millon Adolescent Clinical Inventory (MACI) [99].<br>**Attention deficit and hyperactivity**: Magallanes Scale of Identification for Attention Deficit.	**Descriptive analyses:**<br>**-** Frequency analysis of the study variables according to group membership.<br>**Inferential analyses:**<br>**-** Games-Howell post-hoc tests for multiple comparisons;<br>- Confirmatory factor analysis.	Seventy-three percent of CPV offenders reported having used physical violence against their parents. Additionally, 29% of other offenders and 16% of non-offending adolescents also engaged in CPV. Fifty-three percent of CPV offenders reported economic violence, compared to 21% of other offenders and 21% of non-offenders.<br>Youth who committed CPV, compared to other offending youth, showed higher rates of physical violence, $\chi^2(1, N = 89) = 10.46$, $p = 0.001$, $\varphi = 0.44$; psychological violence, $\chi^2(1, N = 89) = 10.66$, $p = 0.001$, $\varphi = 0.44$; as well as financial violence, $\chi^2(1, N = 96) = 7.07$, $p = 0.008$, $\varphi = 0.33$.<br>Model fit indices were adequate: ML $\chi^2(70, N = 231) = 111.87$, CFI = 0.99, NNFI = 0.98, IFI = 0.98, RMSEA = 0.051; Yuan-Bentler $\chi^2(70, N = 231) = 106.42$, CFI = 0.99, NNFI = 0.99, IFI = 0.99, RMSEA = 0.048.<br>CPV was moderately associated with depressive symptomatology	The group of youths who committed CPV exhibited higher levels of school maladjustment (such as school indiscipline and aversion to teachers) and social maladjustment (social aggression).<br>Both offender groups scored highly on several behavioral problems, including propensity for substance abuse and illegal drug use, hyperactivity, attention deficit, dysnomia, and social self-maladjustment.<br>The CPV offenders' profile also showed elevated levels of personal maladjustment, with a high incidence of symptoms associated with depressive states.<br>The rate of behavioral problems outside the family context was lower among females. Behavioral problems (hyperactivity, indiscipline, social aggression, and substance use) outside the home were significant predictors of CPV.<br>Children and adolescents who

				($r = 0.39$, $p < 0.001$) and behavioral problems ($r = 0.55$, $p < 0.001$). Depressive symptomatology and behavioral problems outside the home were also significantly correlated ($r = 0.56$, $p < 0.001$).<br>Model fit indices were acceptable: ML $\chi^2(71, N = 231) = 110.65$, $p < 0.001$, CFI = 0.99, NNFI = 0.98, IFI = 0.98, RMSEA = 0.049. The model explained 30% of the variance in CPV.<br>Behavioral problems significantly predicted CPV ($\beta = 0.47$, $p < 0.001$), while depressive symptomatology did not ($\beta = 0.13$, $p > 0.05$). A positive relationship was observed between depressive symptoms and behavioral problems in both sexes ($r = 0.61$, $p < 0.001$).<br>Female sex was a significant predictor of emotional CPV ($\beta = 0.17$, $p < 0.001$) and fewer behavioral problems ($\beta = -0.22$, $p < 0.001$).	commit CPV are characterized by multiple types of maladjustment, emotional imbalance associated with depressive states, and family dissatisfaction.<br>**Limitations:**<br>- Cross-sectional study design, which does not allow causal relationships to be established;<br>- Issues regarding data validity.
**Ibabe & Jaureguizar (2010) [41]**	- Identify the main risk factors of youth who physically and verbally abuse their parents, in	Data were collected from case files of the Public Prosecutor's Office for Children and Youth in Bilbao (Basque Country, Spain), covering the period between 1999 and	**Descriptive analyses:**<br>- Frequency analyses to examine the	There was an increase in reported cases starting from 2002, with the percentage of accusations rising from 0.02% to 3.7% in 2005. The reasons for reporting included	A higher proportion of youths involved in CPV lived in nuclear families and were firstborn children, presenting more school problems and lower self-esteem,

	comparison with other juvenile offenders;<br>- Examine sex differences between perpetrators and victims.	2006. Clinical diagnostic information was provided by the youths' parents.	progression of parental abuse;<br>- Pearson's chi-square test to identify relationships between groups.<br>**Inferential analyses:**<br>- Analysis of variance (ANOVA);<br>- Bivariate relationships between youth risk factors according to group.	both physical and psychological violence. In 67% of the cases, both types of violence occurred, with 29% involving physical abuse and 4% psychological violence.<br>Regarding the victim, differences were found depending on the sex of the aggressor [χ^2(2, N=67) = 12.99; $p < 0.01$; asymmetric $\lambda = 0.00$ victim, 0.18 sex]. The most frequent victim was the mother (97%), who was more often assaulted by sons (48%) than daughters (10%).<br>Adolescents in the CPV group more frequently lived in single-parent families compared to those in the non- CPV group [χ^2(4, N=102) = 23.45; $p = 0.001$; asymmetric $\lambda = 0.25$ household, group = 0.28].<br>Youth in the CPV group (M = 1.5) were more often the first-born child than those in the non- CPV group (M = 1.8).<br>There was a moderate negative correlation between economic status and the number of offenses committed, r(93) = -0.47, $p < 0.001$.<br>hirty-three percent of adolescents witnessed domestic violence, and	and were more likely to have received individual and family psychological treatment. Males were more frequently reported for domestic violence offenses than females. Violence among females was more selective, as it could be directed either toward the father or the mother.<br>Greater substance abuse was observed among adolescents who committed CPV.<br>Seventy-seven percent of the cases involved a non-clinical diagnosis. The most common diagnoses were ADHD and Disruptive Behavior Disorder, comprising 77% of all diagnosed youths. Personality disorders accounted for 14% of the youths who committed CPV, including Schizoid Personality Disorder and Borderline Personality Disorder. Other identified disorders included substance dependence, anxiety disorders, and mood disorders.<br>**Limitations**:<br>- The psychological profiles were based on evaluations conducted

				38% of the youth in the CPV group experienced family violence.<br>The correlation between family violence and parental abuse approached significance, $\varphi(102) = -0.18$, $p = 0.06$.<br>Self-esteem was significantly lower in the CPV + and CPV groups ($F = 7.39$, $p < 0.01$). Autonomy was lower in CPV + ($F = 11.70$, $p < 0.001$), and empathy was reduced in both CPV + and CPV groups ($F = 13.42$, $p < 0.001$).<br>Eighty-six percent of the youths consumed some type of legal or illegal substance, with cannabis use being more prevalent in the CPV + group ($\chi^2 = 4.11$, $p < 0.05$).<br>Violent behavior towards adults was more frequent in the CPV group ($\chi^2 = 21.52$, $p < 0.001$).<br>Learning difficulties, adaptation problems, and disruptive behavior were significantly greater in the CPV group ($p < 0.001$).	by the judicial psychosocial team psychologists and social services, relying on information provided by the youths' parents.
**Izaguirre & Calvete (2017) [42]**	- To examine predictive associations between exposure to domestic violence and both CPV and dating violence.	**CPV**: Child-to-Parent Aggression Questionnaire [4].<br>**Exposure to violence**: Exposure to Violence Questionnaire [63].	**Descriptive analyses:**<br>- Frequency analyses for all types of violence	Exposure to violence against mothers was similar across sexes, except for physical assaults, which were more frequently reported by girls. Physical victimization by	Direct victimization was more relevant for CPV, whereas witnessing violence against the mother was more relevant for dating violence. Adolescents

	**Aggressiveness in dating relationships:** Conflict in Adolescent Dating Relationships Inventory [87].	and comparisons by sex.<br>**Model fit indices:**<br>- Comparative Fit Index (CFI);<br>- Non-Normed Fit Index (NNFI);<br>- Root Mean Square Error of Approximation (RMSEA);<br>- Standardized Root Mean Square Residual (SRMR);<br>- Multi-group analysis.	parents was higher among boys. A greater percentage of boys reported sexual abuse against their partners, while girls reported more frequent physical aggression toward partners. The prevalence of psychological abuse toward parents was higher in boys than in girls.<br>In the model, the lowest coefficients were found in the associations between dating violence measures and witnessing violence against the mother, whereas the highest coefficients were observed between direct victimization by parents and corresponding measures of child-to-parent violence (CPV).<br>Victimization by the mother predicted an increase in CPV toward the mother, and victimization by the father predicted CPV toward the father. Victimization by dating violence predicted increased perpetration of dating violence.<br>Model fit indices were adequate: $\chi^2(22, N=606) = 38$, RMSEA = 0.035, 90% CI = [0.015, 0.053], p = 0.89, CFI = 0.99, NNFI = 0.99, SRMR = 0.025.<br>The model explained 30%, 24%, 18%, and 29% of the variance in violence between children and mothers, children and fathers, dating violence perpetration, and	who acted aggressively in one type of close relationship (i.e., parents) tended to act aggressively in other close relationships (i.e., romantic relationships).<br>Direct victimization by the mother predicted an increase in CPV toward mothers, and direct victimization by the father predicted CPV toward fathers. This suggests that youths' aggressive reactions may represent previous aggression experienced from their parents. Various sources of victimization may serve as antecedents of CPV.<br>Victimization by dating violence predicted an increase in the perpetuation of dating violence, with a tendency to increase with age. Witnessing violence against the mother was not associated with victimization by dating violence in boys; however, in girls, witnessing violence against the mother and being maltreated by the father predicted victimization by dating violence.<br>Both boys and girls perpetrate

				dating violence victimization, respectively.<br>For girls, witnessing domestic violence against the mother predicted an increase in dating violence perpetration, and direct victimization by the father predicted victimization by dating violence.<br>Model fit was adequate: $\chi^2(18, N=324) = 55$, NNFI = 0.95, CFI = 0.98, RMSEA = 0.06, 90% CI = [0.04, 0.07], SRMR = 0.03.<br>In the model including both sexes, fit was good: $\chi^2(34, N=606) = 120$, NNFI = 0.95, CFI = 0.98, RMSEA = 0.07, 90% CI = [0.05, 0.08], SRMR = 0.04.<br>The only significant sex difference was in the path from victimization by the mother to CPV toward the mother, $\Delta\chi^2(1, N=606) = 4$, $p = 0.04$, indicating this path was stronger in girls than boys.	physical and psychological CPV. Girls scored higher on the frequency of CPV against mothers, and boys against fathers.<br>**Limitations**:<br>- Exclusive use of self-report inventories;<br>- Short time interval between assessments;<br>- Witnessing of domestic violence from mother to father or parental aggression toward siblings was not assessed;<br>- The study did not include mechanisms explaining longitudinal associations between variables.
**Jiménez-Granado et al. (2023) [43]**	- To evaluate the moderating effects of Borderline Personality traits and psychopathic traits in adolescents on the predictive association between maladaptive parenting strategies and child-to-parent violence;	**CPV:** Assessed using The Revised Child-to-Parent Aggressions Questionnaire (CPAQ-R) [100].<br>**Maladaptive Parenting Strategies:** Measured by The Dimensions of Discipline Inventory – Child-report (DDIC) [65].<br>**Psychopathic Traits:** Evaluated through The Youth Psychopathic Inventory – Short Version (YPI-S).<br>**Borderline Personality Traits:** Assessed using The Personality	**Descriptive analyses:**<br>- Frequencies and Spearman's rho correlations were conducted.<br>**Inferential analyses:**<br>- Structural equation modeling (SEM) was performed using	Girls scored higher on parental discipline strategies involving psychological aggression, as well as on impulsive-irresponsible and borderline personality traits.<br>The overall model demonstrated good fit indices: Satorra-Bentler $\chi^2(173, N = 671) = 643.07$, RMSEA = 0.063, 90% CI [0.057, 0.068], CFI = 0.96, NNFI = 0.95. The model explained 33% of the variance CPV	Key predictive variables of CPV were psychological aggression toward the child (predicting CPV against both parents) and ignoring misbehavior (predicting CPV against mothers).<br>No significant direct predictive association was found between psychopathic traits and CPV in the longitudinal model.

	- To examine the interaction effects in the development of CPV perpetrated against fathers and mothers.	Belief Questionnaire – Short Form (PBQ-SF) [101].	LISREL 8.8 (Jöreskog & Sörbom, 2006) with Robust Maximum Likelihood estimation (RML).	against fathers and 42.1% against mothers.<br>Psychological aggression toward the child predicted an increase in CPV against fathers, while ignoring misbehavior predicted an increase in CPV against mothers.<br>Ignoring misbehavior significantly predicted an increase in CPV against the father among adolescents scoring high on the callous-unemotional trait ($\beta = 0.217$, $t = 4.26$, $p < 0.000$). A significant increase in CPV against the mother was also predicted by high callous-unemotional traits ($\beta = 0.271$, $t = 6.29$, $p < 0.001$).<br>Parental psychological aggression predicted a significant increase in CPV against the father among adolescents scoring low on borderline personality traits ($\beta = 0.163$, $t = 2.95$, $p = 0.003$).<br>Model for boys**:** S-B $\chi^2(147, N = 320) = 258.49$, RMSEA = 0.049, 90% CI [0.039, 0.059], CFI = 0.98, NNFI = 0.96. The boys' model explained 39.2% and 41.2% of the variance in CPV against fathers and mothers, respectively.<br>Model for girls: S-B $\chi^2(155, N = 341) = 399.58$, RMSEA = 0.068, 90% CI [0.059, 0.076], CFI = 0.97, NNFI = 0.95. The girls' model explained	No direct predictive association was found between borderline personality traits and CPV.<br>The interaction between ignoring misbehavior and callous-unemotional traits predicted CPV against both parents, indicating that the predictive association between ignoring misbehavior and CPV against the mother is stronger at higher levels of callous-unemotional traits.<br>Adolescents exposed to high parental psychological aggression and low borderline personality traits exhibited higher CPV scores.<br>The interaction between ignoring misbehavior and callous-unemotional traits in predicting CPV against mothers was significant only for girls.<br>Conversely, the interaction between corporal punishment and borderline traits was significant only for boys.<br>**Limitations:**<br>- Retrospective data collection, with potential recall bias;

				38.3% and 39.2% of the variance in CPV against fathers and mothers, respectively.<br>The interaction between ignoring misbehavior and callous-unemotional traits in predicting CPV against mothers was significant for girls. The interaction between corporal punishment and borderline personality traits in predicting CPV against mothers was significant for boys.<br>For girls, ignoring misbehavior predicted an increase in CPV against mothers in those with high callous-unemotional traits (β = 0.454, t = 6.92, p < 0.001). For boys, ignoring misbehavior was marginally associated with increased CPV against mothers (β = 0.120, t = 1.95, p = 0.052).<br>For girls, corporal punishment did not predict increased CPV against mothers at either low (β = 0.004, t = 0.057, p = 0.954) or high (β = 0.000, t = 0.000, p = 1.000) levels of borderline traits. For boys, corporal punishment predicted an increase in CPV against mothers for those with high borderline traits (β = 0.064, t = 0.788, p = 0.431).	- The average age of participants was lower than the typical peak age for CPV behaviors.
**Junco-Guerrero et al. (2022) [44]**	- To analyze the direct and indirect effects of exposure to domestic	**CPV**: Child-to-Parent Aggression Questionnaire (CPAQ) [4].	**Descriptive analyses:**	A total of 25.4% of adolescents reported having committed severe psychological aggression against	Regarding CPV against the mother, a direct link was observed between witnessing

	violence, emotional insecurity within the family, and the justification of violence on CPV against both parents.	**Emotional security**: Security in the Family System Scale (SIFS) [102].<br>**Justification of violence**: Adolescents' Irrational Belief Scale (ECIA) [62].<br>**Exposure to violence**: Exposure to Violence Questionnaire (CEV) [63].	- Frequency analysis for exposure to violence, emotional insecurity in the family, justification of violence, and CPV;<br>- Pearson correlation to assess the pattern of relationships.<br>**Inferential analyses**:<br>- Tucker-Lewis Index (TLI);<br>- Asymptotically Distribution-Free (ADF) estimation.	their mother, and 20% against their father. Severe physical aggression was admitted by 2.2% against the mother and 1.7% against the father.<br>A global relationship was observed between the youths' sex, domestic violence exposure, emotional insecurity, justification of violence, and types of CPV.<br>The model predicting CPV against the mother indicated that higher rates of exposure to violence—both as victims and witnesses—were associated with higher levels of disengagement ($\beta = 0.18$, $p < 0.001$ and $\beta = 0.19$, $p < 0.001$, respectively). Disengagement was related to higher levels of justification of violence ($\beta = 0.24$, $p < 0.001$).<br>Direct associations were found between exposure to violence as a witness and justification of violence ($\beta = 0.11$, $p < 0.05$), as well as between exposure as a victim and CPV against the mother ($\beta = 0.19$, $p < 0.01$).<br>Male sex was associated with higher levels of disengagement and CPV ($\beta = 0.10$, $p < 0.01$; $\beta = 0.10$, $p < 0.01$), whereas female sex was linked to higher levels of justification of violence ($\beta = -0.19$, $p < 0.001$).<br>Model fit indices were $\chi^2(6) = 12.75$,	violence and justification of violent behavior, whereas no direct association was found in the case of being a victim of violence. A direct association was found between victimization at home and CPV against the mother. The effect of witnessing violence on CPV against the mother was mediated by emotional insecurity and justification of violence. Disengagement from the family system was related to both justification of violence and CPV against the mother. There was a direct relationship between justification of violence and CPV. A significant association was found between female sex and the use of disengagement and higher CPV, and between male sex and greater justification of violence.<br>Regarding CPV against the father, exposure to violence was related to disengagement from the family system and to concern. An association was observed between witnessing violence and the perpetration of

					p = 0.057. The model explained 32% of the variance in CPV against the mother.<br>For CPV against the father, the model suggested that greater exposure to violence was associated with higher levels of concern (β = 0.19, $p < 0.001$ and β = 0.17, $p < 0.01$) and disengagement (β = 0.20, $p < 0.001$ and β = 0.18, $p < 0.001$). Model fit indices were $\chi^2(8) = 5.23$, p = 0.732. The model explained 23% of the variance in CPV against the father.	CPV, with the effect of victimization at home on CPV being mediated by emotional insecurity and justification of violence.<br>There was a relationship between female sex and the use of concern and disengagement strategies within the family system. Female sex was also associated with higher justification of violent behavior.<br>**Limitations:**<br>- Correlational study, precluding causal inference;<br>- Retrospective design;<br>- Limited sample representativeness.
**Loinaz et al. (2020) [46]**	-	To analyze the existence of differences in risk factors between male and female adolescents involved in CPV.	The Guide for the Assessment of Filio-Parental Violence Risk (CPV Risk Assessment Guide).	**Descriptive analyses:**<br>- Frequency analysis and chi-square test to compare the prevalence of risk factors;<br>- Student's t-test;<br>- ROC curve analysis;<br>- Effect size analyses.	Significant differences were found in risk factors, such as escalation, which was more prevalent among boys, and issues related to self-esteem, interparental violence, cohabitation problems, and parental issues, which were more prevalent among girls. Girls experienced more domestic violence, reported significantly higher rates of CPV, exhibited greater self-esteem problems, and showed increased prevalence of interparental violence, non-CPV cohabitation problems, and	No significant differences were found in the use of violence; however, psychological abuse was slightly higher among boys towards both parents, while physical violence was more prevalent among girls. Boys and girls who perpetrated CPV showed few differences in risk factors. The results did not support higher impulsivity, single-parent family status, or substance use as distinguishing factors.

				parental conflicts. Boys revealed a higher prevalence of substance abuse.<br>Boys and girls were similar in academic/employment status and family variables. Mothers were more frequently victimized—96.4% by boys and 94.3% by girls. Fathers were victims in 51.8% of cases by boys and 45.7% by girls. Physical violence was more frequently perpetrated by boys (32.1%), while physical violence against the mother was more often perpetrated by girls (68.6%).<br>The age of onset of violence was similar between boys (M = 12.32, SD = 3.59) and girls (M = 12.46, SD = 3.03), t(71) = -0.158, p = .875. Total risk levels in the assessment were also similar, t(89) = 0.463, p = .645, d = 0.10, for boys (M = 17.76, SD = 10.1) and girls (M = 18.74, SD = 9.3). The effect size for predicting injury to mothers by male offenders was greater than 68.26% (PSTE = 0.6826). No injuries caused by female offenders to fathers were reported.	Girls were more likely to have prior problematic contexts, cohabitation problems within the family, and lower self-esteem. Additionally, 10% of girls showed a higher likelihood of being victims of bullying, intrafamilial violence, anger issues, problematic parenting styles, and non-violent conflicts between parents. Boys exhibited a higher probability of substance abuse and greater escalation of violence, as well as more antisocial behavior.<br>**Limitations**:<br>- Limited representativeness of the sample;<br>- Lack of psychometric results to compare risk factors.
**Loinaz et al. (2023) [47]**	- To compare different risk profiles of CPV perpetrators using a CPV risk assessment tool.	The Child-to-Parent Violence Risk (CPVR).	**Descriptive analyses:**<br>- Chi-square tests to compare proportions of	The age of onset of violence was similar between those who exclusively committed child-to-parent violence (specialists) and those who committed other	The overall prevalence of risk factors was higher among generalist offenders, with greater presence of domestic violence, bullying victimization, empathy

		risk and protective factors between specialists and generalists, and for sex comparisons;<br>- Frequency analysis and T-tests.	offenses (generalists) (generalists M = 12.02, SD = 3.33; specialists M = 12.74, SD = 2.65), $t(204) = -1.45$, $p = 0.111$.<br>A significantly higher prevalence was found among generalists for home victimization, bullying victimization, empathy problems, anger control difficulties, justification of violence, antisocial behavior, failure in previous interventions, parental violence, cohabitation problems, problematic parenting style, and role reversal in the family hierarchy.<br>Regarding risk profile, generalists showed significantly higher scores (M = 28.56, SD = 7.09), $t(204) = 5.81$, $p < 0.001$, indicating a worse risk profile.<br>Male and female specialists presented similar levels of risk (M = 20.50, SD = 9.23; M = 22.48, SD = 9.22), $t(130) = 1.212$, $p = 0.228$.<br>Male generalist offenders experienced more domestic violence, child-to-parent violence complaints, empathy problems, academic difficulties, antisocial behavior, failure of previous interventions, cohabitation problems, and parental personal problems. Specialists showed significantly more motivation to change ($\chi^2 = 10.105$, $p = 0.002$),	problems, anger control difficulties, justification of violence, antisocial behavior, failure of previous interventions, interparental violence, cohabitation problems, problematic parenting styles, and role reversal within the family hierarchy.<br>Generalist and specialist youths did not differ in impulsivity, substance use, age, or association with antisocial peers.<br>**Limitations**:<br>- The type of violence was not assessed;<br>- The variable used to classify perpetrators was dichotomous;<br>- Profile descriptions were not studied;<br>- Only bivariate analyses were conducted.

				future plans (χ² = 4.406, p = 0.036), and family support (χ² = 5.220, p = 0.022). Female generalists exhibited more bullying victimization, anger control problems, justification of violence, antisocial behavior, cohabitation problems, and non-violent parental conflicts. Specialists showed more future plans (χ² = 4.406, p = 0.036) and social support (χ² = 6.553, p = 0.010).	
**Maranon & Ibabe (2024) [48]**	- To identify differential psychological and clinical characteristics of adolescents who perpetrate CPV, as well as parenting practices and family functioning;<br>- To explore the best predictors related to adolescent characteristics, parenting, and family functioning to discriminate between groups.	**CPV:** Conflict Tactics Scales Child-Parents (CTS-CP) (Straus & Douglas, 2004). **Emotional Intelligence:** Trait Meta-Mood Scale (TMMS-4) (Salovey et al., 1995). **Psychological Functioning:** Outcome Questionnaire (OQ-45) (Lambert et al., 2004). **Parenting:** Parental Bonding Instrument (PBI) (Parker et al., 1979). **Family Criticism:** Family Emotional Involvement and Criticism Scale (FEICS) (Shields et al., 1992). **Family Adaptability and Cohesion:** Family Adaptability and Cohesion Evaluation Scale (FACES II) (Olson et al., 1982).	**Descriptive analyses:**<br>- Student's t-test and Chi-square test to examine group differences;<br>- Cohen's d and Cramer's V correlation coefficient to calculate the effect size of group differences.<br>**Inferential analyses:**<br>- Logistic regression; predictive analyses using Receiver	A large effect size was found for educational level (Cramer's V = 0.51) and family structure (Cramer's V = 0.41). Adolescents involved in CPV showed poorer psychological functioning (d = 0.57), lower levels of emotional repair (d = −0.37), and greater distress (d = 0.36). They also reported lower perceived maternal care (d = −1.11) and paternal care (d = −0.93), alongside higher parental overprotection (d = 0.95) and maternal overprotection (d = 0.56). Poorer family functioning was observed in the CPV group, characterized by lower family cohesion (d = −1.43), family adaptability (d = −1.24), family history knowledge (d = −0.77), and higher family criticism (d = 1.33). Family criticism, family cohesion, family history knowledge, and	Differences in Psychological and Clinical Profiles and Family Functioning.<br>Adolescents in the CPV group showed greater psychological impairment, more clinical symptoms, and lower emotional intelligence. They also exhibited reduced emotional repair capacity, indicating difficulties in interrupting and reducing negative emotional states.<br>A higher percentage of separated parents (44%) was observed in the CPV group. Parents who perpetrated violence demonstrated lower levels of care toward their children and a parenting style characterized by overprotection.

		**Family History Knowledge:** Do You Know? (DYK) (Duke et al., 2008).	Operating Characteristic (ROC) curve and Area Under the Curve (AUC) performed with NCSS 2020 software.	educational level significantly contributed to the model. The estimated model showed good fit: $\chi^2(8, n = 133) = 3.35$, $p = 0.910$. The regression model explained 64.8% of the variance. Results revealed that family criticism (OR = 1.105, $p = 0.03$) was a significant predictor of CPV.<br>Family cohesion was the strongest predictor according to the ROC curve analysis, discriminating 84% of adolescents between CPV and non-CPV groups [AUC = 0.839; 95% CI = 0.7577 – 0.895; $p < 0.001$], followed by family criticism with 83% accuracy [AUC = 0.828; 95% CI = 0.744 – 0.887; $p < 0.001$], and educational level [AUC = 0.775; 95% CI = 0.693 – 0.840; $p < 0.001$].	Adolescents involved in CPV reported poorer family functioning, including increased family criticism, low cohesion, reduced adaptability, and limited knowledge of family history. The reported family criticism reflects a lack of emotional support and mutual distrust within these families.<br>An optimal cutoff score of 17 was identified, accurately discriminating most adolescents in the current study, with specificity (77%) and sensitivity (75%) indices close to the standard threshold (80%).<br>**Limitations:**<br>- Cross-sectional study design;<br>- Use of self-report measures;<br>- Low incidence of reported abuse;<br>- Limited sample representativeness.
**Martín et al. (2022) [49]**	- To establish a psychosocial profile of adolescents and adults who have admitted to committing CPV.	**CPV**: Self-Reported Child-to-Parent Violence (Hernández, 2016).<br>**Exposure to violence**: Exposure to Violence Scale [63]. **Self-concept**: Self-Concept Scale (Garcia & Musitu, 2014).	**Descriptive analyses:**<br>- Student's t-test to analyze group differences;<br>- Cohen's d to estimate effect size.	The best predictive model for CPV included academic self-concept ($\beta = -0.149$) and family self-concept ($\beta = -0.177$), avoidant ($\beta = 0.313$) and rational problem-solving styles ($\beta = -0.239$), and a negative orientation toward problems ($\beta = -0.533$). Participants who acknowledged	The existence of a common profile of CPV perpetrators among both adolescents and adults was identified. Exposure to violence and family self-concept were the variables that most strongly defined the

		**Reactivity**: Interpersonal Reactivity Index (Davis, 1980). **Problem-solving style**: Social Problem-Solving Inventory-Revised [67].	**Inferential analyses:**<br>- Stepwise regression;<br>- Logistic regression.	having engaged in CPV exhibited a negative orientation toward problems, an avoidant style, and a less rational problem-solving style. Good model fit indices were obtained with the classical model (adjusted $R^2 = 0.255$) and with the Bayesian replication ($R^2 = 0.294$; BFM = 83.736; BF10 = 202.011).	psychosocial profile of CPV perpetrators. Academic self-concept also differentiated those who admitted to engaging in CPV from those who did not. No empathy-related variables (fantasy, perspective-taking, empathic concern, personal distress) were associated with CPV.<br>**Limitations**:<br>- Entirely male sample;<br>- Cross-sectional and retrospective study design.
**Martínez-Ferrer et al. (2020) [50]**	- To analyze the relationships between CPV, psychological distress, suicidal ideation, and family self-concept in school-aged adolescents, considering sex differences.	**Conflict Strategies:** Conflict Tactics Scales (CTS2) (Straus & Douglas, 2004).<br>**Psychological Distress:** Kessler Psychological Distress Scale (K10) (Kessler & Mroczek, 1994).<br>**Suicidal Ideation:** Suicidal Ideation Scale (Roberts, 1980).<br>**Family and Social Self-Concept:** AF-5 Scale (García & Musitu, 1999).	**Descriptive analyses:**<br>- Frequency analysis according to sex, CPV, psychological distress, suicidal ideation, and self-concept.<br>**Inferential analyses:**<br>- MANOVA;<br>- ANOVA.	Significant main effects were found for CPV [$\alpha = 0.895$, $F(8, 16210) = 159.598$, $p < 0.001$, $\eta^2 = 0.073$] and sex [$\alpha = 0.977$, $F(4, 8105) = 48.030$, $p < 0.001$, $\eta^2 = 0.023$]. A statistically significant interaction effect was observed between CPV and sex [$\alpha = 0.992$, $F(8, 16210) = 7.735$, $p < 0.001$, $\eta^2 = 0.004$].<br>Significant differences were found in psychological distress [$F(2, 8112) = 545.973$, $p < 0.001$, $\eta^2 = 0.119$], suicidal ideation [$F(2, 8112) = 318.600$, $p < 0.001$, $\eta^2 = 0.073$], family self-concept [$F(2, 8112) = 251.839$, $p < 0.001$, $\eta^2 = 0.058$], and	Levels of CPV increased as psychological distress intensified.<br>Higher involvement in CPV was associated with elevated levels of suicidal ideation, suggesting that CPV may represent a manifestation of maladjustment in youth linked to psychological suffering.<br>CPV increased as family and social self-concept decreased. Females exhibited higher levels of psychological distress and suicidal ideation, as well as lower family self-concept scores.<br>**Limitations:**

					social self-concept [F(2, 8111) = 32.288, $p < 0.001$, $\eta^2 = 0.008$]. There was a statistically significant three-way interaction among CPV, sex, and psychological distress [F(2, 8108) = 17.049, $p < 0.001$]. When CPV levels were low, moderate, or high, females reported higher psychological distress scores. A significant interaction effect was also found between CPV, sex, and suicidal ideation [F(2, 8108) = 14.311, $p < 0.001$, $\eta^2 = 0.004$], as well as between CPV, sex, and family self-concept [F(2, 8108) = 9.396, $p < 0.001$, $\eta^2 = 0.002$].	- Cross-sectional study design;<br>- Limited sample representativeness;<br>- Use of self-report measures.
**Nam et al. (2022) [51]**	-	To investigate the relationship between family violence initiated by adults and CPV, focusing on the moderating effect of peer	**CPV:** Conflict Tactics Scales (CTS) (Straus, 1979).<br>**Child Abuse:** Parent–Child Conflict Tactics Scales (PCCTS) [104].<br>**Child Exposure to Intimate Partner Violence:** Conflict Tactics Scales (CTS) (Straus, 1979).<br>**Peer Attachment:** Inventory of Parent and Peer Attachment (IPPA) [75].	**Descriptive analyses:**<br>- Frequency analysis of the sample and prevalence of CPV and victimization.<br>**Inferential analyses:**<br>- ANOVA;<br>- Hierarchical multiple regression analysis.	17.1% of the youths reported perpetrating CPV. Psychological CPV was reported by 16.2%, and physical CPV by 6%.<br>66.8% reported having experienced child abuse by their parents, with 35.6% suffering physical abuse and 34.4% witnessing intimate partner violence.<br>The first model was statistically significant, explaining 16% of the variance in CPV (F(8, 674) = 17.30, $p < 0.001$). Child abuse was significantly associated with CPV (p <	Child maltreatment significantly increases CPV. Adolescents with low self-esteem exhibited higher levels of CPV. Peer attachment moderated the effect of child abuse on CPV; specifically, a high level of peer attachment attenuated the impact of child abuse on CPV.<br>**Limitations:**<br>- Cross-sectional study design;<br>- Limited generalizability of findings;<br>- Use of self-report measures.

	attachment on this relationship.			0.001). Self-esteem was also associated with CPV ($p < 0.04$). The second model explained 25% of the variance in CPV ($F(10, 672) = 23.07$, $p < 0.001$). A significant interaction between child abuse and peer attachment was observed ($B = –0.22$, $p < 0.001$).	
**Navas-Martínez & Cano-Lozano (2022) [52]**	- To identify the typology of specialist offenders versus generalist offenders in CPV;<br>- To examine whether these groups differ in their characteristics.	**CPV:** Child-to-Parent Violence Questionnaire, Adolescents Version (CPV-Q-A) (Contreras & Cano-Lozano, 2019).<br>**Peer Violence:** European Bullying/Cyberbullying Intervention Project Questionnaires (EBIP-Q and ECIP-Q) (Ortega-Ruiz & Del Rey, 2016).<br>**Emotional Intelligence:** Wong–Law Emotional Intelligence Scale (WLEIS) (Wong & Law, 2002).<br>**Resilience:** Connor–Davidson Resilience Scale, Short Version (CD-RISC-10) (Connor & Davidson, 2003).<br>**Parental Attachment:** Attachment Representation Questionnaire, Short Version (CAMIR-R) (Pierrehumbert et al., 1996).<br>**Exposure to Violence:** Violence Exposure Scale (VES) (Calvete et	**Descriptive analyses:**<br>- Chi-square test to examine differences in the distribution of perpetrators according to age and sex;<br>- Cohen's d to estimate effect size;<br>- Mann-Whitney U test to compare differences between groups.<br>**Inferential analyses:**<br>- Logistic regression;<br>- Wald test to assess the significance of the model	No sex differences were found between specialist and generalist perpetrators ($\chi^2(1, N = 1558) = 0.06$, $p = 0.799$). A higher proportion of generalists (64.8%) were aged between 14 and 16 years compared to specialists (57.2%). Conversely, more specialists (42.9%) than generalists (35.2%) were aged 12–13 and 17–18 years.<br>Generalists scored higher on CPV against both parents (CPV mother: Cohen's $d = 0.60$; CPV father: $d = 0.54$), displaying elevated scores across all types of CPV and both reactive and proactive motives. They also scored lower in emotional awareness, assimilation, and regulation, as well as resilience capacity. Furthermore, generalists exhibited higher insecure attachment scores (preoccupied,	The existence of two distinct types of CPV perpetrators was confirmed. The majority of CPV occurs within a generalized pattern of violent behavior. Specialists and generalists differed significantly on all variables except for sex, control/dominance towards the father, the ability to recognize others' emotions, and witnessing physical violence between parents.<br>Generalist perpetrators engaged in violence across multiple contexts and exhibited higher levels of violent behavior towards both parents, which was more severe than that of specialists. Generalists also displayed greater proactive CPV. Moreover, generalists showed lower emotional intelligence and more insecure emotional

		al., 2014).	parameters.	avoidant, and traumatized). Regarding family functioning, generalists showed higher parental permissiveness and lower valuation of parental authority (effect sizes d < 0.20).<br>The logistic regression model correctly classified 78.2% of cases and was statistically significant ($\chi^2(4, N = 1559) = 173.02, p < 0.001$, Nagelkerke $R^2 = 0.164$), explaining 16.4% of the variance in perpetrator type.	attachment to their parents.<br>**Limitations**:<br>- Cross-sectional study design;<br>- Limited sample representativeness;<br>- Reliance on self-report measures.
**Navas-Martínez & Cano-Lozano (2023) [53]**	- To explore the frequency of different types of adverse childhood experiences (ACEs) and cumulative ACEs in adolescents who have perpetrated child-to-parent violence, compared to those who have not.<br>- To analyze differences among perpetrators with varying levels of cumulative ACEs in parental attachment, resilience, and emotional intelligence.	**CPV:** Measured using the Child-to-Parent Violence Questionnaire (CPV-Q; Contreras et al., 2019).<br>**Exposure to Family Violence:** Assessed with the Violence Exposure Scale (VES; Calvete et al., 2014).<br>**Peer Violence Exposure:** Evaluated using the European Bullying Intervention Project Questionnaire (EBIP-Q; Brighi et al., 2012) and the European Cyberbullying Intervention Project Questionnaire (ECIP-Q; Ortega-Ruíz et al., 2016).<br>**Adverse Childhood Experiences (ACEs):** Measured with an ad hoc Adverse Childhood Experiences Questionnaire (Téllez et al., 2022).<br>**Attachment:** Assessed by the Attachment Representations	**Descriptive analyses:**<br>- Frequency analysis of adolescents who experienced adverse childhood experiences (ACEs).<br>- Chi-square tests and Phi coefficient (ϕ) to compare those who did not experience ACEs.<br>**Inferential analyses:**<br>- Hierarchical cluster analysis to identify groups based on cumulative ACEs.	A higher prevalence of adverse childhood experiences (ACEs) was found among adolescents who perpetrated CPV. Specifically, 39.8% and 42.3% reported direct abuse by the mother and father, respectively, while 1.2% and 5.7% experienced maternal and paternal neglect. Peer abuse ranged between 50% and 77%. A greater proportion of cumulative ACEs was observed among youth who engaged in CPV (88%) compared to those who did not.<br>Cluster analysis identified a high ACEs group comprising 274 perpetrators (18.9%) (M = 7.9, SD =	Adverse life experiences related to abuse were frequent among adolescents who perpetrated CPV, with 39.8% reporting abuse by the mother, 42.3% by the father, 16.7% witnessing paternal abuse against the mother, and 50.9% experiencing cyberbullying.<br>Twenty-two percent of CPV perpetrators experienced cumulative victimization (parental and peer abuse) and were generally characterized by insecure parental attachment, lower resilience, and reduced emotional intelligence.<br>Significant differences were

	Questionnaire (CAMIR-R; Pierrehumbert et al., 1996).<br>**Resilience:** Measured with the Connor and Davidson Resilience Scale (CD-RISC-10; Connor & Davidson, 2003).<br>**Emotional Intelligence:** Evaluated using the Wong and Law Emotional Intelligence Scale (WLEIS; Wong & Law, 2002).	- Analysis of variance (ANOVA) to examine differences between clusters in parental attachment, resilience, and emotional intelligence.<br>- Cohen's d to estimate effect sizes.	1.2, range: 7–12), a low ACEs group with 1,003 perpetrators (69.1%) ($M = 3.9$, $SD = 1.3$, range: 2–6), and a no ACEs group including 174 perpetrators (12%) ($M = 0.7$, $SD = 0.4$, range: 0–1). Significant intergroup differences were found in the distribution of ACEs ($\chi^2(24, N = 1{,}451) = 2902.0$, $p < 0.001$, $\phi = 1.4$) and in mean ACEs scores ($F = 4732.0$, $p < 0.001$).<br>Both cumulative ACEs groups exhibited lower scores in secure parental attachment and higher scores in insecure parental attachment compared to the no ACEs group. The high ACEs group showed significantly lower resilience compared to the low and no ACEs groups. Regarding emotional intelligence, both cumulative ACEs groups had lower scores.<br>These results indicate that CPV is positively associated with adverse life experiences and negatively associated with secure parental attachment, resilience, and emotional intelligence.	found in traumatized attachment and emotional assimilation and regulation, suggesting that perpetrators with cumulative adverse experiences tend to develop mental representations of their parents, themselves, and the world based on a lack of affection, which is associated with difficulties in emotional regulation.<br>Adverse childhood experiences directly predisposed adolescents to CPV. Negative associations were found between CPV and secure parental attachment, resilience, and emotional intelligence.<br>**Limitations**:<br>- Cross-sectional study design;<br>- Use of self-report measures;<br>- Limited generalizability of findings;<br>- Greater emphasis was placed on familial adverse experiences compared to extrafamilial ones.

**Navas-Martínez et al. (2023) [54]**	- To analyze whether family victimization is related to CPV through insecure parental attachment;<br>- To examine the relationship between different insecure attachment styles and CPV;<br>- To analyze the mediating role of insecure attachment in the relationship between both types of family victimization and CPV.	**CPV:** Child-to-Parent Violence Questionnaire, young version (CPV-Q) [19].<br>**Familiar victimization**: *Violence Exposure Scale* (VES) [63].<br>**Parental attachment:** *Attachment Representation Questionnaire, short version* (CaMir-R) (Pierrehumbert et al., 1996).	Software used R version 4.1.3 and Jamovi version 2.2.5.<br>**Psychometric analysis**:<br>- Confirmatory factor analysis (CFA).<br>**Descriptive analyses**:<br>- Frequency analysis, including assessment of normality using Mardia's test.<br>**Inferential analyses:**<br>- Linear regression analysis.	Results indicate that insecure attachment, specifically the traumatized style, significantly mediates the relationship between family victimization and CPV.<br>Significant positive correlations were observed among all variables. CPV against the father and mother was related to direct family victimization ($r = 0.355$, $r = 0.385$) and vicarious victimization ($r = 0.169$, $r = 0.162$), respectively. Family victimization was associated with attachment styles characterized as preoccupied ($r = 0.198$, $r = 0.116$), avoidant ($r = 0.263$, $r = 0.211$), and traumatized ($r = 0.373$, $r = 0.539$), which were also related to CPV against the father ($r = 0.168$, $r = 0.206$, $r = 0.287$) and mother ($r = 0.179$, $r = 0.226$, $r = 0.307$).<br>In the model of child-to-father violence, the total effect of the relationship between direct family victimization and CPV was significant ($\beta = 0.34$, $p < 0.001$), attributable to the direct effect between these variables ($\beta = 0.29$, $p < 0.001$) and the indirect effect via traumatized attachment ($\beta = 0.03$, $p = 0.006$).<br>Similarly, in the model of child-to-mother violence, the total effect of direct family victimization on CPV was significant ($\beta = 0.38$, $p < 0.001$),	A stronger relationship was observed between CPV and direct family victimization than between CPV and vicarious victimization. Positive and significant correlations were found between family victimization and preoccupied, avoidant, and traumatized attachment styles, with traumatized attachment showing the strongest association with both direct and vicarious family victimization.<br>The parental attachment styles that explained CPV differed depending on whether the violence was directed toward fathers or mothers. Traumatized attachment was associated with CPV against both parents, while avoidant attachment was only associated with CPV against mothers.<br>Direct victimization was directly related to CPV and indirectly related through traumatized attachment. The relationship between direct victimization and CPV was better explained by the mediating effect of traumatized

				due to the direct effect (β = 0.32, $p < 0.001$) and the indirect effect mediated by traumatized attachment (β = 0.04, $p < 0.001$).	attachment.<br>**Limitations:**<br>- Cross-sectional and retrospective study design;<br>- Use of self-report measures;<br>- Limitations regarding the generalizability of the findings;<br>- Cautious interpretation advised due to the reliability of the avoidant attachment scale.
**Padilla-Falcón & Moreno-Manso (2019) [55]**	- To analyze the psychosocial and clinical characteristics of minors reported for CPV;<br>- To examine the profile of their families and the judicial measures imposed.	Data were obtained from juvenile prosecution files of the Public Prosecutor's Office of the province of Badajoz, covering the years 2012 to 2017.<br>**Personality**: Personality Assessment Inventory for Adolescents (PAI-A) (Morey, 2007).	**Descriptive analysis**:<br>- Frequency analysis of variables, significant differences in PAI-A scales/subscales according to sex and age, and judicial measures according to sex and age.	Both males and females predominantly committed aggression against the mother, followed by aggression against both parents. A higher prevalence of conflictual social relationships was observed in males and youths aged 16–17 years.<br>Family composition showed that 48.3% were two-parent families, 33.8% were single-parent families, and 17.9% were reconstituted families.<br>The predominant parental style was paternal in 43.4% and maternal in 73.1%. Substance use was reported in 9% of fathers and 6.9% of mothers. Psychological disorders were absent in 79.3% of the parents. Regarding the youths,	Aggressive behavior directed toward both parents increases with age.<br>Boys aged 16–17 years appeared to be more well-adjusted.<br>Sporadic substance use was present in 60% to 77% of cases.<br>Girls scored higher on anxiety, post-traumatic stress-related disorders, paranoia, and borderline traits. In terms of anxiety, they experienced greater worry, sensitivity, apprehension, and tension.<br>Boys obtained higher scores on phobias, activity level, social indifference, self-harm, antisocial traits, egocentrism, drug problems, and physical aggression. They also showed a history of antisocial behavior and may have exhibited some conduct disorder.<br>The most frequently imposed

			elevated mean scores were found for anxiety (M=56.43, SD=6.81), depression (M=56.04, SD=4.34), borderline traits (M=56.06, SD=4.40), and drug problems (M=57.39, SD=6.01), although these were not of high clinical relevance. Above-average scores were noted in cognitive anxiety (M=59.02, SD=5.20), emotional anxiety (M=59.11, SD=4.30), and physiological anxiety (M=60.64, SD=5.72). Average scores were found in cognitive depression (M=57.92, SD=4.69) and emotional depression (M=57.18, SD=5.67). Within borderline traits, self-harm showed above-average values (M=57.81, SD=5.01). In paranoia, resentment was notable (M=60.69, SD=5.00). Among antisocial traits, antisocial behavior stood out (M=56.28, SD=5.58). Regarding aggression, the highest values were found in physical aggression (M=56.88, SD=6.99) and aggressive attitude (M=54.97, SD=4.85).<br>Youths aged 14–15 years showed higher scores in somatoform disorders, anxiety-related	judicial measures were educational group programs and probation.<br>**Limitations**:<br>- Study conducted within a legal context and limited to a single province, which restricts the generalizability of the results.

				disorders, schizophrenia, borderline traits, antisocial traits, and alcohol problems. Youths aged 16–17 years exhibited more pronounced anxiety and drug-related problems. No significant sex differences were found regarding judicial measure orientation ($t(103.24) = -0.291$, $p = 0.771$).	
**Palanques et al. (2022) [56]**	- To analyze the criminological profile of juveniles who have committed CPV compared to juveniles who have committed other types of crimes	Risk of recidivism: Youth Level of Service/Case Management Inventory (YLS/CMI).<br>Interviews with youths and their families.	**Descriptive analyses:**<br>**-** Chi-square tests<br>**Variance analyses:**<br>**-** ANOVA	The majority of the youth were male (63.4%). Higher recidivism rates were observed among youth who committed CPV (60.1%), as well as a greater number of crimes committed (2.54 on average). The CPV group presented a moderate risk of recidivism (68.1%), while the comparison group showed a low risk (55.5%), $\chi^2(2) = 50.45$, $p < .001$. The largest differences between risk factors were found in the subscales related to family, parenting, personality/behavior, and substance abuse. Regarding the sanctions applied, youth in the CPV group were more frequently subjected to supervised release (46.6%) and	The youth in the CPV group were predominantly male, with a mean age of 16 years. A high percentage of recidivism and number of crimes committed were observed in the CPV group during the study, possibly due to the impulsivity they exhibit. Elevated averages were found in substance abuse and adverse family circumstances, which may reflect underlying psychological distress.<br>**Limitations**:<br>- Data were collected from a single Spanish province, limiting the generalizability of the results;<br>- Reluctance of parents to report CPV.

				confinement (21.6%).	
**Rosado et al. (2017) [57]**	- To analyze the role of psychopathological symptomatology in youth involved in CPV;<br>- To examine the moderating effect of sex on these psychopathological symptoms.	**CPV**: Child-to-Parent Aggression Questionnaire (CPAQ) [4].**Symptoms**: Symptom Checklist-90-Revised (SCL-90-R) (Derogatis, 2001).	**Descriptive analyses:**<br>- Frequency analysis of sociodemographic variables, SCL-90-R scale scores, types of CPV, substance use, and the importance attributed to studies.<br>**Inferential analyses:**<br>- Multiple linear regression;<br>- Hierarchical multiple linear regression.	Psychological CPV was the most frequent, occurring against mothers (M = 0.59, SD = 0.50) and fathers (M = 0.49, SD = 0.49). More violent behaviors were directed towards mothers than fathers (M = 0.44, SD = 0.39; M = 0.36, SD = 0.37, respectively). A total of 21.7% admitted to committing severe psychological aggression against the father and 27% against the mother.<br>For total CPV against the father, the multiple linear regression model was significant, with an adjusted $R^2 = 0.21$, $F(13, 806) = 16.96$, $p < 0.001$. The predictors of CPV against the father included the importance the youth attributed to their studies ($\beta = -0.156$, $p < 0.001$), drug use ($\beta = 0.141$, $p < 0.001$), and age ($\beta = 0.067$, $p < 0.05$). Additionally, the paranoid ideation scale ($\beta = 0.116$, $p < 0.05$), depression ($\beta = -0.179$, $p < 0.05$), and hostility ($\beta = 0.284$, $p < 0.001$) were significant predictors.<br>For psychological CPV against the father, the multiple linear regression model had an adjusted $R^2 = 0.186$, $F(13, 813) = 15.56$, $p < 0.001$. Significant predictors were importance given to studies ($\beta = -0.136$, $p < 0.001$), drug or alcohol use ($\beta = 0.120$, $p < 0.001$), age ($\beta =$	The importance attributed to studies acted as a protective factor against the perpetration of CPV.<br>Drug use by participants was related to all types of CPV except physical violence.<br>There was a significant association between total and psychological CPV against the mother and sex, with girls showing a higher likelihood of committing these violent acts.<br>Hostility was positively correlated with all types of CPV toward both parents, indicating that irritable and bad-tempered youths are more likely to respond to family conflicts with violence.<br>Depression showed a negative relationship with all types of CPV.<br>Paranoid ideation was related to psychological and total CPV against both parents.<br>No significant relationship was found between anxiety and the various forms of CPV<br>Psychopathological

0.067, $p < 0.05$), paranoid ideation ($\beta = 0.136$, $p < 0.05$), depression ($\beta = -0.161$, $p < 0.01$), and hostility ($\beta = 0.278$, $p < 0.001$).<br>Physical CPV against the father showed an adjusted $R^2 = 0.040$, $F(13, 830) = 3.15$, $p < 0.001$, with psychoticism ($\beta = 0.128$, $p < 0.05$) and hostility ($\beta = 0.091$, $p < 0.05$) as significant predictors.<br>Economic CPV against the father was related to the importance given to studies ($\beta = -0.177$, $p < 0.001$), drug use ($\beta = 0.208$, $p < 0.001$), and paternal drug use ($\beta = 0.066$, $p < 0.05$), as well as hostility ($\beta = 0.210$, $p < 0.001$), interpersonal sensitivity ($\beta = 0.131$, $p < 0.05$), psychoticism ($\beta = 0.120$, $p < 0.05$), and depression ($\beta = -0.198$, $p < 0.05$).<br>Regarding total CPV against the mother, the multiple linear regression model revealed an adjusted $R^2 = 0.26$, $F(13, 811) = 22.27$, $p < 0.001$. Significant predictors included female sex ($\beta = 0.084$, $p < 0.05$), importance given to studies ($\beta = -0.159$, $p < 0.001$), drug use ($\beta = 0.177$, $p < 0.001$), and age ($\beta = 0.075$, $p < 0.05$), along with paranoid ideation ($\beta = 0.106$, $p < 0.05$), depression ($\beta = -0.173$, $p < 0.01$), and hostility ($\beta = 0.335$, $p < 0.001$).	symptomatology demonstrated greater predictive power in youths aged 16 to 18 years. The effects of psychopathology on CPV perpetration toward father and mother depended on the aggressor's sex.<br>In total CPV against the father, interpersonal sensitivity was significantly associated in males. Obsessive symptoms were more strongly related to total CPV against the father among females.<br>In total CPV against the mother, interpersonal sensitivity showed a positive association in males.<br>**Limitations**:<br>- Correlational study design;<br>- Low number of minors who admitted to severe behaviors;<br>- Family variables were self-reported by participants.

					For psychological CPV against the mother, significant predictors included sex (β = 0.089, p < 0.01), importance given to studies (β = -0.143, p < 0.001), drug use (β = 0.156, p < 0.001), age (β = 0.085, p < 0.01), interpersonal sensitivity (β = 0.112, p < 0.05), depression (β = -0.154, p < 0.01), and hostility (β = 0.323, p < 0.001). This model explained 23% of the variance in psychological CPV against the mother, F(13, 832) = 20.39, p < 0.001. Physical CPV against the mother showed an adjusted R^2 = 0.075, F(13, 841) = 6.33, p < 0.001, and was related to importance given to studies (β = -0.096, p < 0.05) and hostility (β = 0.199, p < 0.001). Economic CPV against the mother was associated with importance given to studies (β = -0.137, p < 0.001), drug use (β = 0.179, p < 0.001), hostility (β = 0.220, p < 0.001), psychoticism (β = 0.137, p < 0.05), and depression (β = -0.182, p < 0.01).	
**Sasaki et al. (2021) [58]**	-	To evaluate the clinical characteristics of child and adolescent psychiatric	**CPV:** Individual interviews with patients and their parents. **Psychiatric diagnosis:** Based on the DSM-5 criteria. **Anxiety disorders**: Spence Children's Anxiety Scale (SCAS)	Analyses were conducted using Easy R Package version 1.40. **Descriptive analyses:**	TABS, ADHD-RS, and ODBI scores, male sex (70.6%), antisocial behavior (42.2%), history of abuse (33%), experience of physical abuse (18.3%), psychological abuse (16.5%), and witnessing	The proportion of children who experienced physical abuse, psychological abuse, and witnessed interparental violence was significantly higher among children with CPV.

	patients who engage in CPV.	(Spence, 1998).<br>**Depression: Depression** Self-Rating Scale for Children (DSRS) (Birleson, 1981).<br>**Autism**: Tokyo Autistic Behavior Scale (TABS) (Tachimori et al., 2000).<br>**ADHD**: ADHD Rating Scale (ADHD-RS) (DuPaul & Power, 1998).<br>**Oppositional behavior**: Oppositional Defiant Behavior Inventory (ODBI) (Burns et al., 2009).	- Pearson's Chi-square test was used to compare proportions of variables between groups.<br>- Mann-Whitney U test was used to compare continuous variables between groups.<br>**Inferential analyses:**<br>- Univariate and multivariate logistic regression analyses were performed.	violence (11%) were significantly higher in the CPV group. Neurodevelopmental disorders were more prevalent in the CPV group. No significant differences were found in anxiety and depression disorders. Multivariate logistic regression analysis revealed that antisocial behavior, ADHD symptom scores, and history of abuse were independently associated with CPV.	No significant differences were found in depression. A higher proportion of patients with neurodevelopmental disorders was observed in the CPV group, with an increased likelihood of ADHD characteristics being associated with this type of violence, as well as impulsivity. Significant differences were found between TABS and ADHD-RS scores. CPV was not significantly correlated with school refusal.<br>**Limitations**:<br>- Ascertainment bias;<br>- Information based on patient self-reports and parental reports;<br>- Selection bias and small sample size.
**Sheed et al. (2023) [59]**	- To examine how the characteristics of youth who perpetrate CPV differ between early adolescence, late adolescence, and early adulthood.	Data sourced from the Victoria Police database, State of Victoria, Australia.	**Descriptive analyses:**<br>- Frequency analysis of the sample by age and sex;<br>- Chi-square test.<br>**Inferential analyses:**<br>- Multiple comparisons analyses with	Intimate partner and parent abuse were the most common types of abuse.<br>Victims were predominantly female, while perpetrators were mostly male.<br>No significant age-related differences were found. Age-related differences were observed in substance abuse, unemployment, and CPV. No significant age differences were found in recidivism	The majority of CPV perpetrators were male and targeted female victims. Young adult CPV perpetrators were predominantly male and focused on male victims. High rates of unemployment/school dropout and substance abuse were observed among CPV perpetrators in late adolescence

			Bonferroni-Holm correction for sociodemographic, psychosocial, and victim characteristics;<br>- Kaplan-Meier survival curve analysis.	rates.<br>Adult CPV perpetrators were more likely to be male and to have victims of the same sex. In early adolescence, females were more likely to engage in CPV and to have victims of the same sex.<br>High rates of unemployment, school dropout, and substance abuse were reported.<br>Results indicate that mental health problems are more prevalent among young CPV perpetrators.	and young adulthood. Substance use levels were higher among CPV perpetrators compared to the general community.<br>Mental health needs and victimization in family violence occurred at elevated rates across all age groups. Mental health problems were more prevalent in young CPV perpetrators than in the general population.<br>**Limitations**:<br>- Use of police records of reported cases, limiting comparison with other studies;<br>- Social reluctance to report CPV cases;<br>- Absence of a comparison group, restricting understanding of how characteristics may correlate with CPV risk.
**Suárez-Relinque et al. (2020) [60]**	- Conduct a psychosocial analysis of CPV in a school sample, considering individual and family variables according to sex.	**Psychological distress:** Kessler Psychological Distress Scale (K10) (Kessler & Mroczek, 1994).<br>**Problematic social media use:** Adolescence Scale (Martínez-Ferrer et al., 2018).<br>**Non-conformist social perception:** Reputation Enhancement Scale (RES)	**Descriptive analyses:**<br>- Frequencies were calculated for categorical variables;<br>- Student's *t*-tests were conducted to compare group means;	Girls scored significantly higher on overall CPV, psychological distress, and problematic social media use, whereas boys scored higher on physical CPV and nonconformist attitudes. Significant sex differences were found in CPV levels.<br>Strong positive correlations	Girls exhibited higher levels of CPV, psychological distress, problematic social media use, open communication with mothers, and problematic communication with both parents. Boys scored higher on nonconformist attitudes and open communication with fathers. The variables with the greatest predictive importance were

	(Carroll et al., 1999).<br>**Parent-adolescent relationship:** Parent-Adolescent Communication Scale (PACS) (Barnes & Olson, 1982).<br>**CPV:** Conflict Tactics Scale (CTS2) (Straus & Douglas, 2004).	- Pearson correlation coefficients were computed to examine bivariate associations between continuous variables;<br>- Levene’s test was used to assess the homogeneity of variances across groups;<br>- The Kolmogorov-Smirnov test was applied to verify the normality of data distributions.<br>**Inferential analyses:**<br>- Stepwise multiple regression analyses were performed to identify significant predictors of CPV, including psychological distress, problematic social media use, social perception, and parent-adolescent	emerged between overall CPV and physical CPV ($r = 0.723$), psychological CPV ($r = 0.888$), nonconformist attitudes ($r = 0.388$), and psychological distress ($r = 0.372$).<br>Hierarchical multiple regression models indicated that problematic social media use alone significantly predicted CPV, $F(1, 3729) = 438.525$, $p < 0.001$, explaining 10.5% of the variance. Adding nonconformist attitudes improved the model, $F(2, 3728) = 461.540$, $p < 0.001$, increasing explained variance to 19.8%. Incorporating psychological distress further enhanced the model, $F(3, 3727) = 415.538$, $p < 0.001$, accounting for 25.1% of CPV variance. In the final model, problematic social media use ($\beta = 0.129$, $p < 0.001$), nonconformist attitudes ($\beta = 0.242$, $p < 0.001$), psychological distress ($\beta = 0.190$, $p < 0.001$), open communication with mother ($\beta = -0.047$, $p < 0.05$), problematic communication with mother ($\beta = 0.074$, $p < 0.001$), open communication with father ($\beta = -0.070$, $p < 0.001$), and problematic	nonconformist attitudes, problematic social media use, and psychological distress. Symptoms of stress and depression during adolescence increase the likelihood of aggression toward parents or family authority figures. Problematic communication significantly predicted increases in CPV directed at fathers, while open communication predicted decreases in CPV.<br>**Limitations:**<br>- Cross-sectional study design;<br>- Large sample with a higher prevalence of verbal violence, consistent with existing literature, which may influence overall CPV rates;<br>- Sample comprised mainly of mid-adolescents, the age group in which most CPV cases are reported.

			relationship variables;<br>- Confirmatory factor analysis (CFA) was conducted to validate the measurement models of the scales used.	communication with father (β = 0.112, $p < 0.001$) all significantly contributed to predicting CPV, $F(7, 3723) = 209.746$, $p < 0.001$, explaining 28.3% of the variance.	
**Suárez-Relinque et al. (2023) [61]**	- To examine the relationship between involvement in CPV and the development of emotional loneliness, suicidal ideation, and alexithymia, with a focus on sex differences.	**CPV:** Conflict Tactics Scale (CTS2).<br>**Emotional loneliness:** Loneliness Scale *(UCLA)* [109].<br>**Suicide ideation**: Suicide Ideation Scale [110].<br>**Emotional Recognition and Expression**: *Toronto* Alexithymia Scale (TAS-20) [11].	**Descriptive analyses:**<br>- Frequency analysis and distribution according to sex;<br>- Correlation analysis of the studied variables.<br>**Inferential analyses:**<br>- MANOVA;<br>- Partial eta squared (η^2) to estimate effect size;<br>- ANOVA	Significant differences were found in the main effects of CPV, $\Lambda = .92$, $F(6, 3840) = 28.39$, $p < .001$; and sex, $\Lambda = .96$, $F(3, 1920) = 25.37$, $p < .001$. A significant interaction effect between CPV levels and sex was also observed, $\Lambda = .99$, $F(6, 3840) = 2.38$, $p < .05$.<br>Significant differences were found in emotional loneliness, $F(2, 1925) = 36.08$, $p < .001$; suicidal ideation, $F(2, 1925) = 42.92$, $p < .001$; and alexithymia, $F(2, 1925) = 70.15$, $p < .001$.<br>Significant sex differences emerged in emotional loneliness, $F(1, 1926) = 4.97$, $p < .01$; suicidal ideation, $F(1, 1926) = 28.32$, $p < .001$; and alexithymia, $F(1, 1926) = 89.01$, $p < .001$, with females exhibiting higher levels of emotional loneliness, suicidal ideation, and alexithymia.	The higher the occurrence of CPV, the greater the levels of emotional loneliness, suicidal ideation, and alexithymia. Adolescents with higher CPV involvement feel more emotionally lonely, have a higher likelihood of suicidal ideation, and exhibit increased alexithymia. Elevated levels of alexithymia may be related to greater experiences of physical and sexual victimization.<br>**Limitations**:<br>- Non-clinical sample;<br>- Sample homogeneity

				A statistically significant interaction was found between sex, alexithymia and CPV levels, $F(2, 1922) = 6.61$, $p < .01$.	
**Kennedy et al. (2010) [45]**	- To determine whether juvenile offenders who have committed CPV differ from those who have not committed this type of crime.<br>- To explore the clinical and adaptive characteristics of juvenile CPV offenders with respect to sociodemographic differences, psychological functioning, exposure to domestic violence, and domestic victimization.	**Clinical and adaptive behavior of youth:** The Behavior Assessment System for Children-Self Report of Personality (SRP) [98].<br>**Intelligence***:* Kaufman Brief Intelligence *Test (K-BIT).*<br>**Cognition***:* Wide Range Achievement Test, Third Edition (WRAT-III).	**Descriptive analysis:**<br>- Chi-square test;<br>- T test.<br>**Factorial analysis**	Regarding ethnicity, 49.5% were White and 50.5% were Black in the CPV group. In the Non-CPV group, 43.4% were White and 56.6% were Black. There was a significant difference between groups regarding ethnicity for females, $\chi^2(1, N=52) = 4.94$, $p = 0.026$, $\phi = 0.31$.<br>The mean age at first detention for the total sample was 13.32 years (SD = 1.88).<br>13.3% of the total sample had previously been hospitalized for psychiatric/psychological reasons, with a higher percentage in the CPV group (20%) than in the Non-CPV group (7.2%), $\chi^2(1, N=211) = 7.48$, $p = .006$, $\phi = .19$.<br>21% of the total sample had taken psychiatric medication, with a higher proportion of youths in the CPV group (29%) compared to the Non-CPV group (13.6%).<br>10.9% of the total sample had attempted suicide, with the CPV group more likely to report	This suggests that a large proportion of youth involved with the justice system come from single-parent households. Juvenile offenders who commit CPV reported more relational difficulties with their parents. They also showed a higher likelihood of having been arrested for other violent crimes and engaging in illegal behaviors compared to their offending peers. Therefore, CPV may not only be the result of dysfunctional family relationships but also an expression of broader behavioral problems associated with conduct disorder.

				suicidal ideation (19%) than the Non-CPV group (3.6%), $\chi^2(1, N=211) = 12.84, p < .001, \phi = .25$.<br>In the total sample, 29.5% of youths described poor family relationships. The CPV group was more likely to report poor family relationships (44.4%) compared to the Non-CPV group (16.25%), $\chi^2(1, N=210) = 20.04, p < .001, \phi = .31$.<br>The majority of youths in the total sample (62.4%), including 64.9% of the CPV group and 60.3% of the Non-CPV group, reported having peer relationships with individuals who committed crimes.<br>36% of the total sample, 39% of the CPV group, and 33.3% of the Non-CPV group lived in single-parent families with the biological mother as the main caregiver.<br>51.7% of the total sample reported having been held back in school, with 51% in the CPV group and 52.3% in the Non-CPV group.	
**Zuñeda et al. (2016) [62]**	- To analyze differences in sociodemographic and family characteristics, mental	**CPV**: Conflict Tactics Scales – Child to Parents (CTS-CP) [61].<br>**Aggressiveness**: Aggression Questionnaire (AQ) [112].	**Descriptive analyses:**<br>- Assessment of normality and	The most significant interparental conflict was found in the CPV group across the three CPIC-VER scale factors: Conflict Properties,	Youth involved in CPV exhibited higher levels of physical aggression and anger. Differences in anger levels, but

	health, and aggressiveness among adolescents who commit CPV.	**Psychological Stress**: Brief Symptom Inventory-18 (BSI-18) [113]. **Family Adaptation**: Family Adaptability and Cohesion Evaluation Scale (FACES II) [114]. **Interparental Conflict from Child's Perspective**: Children's Perception of Interparental Conflict Scale (CPIC-VER) [115] **Family Communication**: Family Communication Scale (FCS-VE) [116].	distribution of the study variables;<br>- Student's t-test and Chi-square test to examine significant differences.<br>**Inferential analyses:**<br>- ANOVA;<br>- Multiple linear regression analysis.	$F(1, 113) = 26.05$, $p < 0.0001$, $\eta^2 = 0.19$; Threatening Experience or Appraisal, $F(1, 113) = 5.8$, $p < 0.018$, $\eta^2 = 0.05$; and Self-Blame, $F(1, 113) = 33.47$, $p < 0.0001$, $\eta^2 = 0.23$. Youth who exhibited CPV demonstrated higher levels of aggression.<br>The final model, consisting of physical aggression, low family cohesion, and a high sense of self-blame regarding interparental conflict, explained 55% of the variance in overall CPV.	not hostility, may indicate that the emotional dimension of aggression is more relevant than the cognitive dimension in violent behavior. No significant differences were found in anxiety, somatization, panic, or depression.<br>Family functioning was perceived as less cohesive and adaptable in the CPV group. Parental marital conflicts were perceived as more intense, frequent, and prolonged among youth in the CPV group, who also felt less capable of managing the marital conflict and more involved in it.<br>**Limitations:**<br>- Cross-sectional study design;<br>- Intentional sampling method.
